# DNA Methylation in Urine and Feces Indicative of Eight Major Human Cancer Types Globally

**DOI:** 10.3390/life15030482

**Published:** 2025-03-17

**Authors:** Melanie Engstrom Newell, Ayesha Babbrah, Anumitha Aravindan, Raj Rathnam, Rolf U. Halden

**Affiliations:** 1 Biodesign Institute, Arizona State University, Tempe, AZ 85281, USA; melanie.e.newell@asu.edu (M.E.N.);; 2 Biodesign Center for Environmental Health Engineering, Tempe, AZ 85281, USA; 3 School for Engineering of Matter, Transport and Energy, Arizona State University, Tempe, AZ 85281, USA; 4 Barrett, The Honors College, Arizona State University, Tempe, AZ 85281, USA; 5 School of Sustainable Engineering and the Built Environment, Arizona State University, Tempe, AZ 85281, USA

**Keywords:** wastewater-based epidemiology, liquid biopsy, epigenetic, geography, incidence

## Abstract

Toxic chemicals and epigenetic biomarkers associated with cancer have been used successfully in clinical diagnostic screening of feces and urine from individuals, but they have been underutilized in a global setting. We analyzed peer-reviewed literature to achieve the following: (i) compile epigenetic biomarkers of disease, (ii) explore whether research locations are geographically aligned with disease hotspots, and (iii) determine the potential for tracking disease-associated epigenetic biomarkers. Studies (*n* = 1145) of epigenetic biomarkers (*n* = 146) in urine and feces from individuals have established notable diagnostic potential for detecting and tracking primarily gastric and urinary cancers. Panels with the highest sensitivity and specificity reported more than once were SEPT9 (78% and 93%, respectively) and the binary biomarker combinations GDF15, TMEFF2, and VIM (93% and 95%), NDRG4 and BMP3 (98% and 90%), and TWIST1 and NID2 (76% and 79%). Screening for epigenetic biomarkers has focused on biospecimens from the U.S., Europe, and East Asia, whereas data are limited in regions with similar/higher disease incidence rates (i.e., data for New Zealand, Japan, and Australia for colorectal cancer). The epigenetic markers discussed here may aid in the future monitoring of multiple cancers from individual- to population-level scales by leveraging the emerging science of wastewater-based epidemiology (WBE).

## 1. Introduction

Suitable targets for tracking non-communicable diseases such as cancer include human genes that are enhanced or repressed by epigenetic changes (e.g., DNA methylation; histone modifications, such as methylation or acetylation; and regulation by non-coding RNAs, such as miRNAs and long non-coding RNAs [1]) induced by exposure to harmful chemicals and disease progression. For instance, DNA hypomethylation has been observed in fetal serum of smoking mothers [2]. DNA methylation, an epigenetic modification where a methyl group is added to cytosine bases, was first discovered to silence genes nearly 50 years ago [3]. Global hypomethylation of DNA was a molecular change observed early in epigenetic disease research and is thought to be a general biomarker of environmental exposures linked to chronic diseases, such as cancer [2].

As epigenetic changes are known to be cell- or tissue-specific, current epigenetic research has largely focused on pure cell lines or cells extracted from tissues. However, disease-specific genetic and epigenetic changes are also carried by expelled DNA known as cell-free DNA (cfDNA) [4]. Meanwhile, less invasive diagnostic screening methods using liquid biopsies (e.g., of urine, feces, or blood) are gaining momentum in research and have shown promise in translation into clinical practice [5]. The cfDNA present in liquid biopsy samples represents a novel and promising target for researchers to identify the origin, mechanisms of release, and biomarkers associated with a given disease [6]. The extent of methylation of cfDNA extracted from liquid biopsies, specifically from urine, has been quantified successfully to inform on the presence and progression of a variety of cancers [5]. The potential utility for non-invasive biopsy samples such as urine and feces to diagnose cancer cannot be understated from a patient care perspective.

While epigenetics is primarily studied at the individual level, population epigenetics is an emerging scientific frontier. Population epigeneticists seek to follow epigenetic changes visible at the population level over time and space. Zhao et al. has recommended a framework to illustrate population characteristics of DNA methylation by comparing methylated and unmethylated DNA in a population of known size [7]. Population epigenetics may be used to seek epidemiological information by collecting samples from individuals and then finding average trends within and between populations.

Here, we review the data available on the analysis of non-invasive liquid biopsy samples (i.e., urine and feces) from around the world for the detection of epigenetic biomarkers associated with disease. The goal was to establish a knowledge platform informing future research on tracking the incidence of cancer and other illnesses at the population level. First, we analyzed and ranked literature-sourced biomarker panels for their diagnostic sensitivity and specificity. Second, we used a geographic information system (GIS) to map the geospatial origin and cohort size of urine and feces samples that have been previously analyzed for epigenetic biomarkers associated with disease outcomes. Third, we then compared these geographic locales with information reported on the case incidence rate of specific cancers.

## 2. Materials and Methods

A systematic literature review and geographic trend analyses were performed to determine epidemiological patterns.

### 2.1. Literature Sources

The Preferred Reporting Items for Systematic Reviews and Meta-Analyses (PRISMA) methodology was used to review search criteria. This systematic review was conducted in Scopus for all publications prior to December 2021 (Supplemental Figure S1). 

### 2.2. PRISMA Criteria

Keywords used to search for epigenetic biomarkers shed in urine or fecal liquid biopsy included “epigen* AND (fecal OR feces OR urine) AND cell AND biomarker”. Papers discussing biomarkers, e.g., urine-DNA methylation biomarkers, or cells, e.g., cell-based or classifier, were included in this analysis regardless of study sample size. Studies highlighting fecal transplants, blood plasma/serum liquid biopsy, chemical metabolite concentrations, or animal studies, e.g., rats and mice, were excluded.

### 2.3. Geographic Analysis

ArcGIS Pro 3.0.0 was used to analyze geospatial data. Qualitative and quantitative data reported by geographically linked studies were mapped to determine spatial trends. Cancer incidence rates were accessed from the Global Cancer Observatory database to compare study locations, sample size, and incidence rates at the country level.

### 2.4. Statistical Analysis

Biomarker accuracy was assessed using the combined frequency of sensitivity and specificity (log_10_ transformed). When converting these statistics using log_10_ transformation, threshold scores representing sensitivity and specificity can be added to a maximum of 2.0 for 100% accuracy. If threshold score calculation met or exceeded 1.5, then the gene or gene panel was considered viable as a diagnostic tool for the targeted disease. Sensitivity and specificity percentages for the same biomarker panel reported by more than one study were analyzed by calculating the average threshold scores, standard deviation, and subsequent 95% confidence interval.

## 3. Results

### 3.1. PRISMA Literature Review

Studies identified by the PRISMA search for epigenetic biomarkers in urine and feces have increased over time from 2003 to 2021 (Supplemental Figure S1). Of the 1145 studies screened, 146 articles were identified as relevant for qualitative and quantitative analysis (Supplemental Figure S2). Studies that analyzed epigenetic markers isolated from urine or feces for the diagnostic utility of disease were included in this review (Supplemental Table S1).

**Figure 1:**
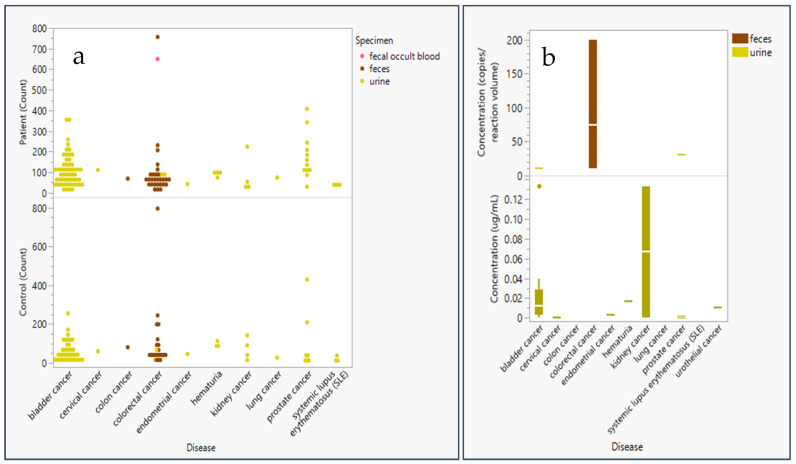
Sample size and concentration of genomic material used in epigenetic panel studies employing liquid biopsy materials. (**a**) Patient and control count (sample size) in epigenetic panel screening studies * are plotted as a function of specimen type: urine (yellow), feces (brown), and fecal occult blood (pink). (**b**) Genetic (DNA or RNA) concentration analyzed for epigenetic markers in screening studies ** plotted in a box and whisker plot (distributed around the mean) by disease and specimen type: urine (yellow) and feces (brown) by either concentration in copies/reaction volume (*n* = 7) or ug/mL (*n* = 24). * Did not include controls in a study of feces for colorectal cancer due to its large study size of 9167 controls [8] ** Excluded study with extremely high copies/reaction volume (1014 genome equivalent copies) for a study extracting genomic content from fecal matter in colorectal patients [9] and a study extracting high µg/mL volume (1 µg/mL) from urine in kidney cancer [10].

Most epigenetic biomarker studies of urine or fecal samples included 100 or fewer patients and fewer than 100 controls for sensitivity and specificity analysis of methylation panels (Figure 1). Additionally, Figure 1 indicates that the methylation status of DNA captured from feces is primarily used for colorectal (CRC) screening, while methylation levels in urine are mostly used to diagnose bladder cancer, prostate cancer, and kidney cancer. Average concentrations of genomic material analyzed in methylation assays were below 1 µg for RNA or DNA and below 5 µL for bisulfite-treated DNA. Extremely high concentrations were considered outliers and therefore excluded, as our analysis focused on the minimum concentrations used for successful methylation assays. Studies further shared concentrations averaging 49.29 genomic copies/reaction volume at a minimum of 10 copies/reaction volume and calculated concentrations averaging 0.02 µg/mL at a minimum of 0.0001 µg/mL (Figure 1).

### 3.2. Sensitivity and Specificity of Epigenetic Biomarkers Utilized in Clinical Screening of Urine and Feces

Studies reported statistics indicating the accuracy of the biomarkers chosen for their epigenetic panels. The percentage of tests in which a patient with the disease tested positive was recorded as sensitivity, whereas the percentage of tests in which a control subject tested negative was recorded as specificity. Studies with fewer false negatives are considered more accurate. If a study reports sensitivity + specificity above a threshold value of 1.5, further research or clinical translation are deemed warranted [11].

**Figure 2:**
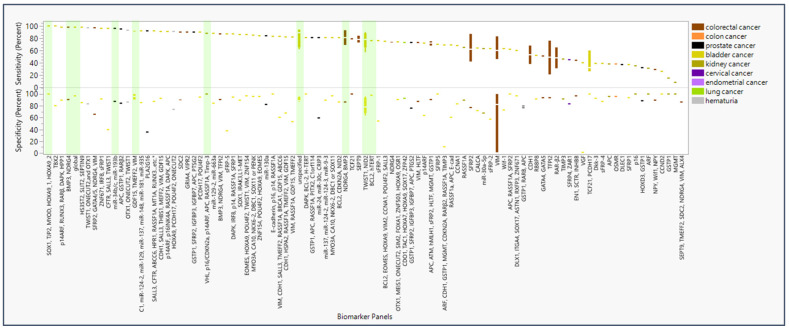
Sensitivity and specificity of panels of epigenetic biomarker detectable in urine and feces and diseases of diagnostic interest. Percent sensitivity and specificity reported in studies (*n* = 95) are color-coded by disease type (*n* = 9) that screened each epigenetic biomarker panel * in liquid biopsy of urine and feces. Bars highlighted in green represent biomarker panels and individual biomarkers with the highest sensitivity and specificity given cancer type or replicability. * Panel “SALL3, CFTR, ABCC6, HPR1, RASSF1A, MT1A, RUNX3 … etc.” also includes biomarkers ITGA4, BCL2, ALX4, MYOD1, DRM, CDH13, BMP3B, CCNA1, RPRM, MINT1, and BRCA1 in the full panel.

The panel with the highest sensitivity and specificity were SOX1, TJP2, MYOD, HOXA9_1, and HOXA9_2 [12], reporting at 100% for both sensitivity and specificity (Figure 2). The panels that were tested in more than one study with the highest sensitivity and specificity included the GDF15, TMEFF2, and VIM panel at 92.5% (95% CI: 96.7–88.3) and 95.0% (95% CI: 109.1–80.9), respectively [13,14]. In addition, the proprietary undisclosed (“unspecified”) panel for the product Bladder EpiCheck reported at 73.1% (95% CI: 101.1–45.2) and 87.4% (95% CI: 89.4–85.5), respectively [15,16,17]. The NDRG4 and BMP3 panel reported 95.2% (95% CI: 103.2–87.1) and 88.3% (95% CI: 93.1–83.5) [8,18]. A test with SEPT9 alone reported 78.2% (95% CI: 92.7–63.6) and 93.3% (95% CI: 96.7–89.9) [19,20]. Finally, the TWIST1 and NID2 panel reported 76.1% (95% CI: 101.1–51.1) and 79.4% (95% CI: 106.2–52.5) [21,22,23,24,25,26].

For biomarker panels informing on CRC, the highest sensitivity (98%) and specificity (90%) were found for BMP3 and NDRG4 (Table 1) [18]. For prostate cancer, the highest sensitivity (94.3%) and specificity (84.4%) were found, respectively, for miR-34b/c and miR-193b, monitored in separate individual assays [27]. For bladder cancer, a perfect score (100%) was found for both sensitivity and specificity in the SOX1, TJP2, MYOD, HOXA9_1, and HOXA9_2 panel [12]. For kidney cancer, a maximum sensitivity and specificity of 88% and 100%, respectively, were found for VHL, p16/CDKN2a, p14ARF, APC, RASSF1A, and Timp-3 [10]. However, only 10 panels have been studied more than once, many yielding sensitivity and specificity ranges below the optimal levels summarized here. Biomarkers with high sensitivity and specificity in multiple panels include RUNX3, SOX1, IRF8, and DAPK; these were used specifically for diagnosis of bladder cancer (Supplemental Figure S3). Biomarkers selected for multiple studies produced sensitivity and specificity results of greater variability and lower quality.

**Figure 3:**
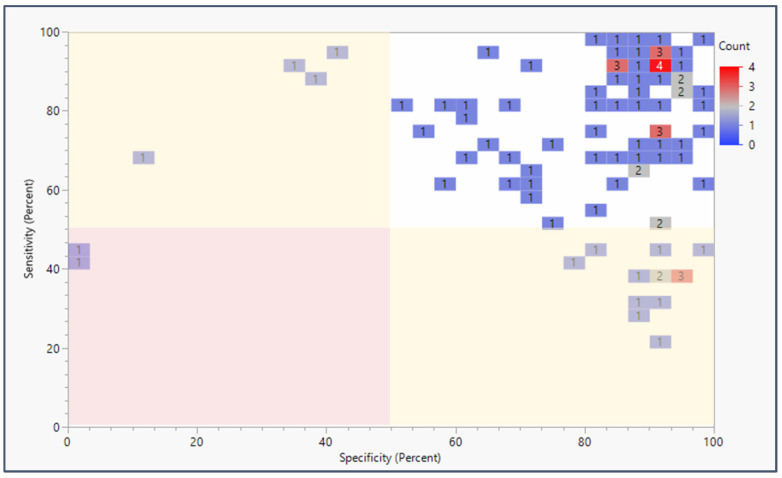
Sensitivity and specificity of epigenetic biomarker panels applied to liquid biopsy (urine and feces) by publication count. Percent sensitivity and specificity are plotted against each other in a heatmap to show publication counts for each sensitivity/specificity range reported by each epigenetic biomarker panel screening study. The red highlighted section represents sensitivity + specificity below the preferred threshold value of 1.5, the yellow sections highlight studies meeting the 1.5 threshold, and the green section shows the studies above the 1.5 threshold.

Nearly all studies of panels with epigenetic biomarker testing urine or feces from patients and controls reported over 80% values for both sensitivity and specificity (Figure 3). Eleven studies reported sensitivity and specificity above 90% for both. In fact, 73 of the 94 studies reported sensitivity and specificity values exceeding the threshold value of 1.5. The most common sensitivity + specificity score reported was approximately 1.8. When biomarkers are assessed individually, nearly all studies report over 60% for both panel sensitivity and panel specificity (Supplemental Figure S4). Biomarkers disaggregated from their panels were represented in 106 studies, reporting 80% or higher for both sensitivity and specificity. Nevertheless, any specific panel is only represented in four or fewer of the 94 studies that met the inclusion criteria of this literature review.

In addition, sensitivity and specificity reporting was compared to the sample (cohort) size of each study in Supplemental Figure S5. No significant patterns were identified, showing that studies with high sensitivity and specificity ranged significantly in sample size, from four-patient studies reporting 100% sensitivity and 80% specificity for TBX2 [28] to a 207-patient study reporting 98% sensitivity and 90% specificity for the BMP3 and NDRG4 panel [18].

### 3.3. Geographic Locations of Patient Cohorts Clinically Analyzed for Epigenetic Biomarkers in Urine and Feces Compared to Cancer Hotspots Globally

**Figure 4:**
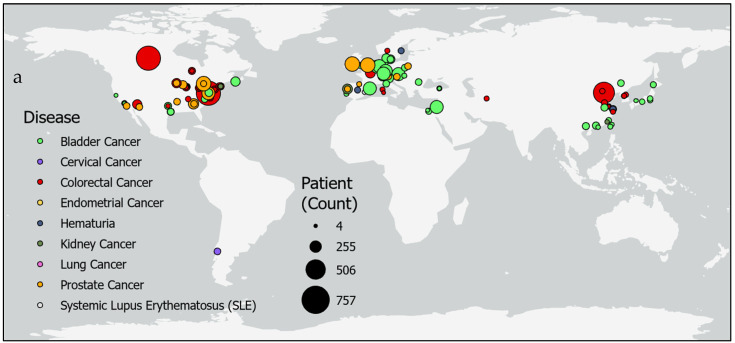

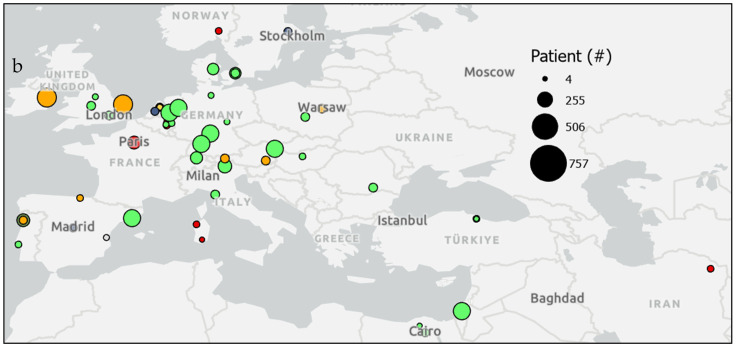
Urine or fecal epigenetic biomarker panel study patient counts by location. Maps show patient counts (sized circles) in epigenetic biomarker panel screening studies of various diseases (colors) (**a**) globally and (**b**) in regions in Europe and the Middle East.

Studies of epigenetic markers in human feces and urine thus far have been confined to regions of North America, Europe, and East Asia (Figure 4). Only four studies were reported from countries in the Middle East and North African regions, while populations in South Asia and Sub-Saharan Africa are not represented in this review due to a lack of search results from these regions. The largest studies were performed for colorectal cancer in Tianjin, China, (*n* = 650) [19] and in a shared study between Canada and the United States (*n* = 757 patients; *n* = 9167 controls) [8]. Additionally, studies for bladder cancer with the highest numbers of participants were in Vienna, Austria, (*n* = 357) [15] and a study combining Nijmegen and Hengelo, the Netherlands; Barcelona, Spain; Wurzberg and Sindelfingen, Germany; and Kfar, Israel (*n* = 353) [17]. Prostate cancer was also studied in large sample sizes in 18 clinics across the United States (*n* = 342 patients, *n* = 430 controls) [9] and in a combined study across Norwich, England; Toronto, Canada; and Dublin, Ireland (*n* = 408) [19]. However, other studies with relatively small sample sizes were reported for colorectal cancer in Hong Kong, China, (*n* = 20 patients, *n* = 30 controls) [76] and Cagliari, Italy, (*n* = 10) [70]. Likewise, studies for bladder cancer with small sample sizes were reported in Aachen, Germany (*n* = 20 patients; *n* = 5 controls) [55]; Maryland, United States (*n* = 20 patients; n = 20 controls) [64]; California, United States (*n* = 20 patients; *n* = 20 controls) [44]; and Rotterdam, the Netherlands (*n* = 4) [28].

In Supplemental Figure S6, incidence rates per 100,000 people are compared geographically for various types of cancer reported to the Global Cancer Observatory. While cancer incidence rates tend to be higher in males than females, regions with the highest rates are not always shared between the sexes. The highest incidence rates for colorectal cancer in males are reported in Canada, Japan, and Italy (70.3, 62.0, and 58.7, respectively), whereas colorectal cancer in females is highest in Canada, New Zealand, and Japan (41.9, 36.1, and 35.3). While incidence rates for bladder cancer top the charts in Italy, Spain, and France (48.5, 44,5, and 40) for males, the countries Chile, Malawi, and Canada report the highest bladder cancer rates (9.8, 9.2, and 9.1) in females. Kidney cancer in males is reportedly the highest in the Czech Republic, Lithuania, and Germany (22.1, 17.6, and 16.9), while females report their highest rates in Argentina, the Czech Republic, and the United States (10.4, 9.9, and 8.9). Lung cancer rates in males rank the highest in Turkey, the United States, and Poland (90.1, 84.9, and 67.9), whereas female rates are highest in the United States, Canada, and Denmark (53.6, 48.4, and 35.2). Meanwhile, prostate cancer rates are highest in Ecuador, Brazil, and Austria (177.8, 157.4, and 140.4). Finally, cervical cancer rates are highest in Zimbabwe, Malawi, and China (86.7, 76.3, and 71.8).

**Figure 5:**
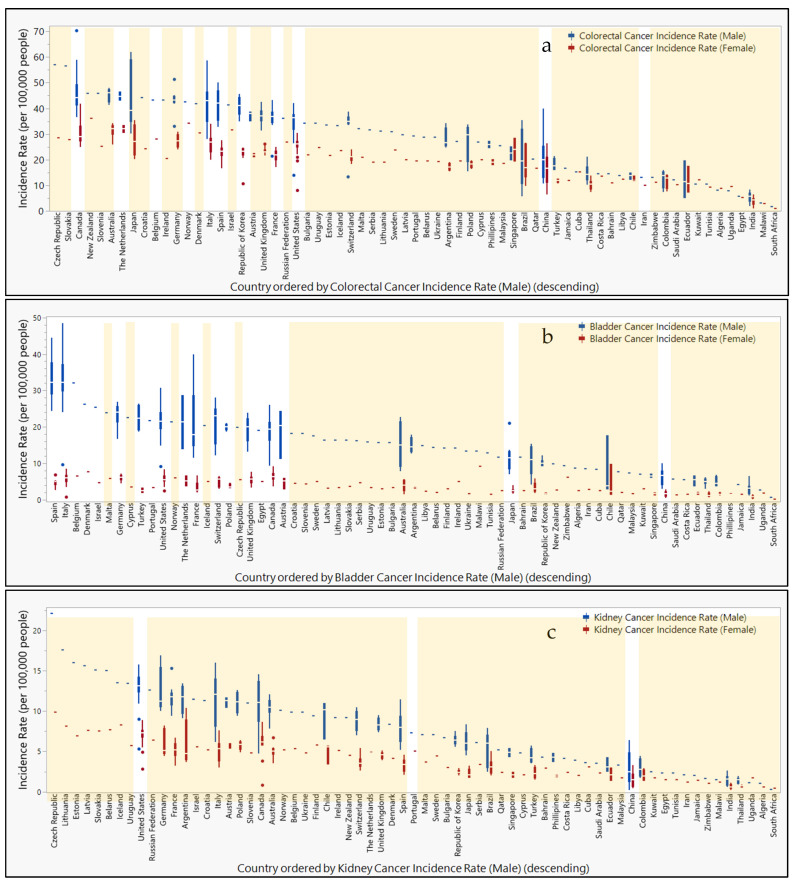

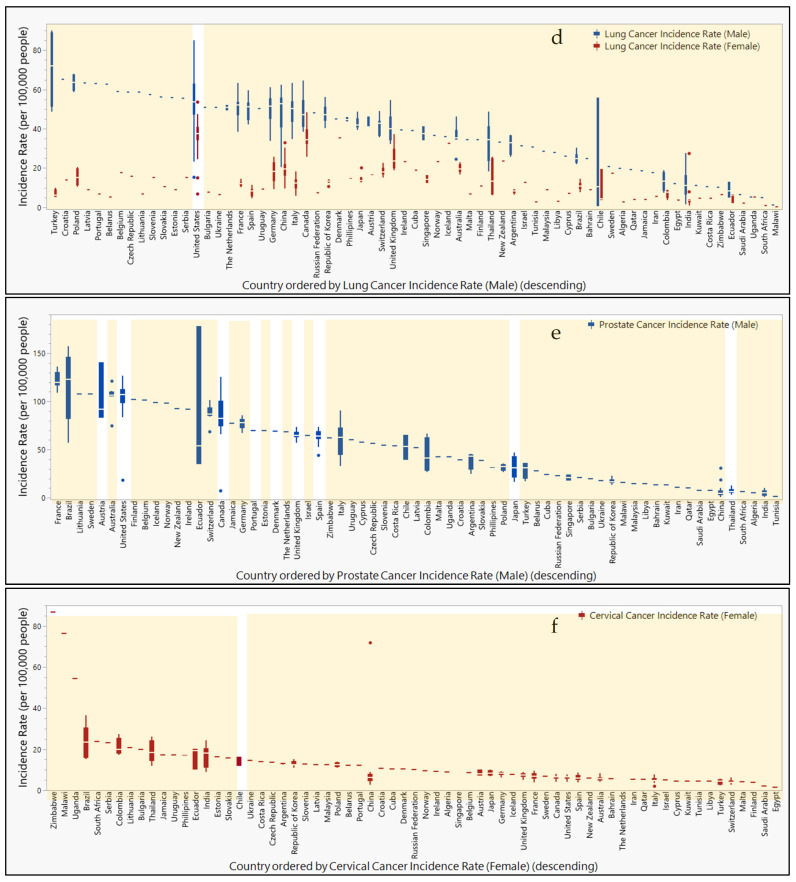
Countries ranked by cancer incidence compared to urine or fecal epigenetic biomarker panel study locations. Box and whisker plots ranked in descending order by cancer incidence rates (per 100,000 people) for (**a**) colorectal, (**b**) bladder, (**c**), kidney, (**d**) lung, (**e**) prostate, and (**f**) cervical cancers. Male (blue) and female (red) incidence rates are separated and color-coded. Orange highlight bars indicate countries that are lacking representation in urine or fecal epigenetic biomarker panel studies for each disease. Countries not highlighted are represented in urine or fecal epigenetic biomarker panel studies identified in this review.

When comparing study locations of epigenetic biomarkers isolated from urine and feces, Figure 5 shows the misalignment of the study location with regions reporting high incidence rates of specific cancers. For colorectal cancer, Japan, the Czech Republic, Slovakia, and Germany are notably absent from the study locations. Bladder cancer studies do not include participants from Malawi or Chile. While studies have been conducted within the United States for kidney and lung cancers, other countries with top ranking incidence rates for kidney cancer that are not represented in these studies include the Czech Republic, Lithuania, Germany, and Argentina. Similarly, countries with high incidence rates for lung cancer without participation in these studies include Turkey, Poland, Canada, and Denmark. For prostate cancer, a study in Austria reported findings, but other countries with high incidence rates were not represented (e.g., Ecuador and Brazil). Meanwhile, the top three countries reporting the highest incidence rates for cervical cancer did not include participants in these studies (i.e., Zimbabwe, Malawi, and China).

## 4. Discussion

This review aimed to assess epigenetic marks found in non-invasive matrices (e.g., urine or feces) as potentially reliable biomarkers of disease. Prior research has shown a relationship between global hypermethylation and the likelihood of receiving a cancer diagnosis [100]. More recently, methylation status of cfDNA in urine has been associated with the prevalence and development of cancer. This paper identified several epigenetic biomarkers and biomarker panels that achieved a sensitivity and specificity above the calculated accuracy threshold (1.5) for several cancers, particularly gastric and urinary cancers. Meanwhile, a global geographic analysis of cancer incidence indicates that many locations with high or the highest incidence of disease are currently underrepresented in studies utilizing epigenetic biomarker panels for disease screening.

### 4.1. Performance of Epigenetic Biomarker Panels

While it is unlikely that the epigenetic status of cfDNA is identical to DNA within the cells of a tumor or tissue of origin, sensitivity and specificity tests indicate high correlations (Figure 4) between cfDNA methylation profiles and the presence of disease validated from traditional diagnosis methods. Not only have DNA fragmentation sizes and end alterations been associated with cancer pathologies [5], but the results of this review show a significant relationship between cfDNA methylation levels and specific diseases.

The recent literature showed that epigenetic panels tested on urine or feces performed with similarly wide ranges of sensitivity and specificity as panels tested on other liquid biopsy (i.e., plasma) [101,102,103,104,105]. Sensitivity ranged from 60% to 92%, and specificity ranged from 55% to 100% for screening a variety of cancers (i.e., kidney cancer, prostate cancer, and CRC) [101,102,103,104,105]. Meanwhile, more invasive biopsies have shown a higher sensitivity and specificity. For instance, the sensitivity and specificity of the same epigenetic panel to diagnose CRC was notably lower in plasma (81.1% and 96.9%, respectively) compared to tissue (93.8% and 99.9%) [104]. Nevertheless, the authors recognize the importance of testing the utility of the least invasive samples for the benefit of the patient.

The highest values for sensitivity and specificity of epigenetic biomarker panels were obtained from studies which differed markedly in sample size (Supplemental Figure S5). While it should be anticipated that the likelihood of false positive or false negative results would be higher in studies with more subjects, doubts emerge as to the scalability of results reported in studies featuring small sample sizes.

More studies have been undertaken with more promising (more sensitive and selective) biomarkers, whereas 22% of studies focused on biomarkers that were found to be underperforming. Moreover, Figure 2 and Figure 3 reveal little to no standardization in the selection and testing of epigenetic biomarkers in urinary or gastric screening panel studies. The large number of assays and small number of replicating studies suggest the need to focus research efforts to coalesce on utilizing biomarker panels of both high sensitivity and high specificity.

This literature search revealed a lack of convergence toward the unified use of selected biomarkers across multiple studies, suggesting that the use of epigenetic biomarkers of disease in clinical studies relying on non-invasive matrices is still in the early stages of identifying biomarkers’ import to public health. Expanding testing and eventual monitoring to include disease-specific biomarkers in regions with high incidence of disease (the United States, Canada, Japan, and the Czech Republic) presents opportunities for identifying susceptible populations around the world (Supplemental Figure S6). Nevertheless, the selection of regions to be studied needs to be informed both by male and female incidence rates. The sensitivity and specificity of biomarkers for identifying cancer in males should include countries such Italy (colorectal cancer and bladder cancer), Spain and France (bladder cancer), Lithuania and Germany (kidney cancer), Turkey and Poland (lung cancer), as well as Ecuador, Brazil, and Austria (prostate cancer), while studies for identifying cancer in females should be held in New Zealand (colorectal cancer), Chile (bladder cancer), Malawi (bladder cancer and cervical cancer), Argentina (kidney cancer), Denmark (lung cancer), as well as Zimbabwe and China (cervical cancer). The literature revealed that participants from Japan would benefit from studies of colorectal cancer in both males and females (Figure 5). Additionally, studies for bladder cancer in females are absent from the highest-incidence countries, Malawi and Chile. Furthermore, regional selection of participants needs to be informed by the cancers with the highest incidence rates. A study in Chile for cervical cancer could have also included biomarkers for bladder cancer as well to address the high incidence rates of this cancer locally. Additionally, while bladder cancer studies have been performed in several cities in Japan, the high incidence of colorectal cancer in the country suggest studies should also include biomarkers specific to CRC identification. Meanwhile, studies across China for a variety of cancers including colorectal, bladder, kidney, and prostate could have also included biomarkers for cervical cancer to address these extreme rates in females.

To assess the potential individual value of any one biomarker present in multiple panels, panels were disaggregated to allow for each biomarker to be examined individually (Supplemental Figure S3). Various biomarker panels overlapped with others; however, the contribution of each individual marker in the panel could not be evaluated due to a lack of data. Furthermore, the task of individually evaluating each biomarker contained in a biomarker panel was made impossible by their proprietary (i.e., undisclosed) identity.

### 4.2. Geographic Considerations for Epigenetic Biomarker Panels

Studies of epigenetic markers for the screening of urinary and gastric cancers are scattered in several regions across the world (Figure 4). However, nearly the entire southern hemisphere is lacking studies on epigenetic biomarkers informing the screening for gastric and urinary cancer. Since variation in methylation status in subjects studied across the East Asian countries Taiwan, Hong Kong, and China [43], as well as globally between Siberia, Cambodia, Pakistan, Algeria, and Mexico [106], suggested that geographic location could impact the success of using epigenetic biomarkers as a diagnostic tool, it is necessary to enroll patients and controls in regions not currently represented in the literature. Furthermore, incidence rates reported to the Global Cancer Observatory reveal populations at high risk for cancer in regions not participating in epigenetic biomarker screening studies of urine or feces (Figure 5).

To address the likely heterogeneity in biomarker performance across diverse populations, particularly in geographic regions with relatively few resources, researchers in more affluent nations would improve the efficiency and efficacy of biomarker identification by collaborating with clinics in various regions globally. While several studies included patients from multiple states within the same country or in nearby countries, it was rare to find studies reaching patients across global classifications. Future studies should plan to intentionally recruit patients from countries with high incidence rates of the target cancer, particularly if populations within these countries have traditionally reported low participation in epigenetic panel studies. Of course, funding agencies aiming to reduce global health disparities should also encourage experimental designs incorporating more diverse populations and scopes to include the target cancer type to others of high need in particular countries. Providing funding opportunity announcements (FOAs) targeting epigenetic panel studies for kidney cancer in the Czech Republic, Lithuania, Estonia, Latvia, Slovakia, and Belarus, for lung cancer in Turkey and Croatia, or for cervical cancer in Zimbabwe, Malawi, and Uganda would further encourage research to meet the needs of populations with the highest incidence of these cancers. If research in these regions is not conducted, then the selection of biomarkers for diseases of particular importance to these populations (e.g., high incidence rates, as shown in Figure 5) may not be as effective during screening, leading to reduced diagnosis and treatment of susceptible individuals.

### 4.3. Translation to Clinic

Several considerations impact the likelihood of a screening test making it to the clinic. For instance, the diagnostic value of a test that is less invasive to the patient needs to be weighed against a more invasive test. A recent systematic review of epigenetic panel studies for CRC compared tissue, plasma, feces, and urine specimens [107]. After calculating threshold scores for each panel, six tests for five single-gene panels (NDRG4 (1.7), SEPT9 (1.9), SFRP2 (1.9; 95% CI: 1.8–1.9), SPG20 (1.9), and TFPI2 (1.9)) were successful when cfDNA was extracted from tissue. Similarly, for fecal specimens, five tests for four genes (ITGA4 (1.8), SFRP2 (1.8; 95% CI: 1.7–1.8), SPG20 (1.8), and TFPI2 (1.8)) were successful. Three tests with cfDNA from plasma for three genes (SEPT9 (1.8), SFRP1 (1.7), and SPG20 (1.8)) were successful. Only one test for one gene (VIM (1.7)) was reported with diagnostic value for CRC. When comparing specimen type, cfDNA extracted from tissue performed slightly better than non-invasive specimens given the relatively higher threshold scores for SPG20; however, cfDNA extracted from fecal and plasma performed comparably. As for panels with multiple biomarkers, two panels reported high threshold scores for tissue (Wif-1 (1.7); RARB2, p16INK4a, MGMT, and APC (1.8)). Two panels were successful for feces (BMP3, NDRG4, VIM, TFPI2, mutant KRAS, B-actin, and Hb (1.8); RARB2, p16INK4a, MGMT, and APC (1.6)), while only one panel reported high sensitivity and specificity for plasma (APC, MGMT, RASSF2A, and Wif-1 (1.8)). The RARB2, p16INK4a, MGMT, and APC panel’s performance was better for the tissue than fecal test, but one could argue that the difference in sensitivity may be acceptable when considering the invasive procedure needed to extract cfDNA from tissue compared to feces.

The possibility of cfDNA degrading in specimens prior to analysis is another concern clinicians need to consider. Storage conditions, such as access to a −80 °C freezer, can prevent further fragmentation and cell lysis [108]. Urine poses a particular challenge due to its high degradation rate when not stabilized with buffers to reduce nuclease activity [109]. Therefore, the development of standards for the development and application of biomarker panels as diagnostic tools will need to include strict storage and sample preparation guidance [110]. Ultimately, the authors acknowledge cfDNA presents limitations and uncertainties (e.g., indirect correlations to disease and degradation over time and in complex matrices) not present in other sample media, such as tissue, where cellular DNA profiles may be analyzed at the source and within the general protection of the nucleus. Nevertheless, cfDNA offers clinicians the opportunity to collect less invasive samples for equally valuable diagnostic potential while inflicting less harm on the patient.

Furthermore, the benefits of translating a viable biomarker into a clinical tool need to outweigh the costs, which can be significant. While many biomarkers have been shown to be viable for gastric and urinary cancers such as bladder and CRC, one study also reported a successful DNA methylation analysis (1.7) of a panel (CDO1, TAC1, HOXA7, HOXA9, SOX17, and ZFP42 promoters) to diagnose lung cancer [20]. Therefore, cfDNA from urine and feces could offer diagnostic value to other highly prevalent cancers. Often, for-profit companies lead the charge in moving a biomarker to clinical practice due to the incentive to sell a product for consumption [110]. Researchers, however, are almost exclusively incentivized to publish independent papers as often as possible. For-profit companies may not consider the viability of a particular biomarker over another, while researchers may not consider methods of detecting biomarkers with clinical infrastructure limitations. Regulatory changes at the national and international levels requiring these two spheres to collaborate rather than simply coexisting could significantly improve the potential for promising biomarkers to make it to patients.

As of this literature search, the U.S. Food and Drug Administration (FDA) has approved several epigenetic screening tests for CRC. The first fecal-based test using epigenetic markers, Cologuard, was approved by the FDA in 2014. However, it was recommended to perform additional screening in clinic [111]. In 2016, the FDA approved the blood-based epigenetic test trademarked Epi proColon as an alternative test to colonoscopy or stool-based fecal immunochemical testing [112]. Therefore, it is reasonable to assert high potential for the FDA to approve a fecal- or urine-based test using epigenetic markers as an alternative to the current more invasive clinical tests upon high sensitivity and specificity reporting in multiple trials.

### 4.4. Applications of Population Epigenetics for Public Health

Epigenetic marks that are highly correlated to patients with disease compared to controls have the potential to be observed at the population level. If these marks can be observed from cfDNA in urine or feces, then composited samples in wastewater may offer an efficient, effective, and timely media to observe varying concentrations of epigenetic marks between populations. Wastewater-based epidemiology (WBE) has recently become popularized for its utility in predicting surges in infectious disease rates during the height of the COVID-19 pandemic [113,114]. Since the methodology to detect SARS-CoV-2 requires comparable technology for genomics analysis (e.g., RT-qPCR, Illumina sequencing) and epigenetic marker analysis, it stands to reason that WBE pipelines could be adapted to detect epigenetic marks. If a WBE pipeline was developed for epigenetic marks associated with cancers in this review, populations could be monitored and subsequently supported with targeted clinical screening, treatment, and other healthcare resources.

### 4.5. Potential Confounding Results

Biomarkers that are not specific to disease represent a challenge for public health interventions. Epigenetic changes may be indicative of one or more diseases, such as global hypermethylation or hypomethylation. Biomarkers that are associated with only one disease would be better suited for monitoring each disease of interest. It is therefore imperative to select genetic biomarkers that are specific to disease.

Additionally, studies with high sensitivity and specificity results may not be viable due to the potential for a Type 1 error (Figure 1 and Figure 4). A study for colorectal cancer with a sample size of *n* = 10 [76] and another for bladder cancer with a sample size of only *n* = 4 for a particular region [44] gives the reviewers pause when considering recommendations of biomarkers. Increasing sample sizes would increase confidence in results prior to selection for further study.

Due to the challenges mentioned above and other aspects, some markers that rank highly (Figure 2) ultimately may not be as well suited compared to others for future clinical application. To be of utility, biomarkers tracked by epigenetic panel testing must occur at appreciable concentrations. For a marker to be a reliable indicator of public health, it would ideally be both abundant, i.e., occurring at high concentrations, and highly modulated in concentration as a function of disease status to produce an epigenetic signal that is both detectable and quantitatively informative. If biomarker panel(s) are widely implemented at the clinical setting for diagnostic purposes, epidemiologists tracking disease rates at the population level (e.g., using WBE methodology) will rely on the knowledge that the chosen biomarkers are highly sensitive, specific, and quantifiable. WBE methodology analyzing wastewater samples compositing urine and feces from a significant number of individuals is more likely to detect signals of epigenetic mark differences in regions with high prevalence of disease. The choice to use WBE for quantification of epigenetic marks at the population level would therefore hinge on whether a high intensity signal with a high dynamic range could be expected given a region’s incidence and prevalence of disease.

### 4.6. Literature Search Window Extension

The authors would also like to note that funding was made available to support this literature review through the year 2021. Since the preparation of the present manuscript, several primary research articles have been published utilizing urine or feces as a diagnostic matrix for epigenetic marker testing, particularly regarding screening for bladder cancer. A recent study evaluating BladMetrix in Norwegian patients, a proprietary epigenetic test for bladder cancer using urine, reported a sensitivity of 96% and a sensitivity of 95% in a sample size of 112 individuals [115]. Another study in China found that a genome-wide DNA methylation profile test for bladder cancer was 100% sensitive to high-grade bladder cancer and 62% sensitive to low-grade bladder cancer, both with 100% specificity [116]. Hypermethylation of individual biomarkers TWIST1, hTERT, NID2, and VIM was detected with a sensitivity of 92%, 97%, 84%, and 83%, respectively, and a specificity of 100% for each using urine sediment samples in Moroccan bladder cancer patients [117]. The performance of a panel of ZNF671, OTX1, and IRF8 developed with a decision tree method attained a sensitivity of 75% and a specificity of 91% in Taiwanese patients [118]. A final study published this year for the detection of CRC in Iranian patients found a sensitivity of 52% and a specificity of 100% using a test for the methylation status of the CDX1 gene in fecal samples [119]. The new study representing bladder cancer patients in Norway is a particularly valuable addition to the literature analyzed in the prior pool collected prior to 2022, as Norway was not previously represented and yet reports a higher incidence of bladder cancer relative to most other nations globally. Nevertheless, the addition of these studies does not change the performance results shared in this literature review, as the best panels remain for both bladder cancer (with 100% sensitivity and specificity for the SOX1, TJP2, MYOD, HOXA9_1, HOXA9_2 panel [12]) and CRC (with a sensitivity of 98% and a specificity of 90% for the BMP3 and NDRG4 panel [18]). We sincerely hope future research continues to narrow biomarker panels by scrutinizing performance for the screening of bladder cancer, CRC, and other diseases.

## 5. Conclusions

Methylated genes detectable in urine and feces of individuals and human populations have the potential to enhance the early diagnosis of a variety of cancers. Development and broad-scale adoption of epigenetic diagnostic panels may enhance both clinical screening of individuals. Researchers offering expertise or potentially partnering with health officials to decide on the best means to use these methods in systematic resource allocation would open a collaboration to assist in the early detection of disease in local communities. Tracing epigenetic marks in composited urine and feces of large populations could potentially inform healthcare professionals regionally as to where and when to mobilize clinical tools to aid in the diagnosis and treatment of difficult-to-detect major diseases.

The possibility exists of improving health outcomes of cancer with a diagnostic strategy that proved helpful during the COVID-19 pandemic: the population-wide monitoring for threat agents and disease biomarkers in composited urine and stool from human communities, followed by a targeted deployment of clinical interventions to reduce morbidity and mortality. This scenario appears plausible given the large spectrum of epigenetic biomarkers identified in this study. However, there are many significant limitations to utilize these diagnostics most effectively. Whether a transition is possible from monitoring individuals only to surveilling whole populations via WBE for cancer will depend on many factors, including the following: the quantity of biomarkers excreted, the stability of epigenetic markers in wastewater, the dynamic range of marker expression, the ratio of expression in controls versus the diseased, etc. Epigenetic testing applied from the individual to the population level could improve the quality of life of both patients and their caregivers while reducing healthcare costs. Exploring this possibility will require time and further studies, however, as the science of population epigenetics and population diagnostics is still in its infancy.

## Figures and Tables

**Table 1:** Ranked biomarker panels by disease, sensitivity, and specificity.

Disease	Liquid Biopsy Specimen	Biomarker Panel	Epigenetic Marker Type	Sensitivity (Disease as Positive)	Specificity (Control as Negative)	Threshold Score *	Source
bladder cancer	urine	hyper: SOX1, TJP2, MYOD, HOXA9_1, HOXA9_2 hypo: VAMP8, CASP8, SPP1, IFNG, CAPG, HLADPA1, RIPK3	methylation	100.0	100.0	2.0	[12]
bladder cancer	urine	TBX2	methylation	100.0	80.0	1.8	[28]
bladder cancer	urine	p14ARF, RUNX3, RARβ, DAPK, and HPP1	methylation	98.2	88.9	1.9	[29]
bladder cancer	urine	150 CpG loci	methylation	98.0	97.0	2.0	[30]
bladder cancer	urine	HS3ST2, SLIT2 and SEPTIN9	methylation	97.6	84.8	1.8	[31]
bladder cancer	urine	ZNF671, IRF8 and sFRP1	methylation	96.2	90.9	1.9	[32]
bladder cancer	urine	CFTR, SALL3, and TWIST1	methylation	96.0	40.0	1.4	[33]
bladder cancer	urine	GDF15, TMEFF2, and VIM CpG island-containing genes	methylation	94.0	90.0	1.8	[13]
bladder cancer	urine	cfDNA	methylation	93.5	95.8	1.9	[34]
bladder cancer	urine	3 BC-specific genes2 internal control genes	methylation	93.5	92.6	1.9	[35]
bladder cancer	urine	CADM1, FAM19A4, GHSR, MAL, PHACTR3, PRDM14, SST, ZIC1, miR-124-2, miR-129, miR-137, miR-148, miR-181 and miR-935	methylation	92.0	85.0	1.8	[36]
bladder cancer	urine	SALL3, CFTR, ABCC6, HPR1, RASSF1A, MT1A, RUNX3, ITGA4, BCL2, ALX4, MYOD1, DRM, CDH13, BMP3B, CCNA1, RPRM, MINT1, and BRCA1	methylation	91.7	87.0	1.8	[37]
bladder cancer	urine	GDF15, TMEFF2 and VIM promoter	methylation	91.0	100.0	1.9	[14]
bladder cancer	urine	CDH1, SALL3, THBS1, MEFF2, VIM, and GDF15: 0.89, and with cytology	methylation	91.0	92.0	1.8	[38]
bladder cancer	urine	p14ARF, p16INK4A, RASSF1A, DAPK, and APC promoters	methylation	91.0		0.9	[39]
bladder cancer	urine	PCDH17 and POU4F2	methylation	90.0	94.0	1.8	[40]
bladder cancer	urine	TWIST1 and NID2	methylation	90.0	93.0	1.8	[22]
bladder cancer	urine	15 (proprietary)	methylation	88.9	88.0	1.8	[15]
bladder cancer	urine	miR-129-2 and miR-663a promoters	methylation	87.7	84.0	1.7	[41]
bladder cancer	urine	TWIST1 and NID2	methylation	87.5	95.8	1.8	[23]
bladder cancer	urine	sFRP-5	methylation	87.0	37.0	1.2	[42]
bladder cancer	urine	DAPK, IRF8, p14, RASSF1A and SFRP1	methylation	86.7	94.7	1.8	[43]
bladder cancer	urine	SOX1, IRAK3, andL1-MET	methylation	86.0	89.0	1.8	[44]
bladder cancer	urine	EOMES, HOXA9, POU4F2, TWIST1, VIM, and ZNF154	methylation	85.5	97.0	1.8	[45]
bladder cancer	urine	MYO3A, CA10, NKX6-2, DBC1, and SOX11 or PENK	methylation	85.0	95.0	1.8	[46]
bladder cancer	urine	ZNF154, POU4F2, HOXA9, and EOMES	methylation	84.0	96.0	1.8	[47]
bladder cancer	urine	E-cadherin, p16, p14, and RASSF1A	methylation	83.0	100.0	1.8	[48]
bladder cancer	urine	VIM, CDH1, SALL3, TMEFF2, RASSF1A, BRCA1, GDF15, and ABCC6	methylation	83.0	60.0	1.4	[38]
bladder cancer	urine	CDH1, HSPA2, RASSF1A, TMEFF2, VIM, and GDF15	methylation	82.0	68.0	1.5	[38]
bladder cancer	urine	VIM, RASSF1A, GDF15, and TMEFF2	methylation	82.0	53.0	1.4	[38]
bladder cancer	urine	DAPK, BCL-2, and H-TERT	methylation	81.1	100.0	1.8	[49]
bladder cancer	urine	MYO3A, CA10, NKX6-2, and DBC1 or SOX11	methylation	81.0	97.0	1.8	[46]
bladder cancer	urine	miR-137, miR-124-2, miR-124-3, and miR-9-3	methylation	81.0	89.0	1.7	[50]
bladder cancer	urine	BCL2, CDKN2A and NID2	methylation	80.9	86.4	1.7	[51]
bladder cancer	urine	TWIST1 and NID2	methylation	79.0	63.0	1.4	[21]
bladder cancer	urine	TWIST1 and NID2	methylation	76.2	83.3	1.6	[26]
bladder cancer	urine	BCL2 and hTERT promoters	methylation	76.0	98.0	1.7	[52]
bladder cancer	urine	sFRP-1	methylation	75.9	53.7	1.3	[42]
bladder cancer	urine	BCL2, EOMES, HOXA9, VIM2, CCNA1, POU4F2, and SALL3	methylation	75.0		0.8	[53]
bladder cancer	urine	OTX1, MEIS1, ONECUT2, SIM2, FOXA1, ZNF503, HOXA9, and OSR1	methylation	74.0	90.0	1.6	[28]
bladder cancer	urine	p14ARF promoter	methylation	72.1	63.6	1.4	[54]
bladder cancer	urine	SFRP5	methylation	70.0	100.0	1.7	[55]
bladder cancer	urine	RASSF1a, APC and E-cad promoters	methylation	69.0	60.0	1.3	[56]
bladder cancer	urine	APC, ARF, CDH1, GSTP1, MGMT, CDKN2A, RARβ2, RASSF1A, and TIMP3 promoters	methylation	69.0	10.0	0.8	[57]
bladder cancer	urine	15 (proprietary)	methylation	68.2	88.0	1.6	[17]
bladder cancer	urine	CCNA1 promoter	methylation	68.0	83.0	1.5	[58]
bladder cancer	urine	CDH1 promoter	methylation	67.4	93.9	1.6	[54]
bladder cancer	urine	TWIST1 and NID2	methylation	67.0	69.0	1.4	[24]
bladder cancer	urine	RAR-β2	methylation	65.0	89.7	1.5	[59]
bladder cancer	urine	CALCA promoter	methylation	63.5	71.5	1.4	[58]
bladder cancer	urine	Wif-1	methylation	63.0	72.2	1.4	[42]
bladder cancer	urine	sFRP-2	methylation	63.0	57.4	1.2	[42]
bladder cancer	urine	15 (proprietary)	methylation	62.3	86.3	1.5	[16]
bladder cancer	urine	APC, RASSF1A and SFRP2	methylation	62.0	100.0	1.6	[60]
bladder cancer	urine	TCF21 and PCDH17 promoters	methylation	60.0	100.0	1.6	[61]
bladder cancer	urine	DLX1, ITGA4, SOX17, ASTN1, RXFP3, and ZNF671	methylation	60.0	96.7	1.6	[62]
bladder cancer	urine	TWIST1 and NID2	methylation	57.0	72.0	1.3	[25]
bladder cancer	urine	RBBP8	methylation	52.0	91.0	1.4	[63]
bladder cancer	urine	VGF	methylation	40.0	1.0	0.4	[64]
bladder cancer	urine	Dkk-3	methylation	38.9	92.6	1.3	[42]
bladder cancer	urine	sFRP-4	methylation	38.9	87.0	1.3	[42]
bladder cancer	urine	SFRP1	methylation	36.7	93.3	1.3	[65]
bladder cancer	urine	TCF21 and PCDH17 promoters	methylation	32.0	100.0	1.3	[61]
bladder cancer	urine	CCND2 promoter	methylation	26.0	100.0	1.3	[58]
bladder cancer	urine	TCF21 and PCDH17 promoters	methylation	26.0	100.0	1.3	[61]
cervical cancer	urine	SFRP4 and ZAR1 promoters	methylation	45.2	83.3	1.3	[66]
colorectal cancer	feces	BMP3 and NDRG4	methylation	98.0	90.0	1.9	[18]
colorectal cancer	feces	SFRP2, GATA4/5, NDRG4 and VIM	methylation	96.4	65.0	1.6	[67]
colorectal cancer	feces	BMP3 and NDRG4	methylation	92.3	86.6	1.8	[8]
colorectal cancer	feces	SDC2	methylation	90.2	90.2	1.8	[68]
colorectal cancer	feces	SDC2	methylation	90.0	90.9	1.8	[69]
colorectal cancer	feces	GRIA4 and VIPR2	methylation	90.0		0.9	[70]
colorectal cancer	feces	BMP3, NDRG4, vimentin, and TFPI2 plus mutant KRAS	methylation	87.0	90.0	1.8	[71]
colorectal cancer	feces	SFRP2 promoter	methylation	87.0		0.9	[72]
colorectal cancer	feces	SEPT9	methylation	83.3	92.1	1.8	[20]
colorectal cancer	feces	VIM	methylation	83.0	82.0	1.7	[73]
colorectal cancer	feces	NDRG4	methylation	76.2		0.8	[74]
colorectal cancer	feces	TFPI2	methylation	76.0	93.0	1.7	[75]
colorectal cancer	feces	APC, ATM, hMLH1, sFRP2, HLTF, MGMT, and GSTP1	methylation	75.0	90.0	1.7	[76]
colorectal cancer	fecal occult blood	SEPT9	methylation	73.0	94.5	1.7	[19]
colorectal cancer	urine	NDRG4	methylation	72.6		0.7	[74]
colorectal cancer	feces	VIM and HLTF	methylation	72.5	86.9	1.6	[77]
colorectal cancer	feces	BMP3 and NDRG4	methylation	69.2	86.6	1.6	[8]
colorectal cancer	feces	APC, ATM, hMLH1, sFRP2, HLTF, MGMT, and GSTP1	methylation	68.0	90.0	1.6	[76]
colorectal cancer	feces	SFRP2 promoter	methylation	61.8		0.6	[72]
colorectal cancer	feces	VIM	methylation	60.0	100.0	1.6	[78]
colorectal cancer	feces	GATA4 and GATA5 promoters	methylation	51.0	93.0	1.4	[79]
colorectal cancer	feces	Vimentin exon 1	methylation	46.0	90.0	1.4	[80]
colorectal cancer	feces	EN1, SCTR, INHBB CpG Islands	methylation	44.0	97.0	1.4	[81]
colorectal cancer	feces	SFRP2 promoter	methylation	42.3	76.8	1.2	[72]
colorectal cancer	feces	OSMR CpGs	methylation	38.0	95.0	1.3	[82]
colorectal cancer	feces	NPY, Wif1, and NPY	methylation	29.2	89.7	1.2	[83]
colorectal cancer	feces	TFPI2	methylation	21.0	93.0	1.1	[75]
colorectal cancer	urine	SEPT9, TMEFF2, SDC2, NDRG4, VIM and ALX4	methylation		86.0	0.9	[84]
hematuria	urine	TWIST1, ONECUT2, and OTX1	methylation	97.0	83.0	1.8	[85]
hematuria	urine	OTX1, ONECUT2 and TWIST1	methylation	93.0	86.0	1.8	[86]
hematuria	urine	HOXA9, PCDH17, POU4F2, and ONECUT2	methylation	90.5	73.2	1.6	[87]
kidney cancer	urine	VHL, p16/CDKN2a, p14ARF, APC, RASSF1A, and Timp-3	methylation	88.0	100.0	1.9	[10]
kidney cancer	urine	TCF21	methylation	79.0	100.0	1.8	[88]
kidney cancer	urine	RASSF1A promoter	methylation	65.0	89.0	1.5	[89]
kidney cancer	urine	microRNA-30a-5p: two CpGs	methylation	63.0	67.0	1.3	[90]
kidney cancer	urine	TIMP3 promoter	methylation	46.0	91.0	1.4	[89]
kidney cancer	urine	APC promoter	methylation	38.0	96.0	1.3	[89]
kidney cancer	urine	CDH1 promoter	methylation	38.0	95.0	1.3	[89]
kidney cancer	urine	p16 promoter	methylation	35.0	100.0	1.4	[89]
kidney cancer	urine	ARF promoter	methylation	31.0	100.0	1.3	[89]
kidney cancer	urine	RAR-β2 promoter	methylation	31.0	91.0	1.2	[89]
kidney cancer	urine	GSTP1 promoter	methylation	15.0	100.0	1.2	[89]
kidney cancer	urine	MGMT promoter	methylation	8.0	100.0	1.1	[89]
lung cancer	urine	CDO1, TAC1, HOXA7, HOXA9, SOX17, and ZFP42 promoters	methylation	73.0	92.0	1.7	[91]
prostate cancer	urine	singleplex-miR-34b/c and miR-193b;multiplex-APC, GSTP1, and RARβ2 promoters	methylation	94.3	84.4	1.8	[27]
prostate cancer	urine	PLA2G16 CpG adjacent loci	methylation	92.0	35.0	1.3	[92]
prostate cancer	urine	GSTP1, SFRP2, IGFBP3, IGFBP7, APC, and PTSG2	methylation	90.0		0.9	[93]
prostate cancer	urine	miR-130a promoter	methylation	83.5	82.3	1.7	[94]
prostate cancer	urine	miR-24, miR-30c and CRIP3	methylation	81.0	59.7	1.4	[95]
prostate cancer	urine	GSTP1, APC, RASSF1A, PITX2 and C1orf114 p	methylation	81.0		0.8	[96]
prostate cancer	urine	GSTP1, SFRP2, IGFBP3, IGFBP7, APC, and PTGS2	methylation	73.0	76.0	1.5	[97]
prostate cancer	urine	GSTP1, RARB, and APC	methylation	55.0	80.0	1.4	[98]
prostate cancer	urine	GSTP1, RARB, and APC	methylation	53.0	76.0	1.3	[98]
prostate cancer	urine	DLEC1	methylation	36.7		0.4	[99]
prostate cancer	urine	HOXD3 and GSTP1	methylation	31.6	88.1	1.2	[7]

Green highlighted rows indicate the biomarker panels scoring highest in threshold score (>1.5) for each disease. * Threshold scores were calculated by log_10_ transformation of sensitivity and specificity.

## Data Availability

The original contributions presented in this study are included in the article/Supplementary Material. Further inquiries can be directed to the corresponding author.

## Supplementary Materials

The following supporting information can be downloaded at: https://www.mdpi.com/article/10.3390/life15030482/s1, Supplemental Figure S1. Publication Trends in Epigenetics in Urine and Feces PRISMA Literature Review Search; Supplemental Figure S2. PRISMA Systematic Literature Review Diagram of Liquid Biopsy (Urine and Feces) Epigenetic Marks; Supplemental Figure S3. Rank-ordered Epigenetic Biomarker Sensitivity Urine and Feces by Biomarker and Disease; Supplemental Figure S4. Epigenetic Biomarker Sensitivity and Specificity in Liquid Biopsy (Urine and Feces) by Publication Count; Supplemental Figure S5. Epigenetic Biomarker Sensitivity and Specificity in Liquid Biopsy (Urine and Feces) scaled by Sample Size; Supplemental Figure S6. Cancer Incidence Hotspots; Supplemental Table S1. Metadata of Epigenetic Biomarker Panel Studies include in this Review.

## Glossary

biomarker	biological compound produced in the body indicating disease
epigenetic	marks and changes (e.g., methylation, acetylation) to genetic machinery by environmental stimuli; not mutation
incidence	the rate of individuals diagnosed with a disease at a given time
liquid biopsy	analysis of bodily fluids to detect biomarkers of disease
sensitivity	ability for a test to indicate individuals with disease
specificity	ability for a test to indicate individuals without a disease
