# Supplementary material for: DNA Methylation in Urine and Feces Indicative of Eight Major Human Cancer Types Globally
Source: Life (Basel). 2025 Mar 17;15(3):482. doi: 10.3390/life15030482 (PMC11943902; doi:10.3390/life15030482)
Supplement: Supplementary file 1 [file life-15-00482-s001.zip › life-3417699-supplementary.pdf]

DNA Methylation in Urine and Feces Indicative of Eight Major Human Cancer Types Globally

Melanie Engstrom Newell [1,2,3]; Ayesha Babbrah [1,2,4]; Anumitha Aravindan [1,2,4]; Rathnam, R. [1,2,4] and Rolf U. Halden [1-5]

[1] Arizona State University, USA
[2] Biodesign Center for Environmental Health Engineering, USA
[3] School for Engineering of Matter, Transport and Energy, USA
[4] Barrett The Honors College, USA
[5] School of Sustainable Engineering and the Built Environment, USA
Address: Biodesign Institute Building B, 1001 S McAllister Ave, Tempe, AZ 85287
* Correspondence: author: Rolf U. Halden; rolf.halden@asu.edu

Supplemental Material Table of Contents

Supplemental Figure S1. Publication Trends in Epigenetics in Urine and Feces PRISMA Literature Review Search………………………………………………………………………… 2
Supplemental Figure S2. PRISMA Systematic Literature Review Diagram of Liquid Biopsy (Urine and Feces) Epigenetic Marks……………………………………………………………… 2
Supplemental Figure S3. Rank-ordered Epigenetic Biomarker Sensitivity Urine and Feces by Biomarker and Disease…………………………………………..……………………………… 3
Supplemental Figure S4. Epigenetic Biomarker Sensitivity and Specificity in Liquid Biopsy (Urine and Feces) by Publication Count…………………………………….…………………… 3
Supplemental Figure S5. Epigenetic Biomarker Sensitivity and Specificity in Liquid Biopsy (Urine and Feces) scaled by Sample Size…………………………………………………………4
Supplemental Figure S6. Cancer Incidence Hotspots………………………………………… 5-6
Supplemental Table S1. Metadata of Epigenetic Biomarker Panel Studies include in this Review……………………………………………………..………………………………..6-59

Supplemental Material

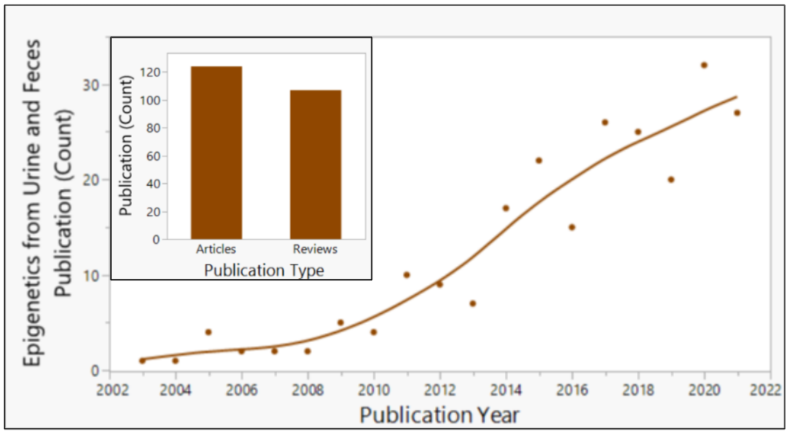

Supplemental Figure S1. Publication Trends in Epigenetics in Urine and Feces PRISMA Literature Review Search. (a) Line graph showing publications (count) over time of epigenetic marks in urine and feces. (b) The number of articles and reviews are compared in a histogram.

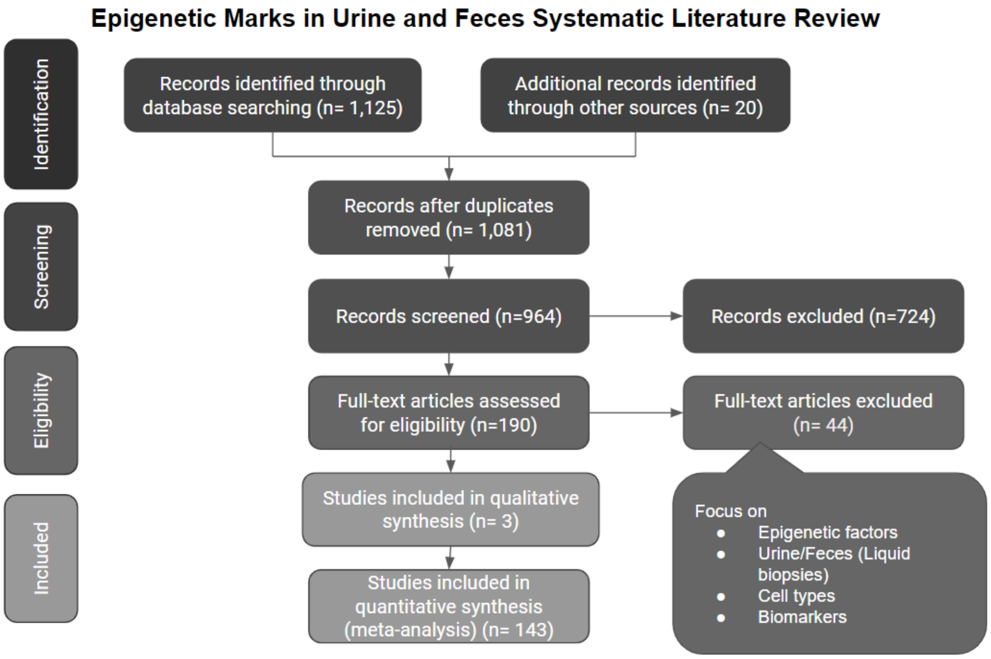

Supplemental Figure S2. PRISMA Systematic Literature Review Diagram of Liquid Biopsy (Urine and Feces) Epigenetic Marks. Key words: epigen* AND (fecal OR feces OR urine) AND cell AND biomarker

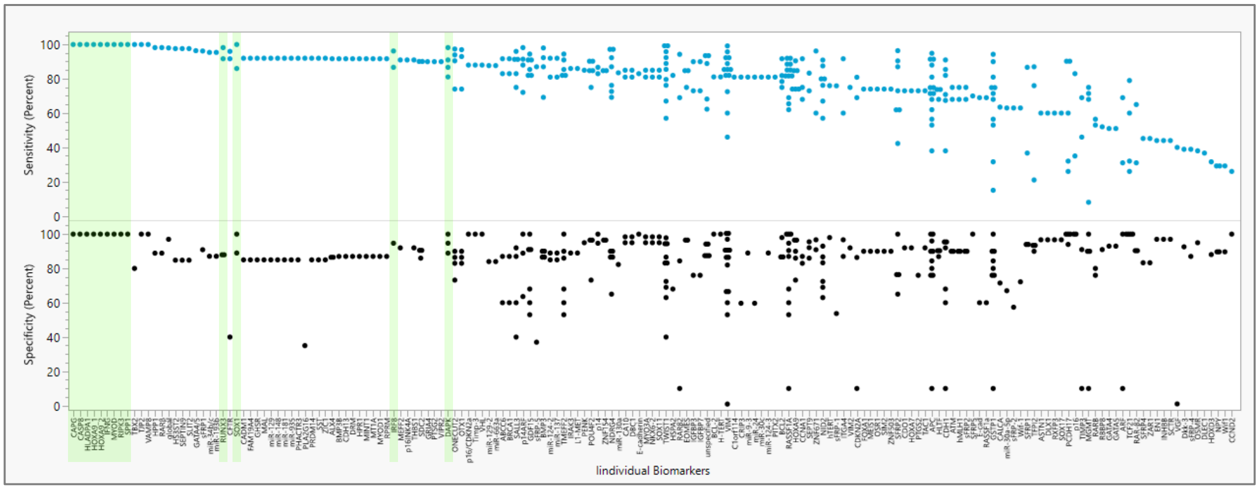

Supplemental Figure S3. Rank-ordered Epigenetic Biomarker Sensitivity Urine and Feces by Biomarker and Disease. Percent sensitivity and specificity reported by screening each epigenetic biomarker panel in liquid biopsy (urine and feces) are disaggregated into each biomarker**. Green highlight bars represent biomarker panels and individual biomarkers with the highest sensitivity and specificity given cancer type or replicability.
**Each biomarker represents the percent sensitivity and specificity reported from each panel in which it was present.

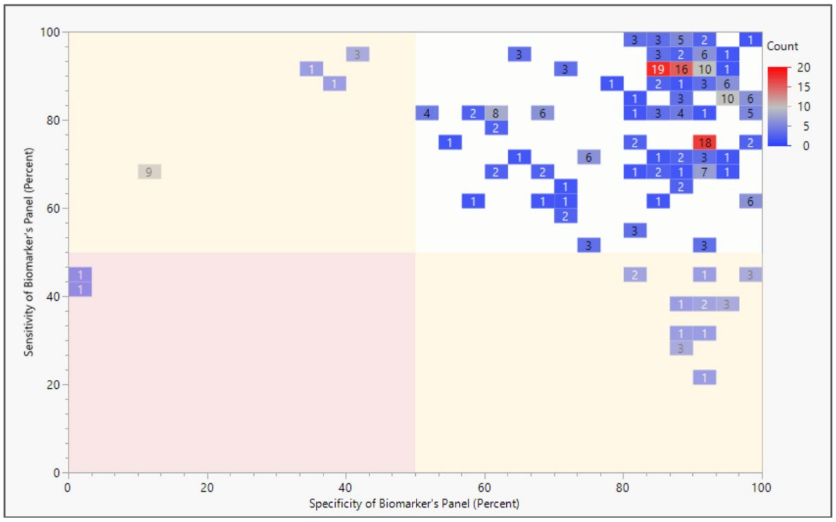

Supplemental Figure S4. Epigenetic Biomarker Sensitivity and Specificity in Liquid Biopsy (Urine and Feces) by Publication Count. Percent sensitivity and specificity are plotted against each other in a heatmap disaggregated into individual biomarkers* from the reported studies of liquid biopsy (urine and feces) represented in panel a. The red highlighted section represents sensitivity + specificity below the preferred 1.5 threshold, the yellow sections highlight studies meeting the 1.5 threshold, and the green section shows the studies above the 1.5 threshold.
*Each biomarker represents the percent sensitivity and specificity reported from each panel in which it was present.

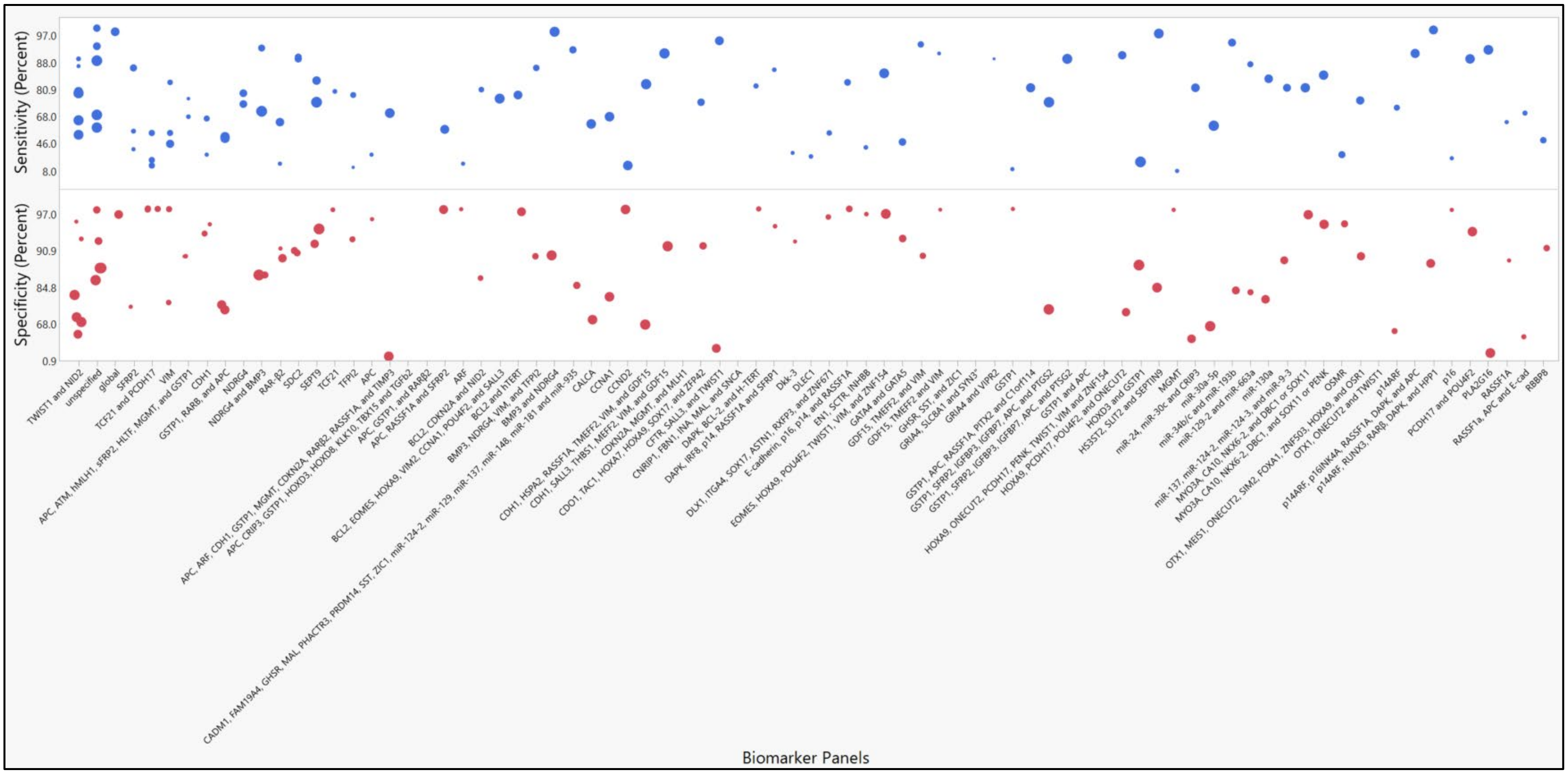

Supplemental Figure S5. Epigenetic Biomarker Sensitivity and Specificity in Liquid Biopsy (Urine and Feces) scaled by Sample Size. Biomarker panels are plotted by percent sensitivity (blue) and specificity (red) and sample size (patient count) ranging from n = 4 to n = 408.

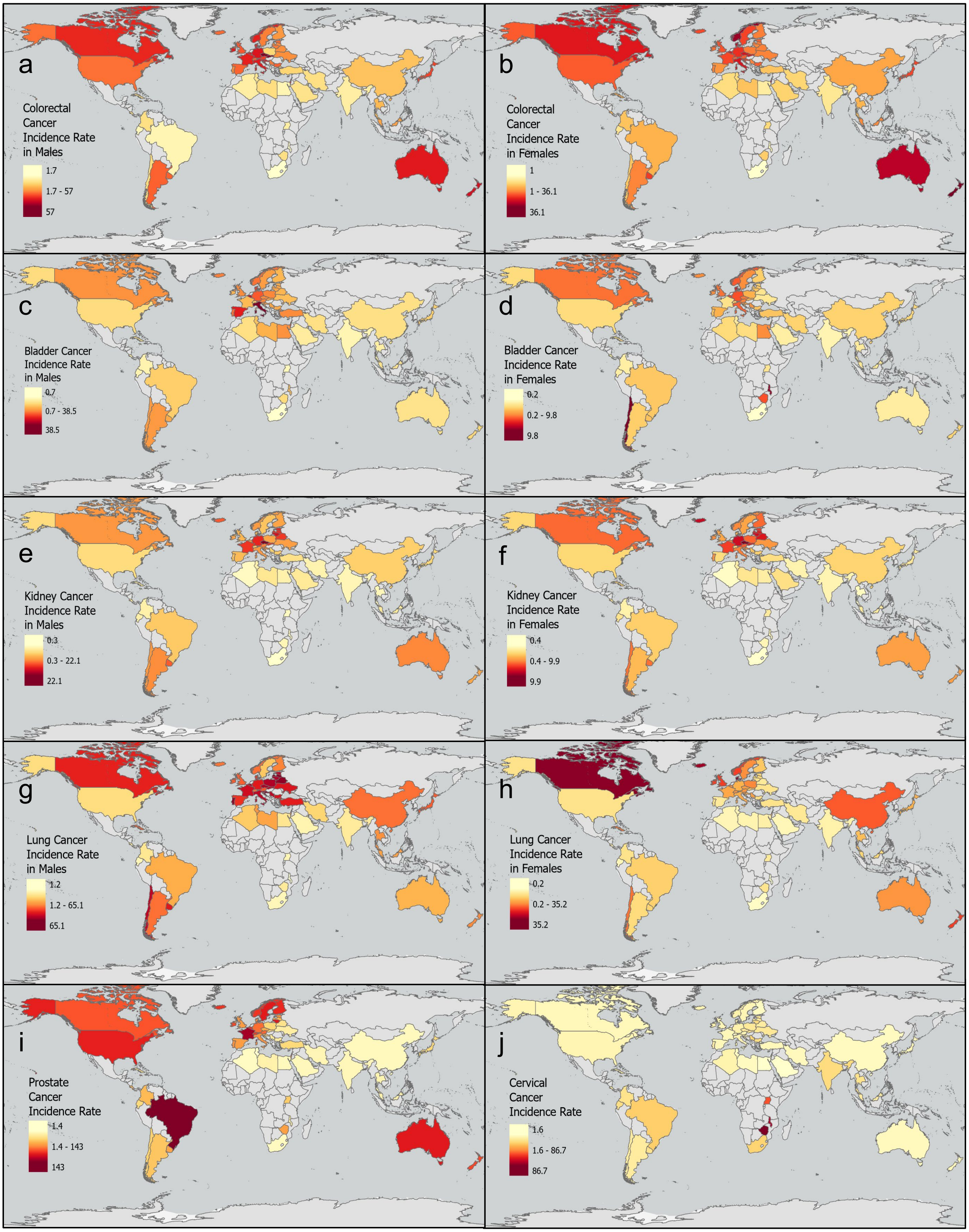
a
Colorectal Cancer Incidence Rate in Males
1.7
1.7 - 57
57
b
Colorectal Cancer Incidence Rate in Females
1
1 - 36.1
36.1
c
Bladder Cancer Incidence Rate in Males
0.7
0.7 - 38.5
38.5
d
Bladder Cancer Incidence Rate in Females
0.2
0.2 - 9.8
9.8
e
Kidney Cancer Incidence Rate in Males
0.3
0.3 - 22.1
22.1
f
Kidney Cancer Incidence Rate in Females
0.4
0.4 - 9.9
9.9
g
Lung Cancer Incidence Rate in Males
1.2
1.2 - 65.1
65.1
h
Lung Cancer Incidence Rate in Females
0.2
0.2 - 35.2
35.2
i
Prostate Cancer Incidence Rate
1.4
1.4 - 143
143
j
Cervical Cancer Incidence Rate
1.6
1.6 - 86.7
86.7

Supplemental Figure S6. Cancer Incidence Hotspots. Maps show incidence rates by country for colon and rectal combined to colorectal (a & b), bladder (c & d), kidney (e & f), lung (g & h), prostate (i), and cervical cancer (j). Incidence rates (per 100,000 people) reported by the Global Cancer Observatory are scaled by color, by sex, and country.

Supplemental Table S1. Metadata of Epigenetic Biomarker Panel Studies include in this Review

No.	Disease Details	Disease	Liquid biopsy specimen (i.e., Urine, Feces)	Sample size	Centrifuged sample	Fragment copies Details	RNA or DNA concentration (ug)	RNA or DNA concentration (uL)	Calculated RNA or DNA concentration (ug/mL)	RNA or DNA concentration Details	Extraction Method	Biomarker(s)	Epigenetic marker type	Epigenetic Assay	Epigenetic Frequency	Statistical Methods	Sensitivity (disease as positive) Details	Specificity (control as negative) Details	Patient (#) Details	Controls (#) Details	Geographic region(s)	Latitude (decimal)	Longitude (decimal)	Source	Comment
1	colorectal cancer, colorectal neoplasia	Colorectal Cancer	feces	36 g	12 mL	75 copies per reaction volume					direct gene capture from fecal supernatant	BMP3, NDRG4, vimentin, and TFPI2 plus mutant KRAS<br><br>quantitative fecal hemoglobin	methylation	rapid bisulfite treatment process QuARTS method<br><br>pre-commercial prototype sDNA assay		95% CI Fisher's exact binomial distribution<br><br>logistic regression	Neoplasm: 85% Adenomas: 82%<br><br>Cancer: 87% stage I and 2 both (86%), III (100%), and IV (75%) proximal: 92% distal: 81%	90% cutoff for positive	52	46	Rochester, Minnesota	44.016369	-92.475395	Ahlquist et al., 2012	
2	bladder cancer, urothelial carcinomas	Bladder Cancer	urine					1		1 ug Total RNA, 20 ng cDNA	ZR Urine DNA Isolation Kit ™	SFRP5	methylation	bisulfite modified EZ-DNA methylation kit<br><br>1 µg of total RNA Promega reverse transcription system Taq Polymerase ethidium bromide, UV light<br><br>Reference gene: GAPDH		p-value <0.05 Fisher exact test	70%	0% control	20	5	Aachen, Germany	50.775555	6.083611	Antony et al., 2010	
3	urothelial bladder cancer	Bladder Cancer	urine				2			2 µL bisulfite-treated DNA / 50uL	ZR Urine DNA Isolation KitTM	CDH1 promoter	methylation	~200 ng DNA/20 µL Bisulfite conversion EZ DNA Methylation™ Kit<br><br>MS-PCR Taq Polymerase	CDH1: 68.8% p14ARF: 72.9%<br><br>Control CDH1: 5.7% Control p14ARF: 37.1%	p-value 0.05 normal data distribution: Kolmogorov–Smirnov test nonparametric Spearman's rank correlation and	CDH1: 67.4% urine cytology: 34.9%	CDH1: 93.9% urine cytology: 93.9%	48	35	Samsun, Turkey	41.292782	36.33128	Bayramov et al., 2018	

																Mann–Whitney U-test<br>k test									
3	urothelial bladder cancer	Bladder Cancer	urine								ZR Urine DNA Isolation KitTM	p14ARF promoter	methylation	~200 ng DNA/20 µL<br>Bisulfite conversion EZ DNA Methylation™ Kit<br><br>MS-PCR<br>Taq Polymerase	CDH1: 68.8%<br>p14ARF: 72.9%<br><br>Control CDH1: 5.7%<br>Control p14ARF: 37.1%	p-value 0.05<br>normal data distribution: Kolmogorov–Smirnov test<br>nonparametric Spearman's rank correlation and Mann–Whitney U-test<br>k test	p14ARF: 72.1%<br>urine cytology: 34.9%	p14ARF: 63.6%<br>urine cytology: 93.9%	48	35	Samsun, Turkey	41.292782	36.33128	Bayramov et al., 2018	
4	colorectal cancer	Colorectal Cancer	feces	4 g		normal patients (median = 936 genome equivalents, range = 33–18 560 genome equivalents) and cancer patients (median = 1014 genome equivalents, range = 32–3700 genome equivalent					4 g stool sample, 20 µ L of RNase A Total DNA	Vimentin exon 1	methylation	vimentin fragments acrylamide gel–based affinity capture<br><br>MS-PCR<br>SybrGreen Mastermix		95% CI<br>log-odds ratios<br>Wald-type test<br>p <0.05	46%<br>stage I or II: 43%<br>stage II or IV: 50%<br>proximal to the splenic flexure: 46%<br>distal to the splenic flexure: 45%<br><br>~15 methylated cell detection limit	90%	94	198	Cleveland, Ohio	41.505493	-81.68129	Chen et al., 2005	
4	colorectal cancer	Colorectal Cancer	feces								4 g stool sample, 20 µ L of RNase A Total DNA	Vimentin exon 1	methylation	vimentin fragments acrylamide gel–based affinity capture<br><br>MS-PCR<br>SybrGreen Mastermix		95% CI<br>log-odds ratios<br>Wald-type test<br>p <0.05	46%<br>stage I or II: 43%<br>stage II or IV: 50%<br>proximal to the splenic flexure: 46%<br>distal to the splenic flexure:	90%	94	198	Burlington, Massachusetts	42.508045	-71.197929	Chen et al., 2005	

																	45%<br><br>~15 methylated cell detection limit								
5	bladder cancer	Bladder Cancer	urine	30-50 mL			0.5		0.0125	<500 ng of urinary cfDNA		global	methylation	500 ng of urinary cfDNA KAPA HTP Library Preparation Kit library preparation<br><br>bisulfite sequencing Illumina HiSeq 2500 sequencer<br><br>analysis pipeline Methy-Pipe	global methylation range: 61.1% - 73.5%<br><br>prportional cell contributions:<br>blood cells: 52% (0–84%)<br>kidney: 32% (7–100%)<br>urothelium: 5% (0–12%)				26 samples from 11 renal transplant patients<br>5 samples from 2 hematopoietic stem cell transplant (HSCT) patients		Hong Kong, China	22.302711	114.177216	Cheng et al., 2017	
6	bladder cancer	Bladder Cancer	urine	50 mL						100 uL DNA (outlier)	centrifuged, pellet washed twice with PBS buffer<br><br>QIAamp DNA Mini Kit	MYO3A, CA10, NKX6-2, and DBC1 or SOX11	methylation	bisulfite conversion EpiTect Bisulfite Kits<br><br>quantitative methylation-specific real-time PCR (qMSP)<br><br>internal control: mC-LESS		receiver operating characteristics (ROC) curve of the assay calculating the area under the curve (AUC) of ROC<br>$p < 0.05$	MYO3A, CA10, NKX6-2, and DBC1 or SOX11 panel: 81%	MYO3A, CA10, NKX6-2, and DBC1 or SOX11 panel: 97%	128	110	Houston, Texas	29.749907	-95.358421	Chung et al., 2011	All biomarkers: "MYO3A, CA10, SOX11, NKX6-2, PENK, and DBC1"
6	bladder cancer	Bladder Cancer	urine								centrifuged, pellet washed twice with PBS buffer<br><br>QIAamp DNA Mini Kit	MYO3A, CA10, NKX6-2, and DBC1 or SOX11	methylation	bisulfite conversion EpiTect Bisulfite Kits<br><br>quantitative methylation-specific real-time PCR (qMSP)<br><br>internal control: mC-LESS		receiver operating characteristics (ROC) curve of the assay calculating the area under the curve (AUC) of ROC<br>$p < 0.05$	MYO3A, CA10, NKX6-2, and DBC1 or SOX11 panel: 81%	MYO3A, CA10, NKX6-2, and DBC1 or SOX11 panel: 97%	128	110	Dallas, Texas	32.779167	-96.808891	Chung et al., 2011	All biomarkers: "MYO3A, CA10, SOX11, NKX6-2, PENK, and DBC1"
6	bladder cancer	Bladder Cancer	urine								centrifuged, pellet washed twice with PBS buffer<br><br>QIAamp DNA Mini Kit	MYO3A, CA10, NKX6-2, DBC1, and SOX11 or PENK	methylation	bisulfite conversion EpiTect Bisulfite Kits<br><br>quantitative methylation-specific real-time PCR (qMSP)<br><br>internal control: mC-LESS		receiver operating characteristics (ROC) curve of the assay calculating the area under the curve (AUC) of	MYO3A, CA10, NKX6-2, DBC1, and SOX11 or PENK panel: 85%	MYO3A, CA10, NKX6-2, DBC1, and SOX11 or PENK panel: 95%	128	110	Houston, Texas	29.749907	-95.358421	Chung et al., 2011	All biomarkers: "MYO3A, CA10, SOX11, NKX6-2, PENK, and DBC1"

																ROC<br>p < 0.05									
6	bladder cancer	Bladder Cancer	urine								centrifuged, pellet washed twice with PBS buffer<br><br>QIAamp DNA Mini Kit	MYO3A, CA10, NKX6-2, DBC1, and SOX11 or PENK	methylation	bisulfite conversion EpiTect Bisulfite Kits<br><br>quantitative methylation-specific real-time PCR (qMSP)<br><br>internal control: mC-LESS		receiver operating characteristics (ROC) curve of the assay calculating the area under the curve (AUC) of ROC<br>p < 0.05	MYO3A, CA10, NKX6-2, DBC1, and SOX11 or PENK panel: 85%	MYO3A, CA10, NKX6-2, DBC1, and SOX11 or PENK panel: 95%	128	110	Dallas, Texas	32.779167	-96.808891	Chung et al., 2011	All biomarkers: "MYO3A, CA10, SOX11, NKX6-2, PENK, and DBC1"
7	bladder cancer	Bladder Cancer	urine							1.5 mg of total RNA (outlier)	centrifuged, pellet washed twice with phosphate-buffered solution<br><br>phenol–chloroform procedure	GDF15, TMEFF2, and VIM CpG island-containing genes	methylation	bisulfite treatment<br><br>real-time quantitative MSP (qMSP) TaqMan Universal PCR Master Mix No AmpErase UNG<br><br>Reference gene: ALU	GDF15: 24/51 TMEFF2: 32/51 VIM: 40/51	Mann-Whitney Test<br>p < 0.001	94%	90%	51 BlCa patients	20 healthy individuals, 19 renal cancer patients 20 prostate cancer patients	Lisbon, Portugal	38.736946	-9.142685	Costa et al., 2010	
8	non-muscle invasive bladder cancer	Bladder Cancer	urine	10 mL							centrifuged, pellet analyzed	15 (proprietary)	methylation	methylation-sensitive restriction enzyme digestion<br><br>real-time PCR<br><br>Bladder EpiCheckTM software		sensitivity analysis<br><br>discrimination area under the receiver operating curve 95% CI<br><br>bias boostrapping<br><br>multivariate analysis calibration plots<br><br>robustness differential area under the curve	any cancer: 67.3% high-grade cancer: 88.9%	any cancer or high-grade cancer: 88%<br><br>negative predictive value (NPV) for any cancer: 94.4% high-grade cancer NPV: 99.3%<br><br>EpiScore TM positive threshold : ≥60	357		Vienna, Austria	48.210033	16.363449	D'Andrea et al., 2019	
9	bladder cancer	Bladder Cancer	urine	50 mL								transcription factor 21(TCF21)	methylation			p<0. 05			80	62 non-bladder cancer patients 40 healthy	Fujian Quanzhou, China	25.721292	119.384155	Ding et al., 2019	Article from ILL unreadable

																				individua<br>ls					
1<br>0	colorecta<br>l cancer	Colorect<br>al<br>Cancer	feces				0.05			50 ng/µl<br>DNA	QIAamp DNA Stool<br>Mini Kit	GRIA4,<br>SLC8A1<br>and<br>SYN3<br>CpG<br>islands	methyl<br>ation	bisulfite<br>conversion<br><br>digital PCR<br><br>methylation<br>percentage<br>BD Accuri C6	>1% of<br>methylatio<br>n<br>at least<br>one of the<br>three<br>markers:<br>87.5%<br>GRIA4<br>79.2%<br>SLC8A1:<br>70.8%<br>SYN3:<br>62.5%	logistic<br>regression<br>model<br>calculate<br>the area<br>under the<br>curve<br>(AUC)			24		Cagliari,<br>Sardinia,<br>Italy	39.22<br>7779	9.1111<br>11	Fadda<br>et al.,<br>2018	
1<br>0	colorecta<br>l cancer	Colorect<br>al<br>Cancer	feces								QIAamp DNA Stool<br>Mini Kit	GRIA4,<br>SLC8A1<br>and<br>SYN3<br>CpG<br>islands	methyl<br>ation	bisulfite<br>conversion<br><br>digital PCR<br><br>methylation<br>percentage<br>BD Accuri C6	>1% of<br>methylatio<br>n<br>at least<br>one of the<br>three<br>markers:<br>87.5%<br>GRIA4<br>79.2%<br>SLC8A1:<br>70.8%<br>SYN3:<br>62.5%	logistic<br>regression<br>model<br>calculate<br>the area<br>under the<br>curve<br>(AUC)			24		Sassari,<br>Italy	40.72<br>5925	8.5556<br>83	Fadda<br>et al.,<br>2018	
1<br>1	bladder<br>cancer	Bladder<br>Cancer	urine			at least<br>10<br>copies<br>of the<br>β-actin<br>gene						TWIST1<br>and NID2	methyl<br>ation	real-time<br>methylation-<br>specific<br>polymerase<br>chain reaction<br>(MS-PCR)<br><br>reference gene:<br>β-actin		95% CI<br><br>areas<br>under the<br>curve<br>TWIST1:<br>0.67<br>NID2:<br>0.64<br>combined<br>TWIST1<br>and NID2:<br>0.66	positive<br>threshold:<br>67%<br><br>smokers:<br>78%	positive<br>threshold<br>: 69%	209		Durham,<br>North<br>Carolina	35.99<br>4034	-<br>78.898<br>621	Fantony<br>et al.,<br>2015	
1<br>1	bladder<br>cancer	Bladder<br>Cancer	urine									TWIST1<br>and NID2	methyl<br>ation	real-time<br>methylation-<br>specific<br>polymerase<br>chain reaction<br>(MS-PCR)<br><br>reference gene:<br>β-actin		95% CI<br><br>areas<br>under the<br>curve<br>TWIST1:<br>0.67<br>NID2:<br>0.64<br>combined<br>TWIST1<br>and NID2:<br>0.66	positive<br>threshold:<br>67%<br><br>smokers:<br>78%	positive<br>threshold<br>: 69%	209		Norfolk,<br>Virginia	36.85<br>0769	-<br>76.285<br>873	Fantony<br>et al.,<br>2015	
1<br>2	bladder<br>cancer	Bladder<br>Cancer	urine			at least<br>10<br>copies<br>of the<br>β-actin<br>gene						TWIST1<br>and NID2	methyl<br>ation	real-time<br>methylation-<br>specific<br>polymerase<br>chain reaction<br>(MSPCR)<br><br>reference<br>gene: β-actin	positive<br>cytology:<br>AUC<br>0.704<br><br>positive<br>cytology<br>and DNA<br>methylatio	likelihood<br>ratio<br>Areas<br>under the<br>curve<br>(AUC)<br>Receiver<br>operator<br>characteri	positive<br>cytology<br>and DNA<br>methylatio<br>n assay:<br>0.57<br><br>current<br>smokers:	positive<br>cytology<br>and DNA<br>methylati<br>on<br>assay:<br>0.72	172		Durham,<br>North<br>Carolina	35.99<br>4034	-<br>78.898<br>621	Fantony<br>et al.,<br>2017	

															n assay: AUC 0.773 p < 0.001<br><br>negative cytology: AUC 0.558<br><br>negative cytology and DNA methylation assay: AUC 0.697 p = 0.003	stic (ROC) curves	78% compared to former: 56% non-smokers: 44%								
12	bladder cancer	Bladder Cancer	urine									TWIST1 and NID2	methylation	real-time methylation-specific polymerase chain reaction (MSPCR)<br><br>reference gene: β-actin	positive cytology: AUC 0.704<br><br>positive cytology and DNA methylation assay: AUC 0.773 p < 0.001<br><br>negative cytology: AUC 0.558<br><br>negative cytology and DNA methylation assay: AUC 0.697 p = 0.003	likelihood ratio Areas under the curve (AUC) Receiver operator characteristic (ROC) curves	positive cytology and DNA methylation assay: 0.57<br><br>current smokers: 78% compared to former: 56% non-smokers: 44%	positive cytology and DNA methylation assay: 0.72	172		Norfolk, Virginia	36.850769	-76.285873	Fantony et al., 2017	
13	bladder cancer	Bladder Cancer	urine				0.5			500 ng DNA	centrifuged, pellet washed with PBS buffer<br><br>DNeasy Blood and Tissue Kit	150 CpG loci	methylation	bisulphite sequencing RainDrop BS-seq UroMark<br><br>microdroplet PCR Taq polymerase		AUC: 97%<br><br>NPV: 97%	98%	97%	107	167	London, United Kingdom	51.509865	-0.118092	Feber et al., 2017	
13	bladder cancer	Bladder Cancer	urine								centrifuged, pellet washed with PBS buffer<br><br>DNeasy Blood and Tissue Kit	150 CpG loci	methylation	bisulphite sequencing RainDrop BS-seq UroMark<br><br>microdroplet PCR Taq polymerase		AUC: 97%<br><br>NPV: 97%	98%	97%	107	167	Birmingham, United Kingdom	52.489471	-1.898575	Feber et al., 2017	

14	urothelial carcinomas (UC)	Bladder Cancer	urine	50 mL							centrifuged, pellet washed twice with phosphate-buffered solution<br><br>QIAamp DNA Mini Kit	VIM, CDH1, SALL3, TMEFF2, RASSF1A, BRCA1, GDF15, and ABCC6	methylation	bisulfite treatment EpiTect Fast Bisulfite Conversion Kits<br><br>methylation-specific polymerase chain reaction (MSP)<br>2% agarose gel, ethidium bromide, UV<br><br>positive control: methylated human genomic DNA	odds ratios for diagnosis:<br><br>upper tract: univariable / multivariable<br>CDH1: 21.106 / 16.354<br>GDF15: 0.257 / 0.238<br>HSPA2: 2.713 / 2.913<br>RASSF1A: 2.550 / 5.490<br>TMEFF2: not significant / 2.864<br>VIM: 4.343 / 5.078<br><br>muscle invasive: uni / multi<br>GDF15: 0.214 / 0.214<br><br>high grade: uni / multi<br>ABCC6: 0.274 / 0.367<br>CDH1: 6.439 / 4.678	chi-square tests<br>logistic regression analyses<br>p < 0.05<br><br>area under the receiver operating characteristic (ROC) curve (AUC)	VIM, CDH1, SALL3, TMEFF2, RASSF1A, BRCA1, GDF15, and ABCC6: 0.83	VIM, CDH1, SALL3, TMEFF2, RASSF1A, BRCA1, GDF15, and ABCC6: 0.60	217	256	Xi Cheng Qu, Bei Jing Shi, China	39.858425	116.287148	Guo et al., 2018	All biomarkers: ABCC6, BRCA1, CDH1, GDF15, HSPA2, RASSF1A, SALL3, THBS1, TMEFF2, and VIM
14	urothelial carcinomas (UC)	Bladder Cancer	urine								centrifuged, pellet washed twice with phosphate-buffered solution<br><br>QIAamp DNA Mini Kit	CDH1, HSPA2, RASSF1A, TMEFF2, VIM, and GDF15	methylation	bisulfite treatment EpiTect Fast Bisulfite Conversion Kits<br><br>methylation-specific polymerase chain reaction (MSP)<br>2% agarose gel, ethidium bromide, UV<br><br>positive control: methylated human genomic DNA	odds ratios for diagnosis:<br><br>upper tract: univariable / multivariable<br>CDH1: 21.106 / 16.354<br>GDF15: 0.257 / 0.238<br>HSPA2: 2.713 / 2.913<br>RASSF1A: 2.550 / 5.490	chi-square tests<br>logistic regression analyses<br>p < 0.05<br><br>area under the receiver operating characteristic (ROC) curve (AUC)	CDH1, HSPA2, RASSF1A, TMEFF2, VIM, and GDF15: 0.82	CDH1, HSPA2, RASSF1A, TMEFF2, VIM, and GDF15: 0.68	217	256	Xi Cheng Qu, Bei Jing Shi, China	39.858425	116.287148	Guo et al., 2018	All biomarkers: ABCC6, BRCA1, CDH1, GDF15, HSPA2, RASSF1A, SALL3, THBS1, TMEFF2, and VIM

															TMEFF2: not significant / 2.864<br>VIM: 4.343 / 5.078<br><br>muscle invasive: uni / multi<br>GDF15: 0.214 / 0.214<br><br>high grade: uni / multi<br>ABCC6: 0.274 / 0.367<br>CDH1: 6.439 / 4.678										
14	urothelial carcinomas (UC)	Bladder Cancer	urine								centrifuged, pellet washed twice with phosphate-buffered solution<br><br>QIAamp DNA Mini Kit	VIM, RASSF1A, GDF15, and TMEFF2	methylation	bisulfite treatment EpiTect Fast Bisulfite Conversion Kits<br><br>methylation-specific polymerase chain reaction (MSP)<br>2% agarose gel, ethidium bromide, UV<br><br>positive control: methylated human genomic DNA	odds ratios for diagnosis:<br><br>upper tract: univariable / multivariable<br>CDH1: 21.106 / 16.354<br>GDF15: 0.257 / 0.238<br>HSPA2: 2.713 / 2.913<br>RASSF1A: 2.550 / 5.490<br>TMEFF2: not significant / 2.864<br>VIM: 4.343 / 5.078<br><br>muscle invasive: uni / multi<br>GDF15: 0.214 / 0.214<br><br>high grade: uni / multi<br>ABCC6: 0.274 / 0.367<br>CDH1:	chi-square tests<br>logistic regression analyses<br>p < 0.05<br><br>area under the receiver operating characteristic (ROC) curve (AUC)	VIM, RASSF1A, GDF15, and TMEFF2: 0.82	VIM, RASSF1A, GDF15, and TMEFF2: 0.53	217	256	Xi Cheng Qu, Bei Jing Shi, China	39.858425	116.287148	Guo et al., 2018	All biomarkers: ABCC6, BRCA1, CDH1, GDF15, HSPA2, RASSF1A, SALL3, THBS1, TMEFF2, and VIM

															6.439 / 4.678										
14	urothelial carcinomas (UC)	Bladder Cancer	urine								centrifuged, pellet washed twice with phosphate-buffered solution<br><br>QIAamp DNA Mini Kit	CDH1, SALL3, THBS1, MEFF2, VIM, and GDF15: 0.89, and with cytology	methylation	bisulfite treatment EpiTect Fast Bisulfite Conversion Kits<br><br>methylation-specific polymerase chain reaction (MSP)<br>2% agarose gel, ethidium bromide, UV<br><br>positive control: methylated human genomic DNA	odds ratios for diagnosis:<br><br>upper tract: univariable / multivariable<br>CDH1: 21.106 / 16.354<br>GDF15: 0.257 / 0.238<br>HSPA2: 2.713 / 2.913<br>RASSF1A: 2.550 / 5.490<br>TMEFF2: not significant / 2.864<br>VIM: 4.343 / 5.078<br><br>muscle invasive: uni / multi<br>GDF15: 0.214 / 0.214<br><br>high grade: uni / multi<br>ABCC6: 0.274 / 0.367<br>CDH1: 6.439 / 4.678	chi-square tests<br>logistic regression analyses<br>$p < 0.05$<br><br>area under the receiver operating characteristic (ROC) curve (AUC)	CDH1, SALL3, THBS1, MEFF2, VIM, and GDF15: 0.89, and with cytology: 0.91	CDH1, SALL3, THBS1, TMEFF2, VIM, and GDF15: 0.74, and with cytology: 0.92	217	256	Xi Cheng Qu, Bei Jing Shi, China	39.858425	116.287148	Guo et al., 2018	All biomarkers: ABCC6, BRCA1, CDH1, GDF15, HSPA2, RASSF1A, SALL3, THBS1, TMEFF2, and VIM
15	urothelial cell carcinoma	Bladder Cancer	urine							(not shared for urine)		VGF	methylation	EpiTect Bisulfite Kit<br><br>PCR Taq Polymerase		Fisher's exact test $p = 0.020$	40%	Control: 5%	20	20	Baltimore, Maryland	39.299236	-76.609383	Hayashi et al., 2014	
16	bladder cancer	Bladder Cancer	urine	10-100 mL			0.02		0.0004	20 ng/µl	centrifuged, pellet suspended in phosphate-buffered saline<br><br>QiAamp DNA micro-kit	TWIST1 and NID2	methylation	qPCR MethyLight assay	odds ratio for detection multivariate<br><br>cancer:<br>NID2: 1.06<br>TWIST1: 1.25<br>age: 1.04<br><br>grade:	Kruskal-Wallis and Fisher's exact test logistic regression models<br><br>$p < 0.05$<br><br>area under the receiver	cancer:<br>NID2: 76.8%<br>TWIST1: 70.8%<br>both NID2 & TWIST1: 76.2%<br><br>grade:<br>NID2: 77.8%	cancer:<br>NID2: 61.56%, PPV: 81.46%, NPV: 55.96%<br>TWIST1: 85.4%, PPV: 92.12, NPV: 58.03%	212	102	Toronto, Canada	43.65107	-79.347015	Hermanns et al., 2020	All biomarkers: TWIST1, RUNX3, GATA4, NID2, and FOXE1

															TWIST1: 1.04	operating characteristics curves (AUC).	TWIST1: 70.8%<br>both NID2 & TWIST1: 77.6%	both NID2 & TWIST1: 83.33%<br><br>grade:<br>NID2: 60.2%, PPV: 81.37%, NPV: 58.31%<br>TWIST1: 85.3%, PPV: 90.86, NPV: 56.94%<br>both NID2 & TWIST1: 61.1%							
16	bladder cancer	Bladder Cancer	urine								centrifuged, pellet suspended in phosphate-buffered saline<br><br>QiAamp DNA micro-kit	TWIST1 and NID2	methylation	qPCR MethyLight assay	odds ratio for detection multivariate<br><br>cancer:<br>NID2: 1.06<br>TWIST1: 1.25<br>age: 1.04<br><br>grade:<br>TWIST1: 1.04	Kruskal-Wallis and Fisher's exact test logistic regression models<br><br>p < 0.05<br><br>area under the receiver operating characteristics curves (AUC).	cancer:<br>NID2: 76.8%<br>TWIST1: 70.8%<br>both NID2 & TWIST1: 76.2%<br><br>grade:<br>NID2: 77.8%<br>TWIST1: 70.8%<br>both NID2 & TWIST1: 77.6%	cancer:<br>NID2: 61.56%, PPV: 81.46%, NPV: 55.96%<br>TWIST1: 85.4%, PPV: 92.12, NPV: 58.03%<br>both NID2 & TWIST1: 83.33%<br><br>grade:<br>NID2: 60.2%, PPV: 81.37%, NPV: 58.31%<br>TWIST1: 85.3%, PPV: 90.86, NPV: 56.94%<br>both NID2 & TWIST1: 61.1%	212	102	Halifax, Canada	44.65107	-63.582687	Hermanns et al., 2020	All biomarkers: TWIST1, RUNX3, GATA4, NID2, and FOXE1
16	bladder cancer	Bladder Cancer	urine								centrifuged, pellet suspended in phosphate-buffered saline<br><br>QiAamp DNA micro-kit	TWIST1 and NID2	methylation	qPCR MethyLight assay	odds ratio for detection multivariate<br><br>cancer:<br>NID2: 1.06<br>TWIST1: 1.25	Kruskal-Wallis and Fisher's exact test logistic regression models<br><br>p < 0.05	cancer:<br>NID2: 76.8%<br>TWIST1: 70.8%<br>both NID2 & TWIST1: 76.2%	cancer:<br>NID2: 61.56%, PPV: 81.46%, NPV: 55.96%<br>TWIST1: 85.4%, PPV:	212	102	Zurich, Switzerland	47.373878	8.545094	Hermanns et al., 2020	All biomarkers: TWIST1, RUNX3, GATA4, NID2, and FOXE1

															age: 1.04<br><br>grade: TWIST1: 1.04	area under the receiver operating characteristics curves (AUC).	grade: NID2: 77.8% TWIST1: 70.8% both NID2 & TWIST1: 77.6%	92.12, NPV: 58.03% both NID2 & TWIST1: 83.33%<br><br>grade: NID2: 60.2%, PPV: 81.37%, NPV: 58.31% TWIST1: 85.3%, PPV: 90.86, NPV: 56.94% both NID2 & TWIST1: 61.1%							
17	cervical cancer	Cervical Cancer	urine	10-50 mL			0.02		0.0007	at least 20 ng/µL	EZNA Tissue DNA Extraction kit	SFRP4 and ZAR1 promoters	methylation	DNA integrity quantified with 268-base pair fragment of β-globin (HBB)<br><br>bisulfite modification EZ DNA Methylation Kit-GoldTM<br><br>quantitative methylation-specific PCR (QMSP) Taq Polymerase	SFRP4: non-lesion (mean = 0) L-SIL (mean = 0.04098) H-SIL (mean = 0.07513) SCC (mean = 4.98)<br><br>ZAR1: not significant differences between groups	chi-square test Fisher's exact test Kruskal Wallis test and Dunn’s post-test Cohen’s kappa coefficient<br><br>ROC AUC: 0.633<br><br>p<0.005	45.16%	83.30%	40 low-grade squamous intraepithelial lesions (L-SIL) 40 high-grade squamous intraepithelial lesions (H-SIL) 31 cervical squamous cell carcinoma (SCC)	60 non-lesions	Temuco, Chile	-38.73965	-72.59842	Hoffstetter et al., 2017	
18	colorectal cancer	Colorectal Cancer	feces			<10 copies DNA						NDRG4 and BMP3	methylation	multitarget stool DNA test		a logistic-regression algorithm positive threshold: 183<br><br>p<0.001	cancer: 92.3%	nonadvanced or negative findings: 86.6%	65 colorectal patients	9167 nonadvanced or negative findings	United States	38.889722	-77.008889	Imperiale et al., 2014	90 sites in U.S. and Canada
18	colorectal cancer	Colorectal Cancer	feces									NDRG4 and BMP3	methylation	multitarget stool DNA test		a logistic-regression algorithm positive threshold: 183<br><br>p<0.001	cancer: 92.3%	nonadvanced or negative findings: 86.6%	65 colorectal patients	9167 nonadvanced or negative findings	Canada	56.130366	-106.346771	Imperiale et al., 2014	

18	colorectal cancer	Colorectal Cancer	feces									NDRG4 and BMP3	methylation	multitarget stool DNA test		a logistic-regression algorithm positive threshold: 183<br><br>p<0.001	polyps with high grade dysplasia: 69.2% serrated sessile polyps: 42.4%	nonadvanced or negative findings: 86.6%	757 with advanced adenomas or polyps	9167 nonadvanced or negative findings	United States	38.889722	-77.008889	Imperiale et al., 2014	
18	colorectal cancer	Colorectal Cancer	feces									NDRG4 and BMP3	methylation	multitarget stool DNA test		a logistic-regression algorithm positive threshold: 183<br><br>p<0.001	polyps with high grade dysplasia: 69.2% serrated sessile polyps: 42.4%	nonadvanced or negative findings: 86.6%	757 with advanced adenomas or polyps	9167 nonadvanced or negative findings	Canada	56.130366	-106.346771	Imperiale et al., 2014	
19	urothelial carcinoma	Bladder Cancer	urine			at least 10 copies of the control ACTB gene						TWIST1 and NID2	methylation	methylation-specific polymerase chain reaction (MSPCR)<br><br>reference gene: β-Actin		logistic regression model<br><br>AUC of ROC<br><br>95% CI	79% optimized: 75%	63% optimized: 71%	111		Durham, North Carolina	35.994034	-78.898621	Abern et al., 2014	
20	bladder cancer	Bladder Cancer	urine				0.5			maximum 500 ng	centrifuged, pellet washed in PBS filtered<br><br>Qiagen Mini Prep kit<br><br>concentration analysis: NanoDrop spectrophotometer	BCL2, EOMES, HOXA9, VIM2, CCNA1, POU4F2, and SALL3	methylation	bisulfite treatment EZ DNA MethylationGold Kit<br><br>Real-time quantitative methylation-specific PCR MethyLight<br><br>reference gene: ALUC4 positive control: bisulfite treated Human Genomic DNA<br><br>pyrosequencing assays			75%		189	11	Herlev, Denmark	55.72876	12.43728	Andersson et al., 2014	
21	Non–Small Cell Lung Cancer	Lung Cancer	urine	10 mL							centrifuged, preserved in EDTA<br><br>Proteinase K, Buffer AL isopropanol, Magnetic Beads	CDO1, TAC1, HOXA7, HOXA9, SOX17, and ZFP42 promoters	methylation	methylation on beads (MOB) protocol bisulfite conversionmethylation-specific PCR (qMSP)<br><br>reference gene: β-Actin	Concordance: 78.3%	95% CI<br><br>Wilcoxon rank sum test Fisher exact test Pearson correlation analysis	methylation in 3 or more genes: 73%	methylation in 3 or more genes: 92%	74	27	Baltimore, Maryland	39.299236	-76.609383	B. Liu et al., 2020	
21	Non–Small Cell Lung Cancer	Lung Cancer	urine								centrifuged, preserved in EDTA<br><br>Proteinase K, Buffer AL isopropanol, Magnetic Beads	CDO1, TAC1, HOXA7, HOXA9, SOX17, and ZFP42	methylation	methylation on beads (MOB) protocol bisulfite conversionmethylation-specific PCR	Concordance: 78.3%	95% CI<br><br>Wilcoxon rank sum test Fisher exact test	methylation in 3 or more genes: 73%	methylation in 3 or more genes: 92%	74	27	Chicago, Illinois	41.881832	-87.623177	B. Liu et al., 2020	

												promoter<br>s		(qMSP)<br><br>reference<br>gene: β-Actin		Pearson<br>correlation<br>analysis									
2<br>2	colorecta<br>l cancer	Colorect<br>al<br>Cancer	urine				0.4			up to<br>400 ng<br>DNA	centrifuged, pellet or<br>unfractionated<br>analyzed<br><br>Quick DNA urine kit<br><br>concentration<br>analysis: Qubit™<br>dsDNA<br>HS Assay	SEPT9,<br>TMEFF2,<br>SDC2,<br>NDRG4,<br>VIM and<br>ALX4	methyl<br>ation	bisulfite<br>conversion<br>EZ DNA<br>Methylation kit<br><br>quantitative<br>Methylation<br>Specifc PCRs<br>Epitect<br>Multiplex PCR<br>Mastermix<br><br>reference<br>gene: ACTB	SEPT9<br>and<br>SDC2:<br>70%<br>cases	Mann<br>Whitney U<br>test<br>Pearson's<br>Chi-square<br>test<br>p v<0.05,<br>Bonferroni<br>correction<br><br>Kruskal<br>Wallis test<br><br>multivariate<br>logistic<br>regression<br><br>Receiver<br>Operator<br>Characteris<br>tic (ROC)<br>curve		SEPT9<br>and<br>SDC2:<br>86%	92	63	Amsterd<br>am, The<br>Netherla<br>nds	52.37<br>7956	4.8970<br>7	Bach et<br>al.,<br>2021	
2<br>3	kidney<br>cancer	Kidney<br>Cancer	urine	200–<br>500<br>µl			0.25		1.0000	0.25–1<br>µg DNA	centrifuged, pellet<br>analyzed<br><br>proteinase K<br>phenol/chloroform<br>extraction<br>ethanol precipitation	VHL,<br>p16/CDK<br>N2a,<br>p14ARF,<br>APC,<br>RASSF1<br>A, and<br>Timp-3	methyl<br>ation	bisulfite<br>treatment<br><br>methylation-<br>specific PCR		Fisher's<br>exact test<br><br>P ≤ 0.05	88%	100%	50	12<br>healthy<br>individua<br>ls<br>9<br>nephrolit<br>hiasis<br>(renal<br>stones)<br>3 benign<br>renal<br>cysts	Philadelp<br>hia,<br>Pennsylv<br>ania	39.95<br>2583	-<br>75.165<br>222	Battagli<br>et al.,<br>2003	
2<br>4	bladder<br>cancer	Bladder<br>Cancer	urine	2 to<br>40ml			0.05		0.0026	50ng<br>DNA	Quick-DNA™ Urine<br>Kit	CADM1,<br>FAM19A<br>4,<br>GHSR,<br>MAL,<br>PHACTR<br>3,<br>PRDM14<br>, SST,<br>ZIC1,<br>miR-124-<br>2, miR-<br>129,<br>miR-137,<br>miR-148,<br>miR-181<br>and miR-<br>935	methyl<br>ation	bisulfite<br>treatment<br>EZ DNA<br>Methylation™<br>kit<br><br>quantitative<br>methylation-<br>specific PCR<br><br>reference<br>gene: ACTB		Mann-<br>WhitneyUt<br>est<br>chi-square<br>test<br><br>Multivariat<br>e logistic<br>regression<br>analysis<br><br>95% CI	92%	85%	72	75	Amsterd<br>am, The<br>Netherla<br>nds	52.37<br>7956	4.8970<br>7	Bosschi<br>eter et<br>al.,<br>2019	
2<br>5	bladder<br>cancer	Bladder<br>Cancer	urine					4		4uL DNA	Tissue and Cell<br>Genomic DNA<br>Purification Kit	DAPK,<br>IRF8,<br>p14,<br>RASSF1<br>A and<br>SFRP1	methyl<br>ation	bisulfite<br>treatment<br>EZ DNA<br>Methylation<br>Kit<br><br>quantitative<br>MSP<br>Taq<br>Polymerase		Kruskal-<br>Wallis<br>Test<br>Mann-<br>Whitney<br>test<br><br>chi-square<br>test<br>Fisher's	86.70%	94.70%	30	19	Taiwan,<br>China	23.69<br>781	120.96<br>051	Chen et<br>al.,<br>2011	

														unmethylated positive control: human peripheral lymphocyte DNA		exact test<br><br>AUC of ROC<br><br>Kaplan-Meier analysis log-rank test<br><br>P < 0.05									
25	bladder cancer	Bladder Cancer	urine								Tissue and Cell Genomic DNA Purification Kit	DAPK, IRF8, p14, RASSF1A and SFRP1	methylation	bisulfite treatment EZ DNA Methylation Kit<br><br>quantitative MSP Taq Polymerase<br><br>unmethylated positive control: human peripheral lymphocyte DNA		Kruskal-Wallis Test Mann-Whitney test<br><br>chi-square test Fisher's exact test<br><br>AUC of ROC<br><br>Kaplan-Meier analysis log-rank test<br><br>P < 0.05	86.70%	94.70%	30	19	Hong Kong, China	22.302711	114.177216	Chen et al., 2011	
26	bladder cancer	Bladder Cancer	urine	20 mL			0.5		0.0250	<500 ng of urinary cfDNA	centrifugation filtration	cfDNA	methylation<br><br>copy number	bisulfite conversion EpiTect Bisulfite Kit<br><br>KAPA HTP Library Preparation Kit Shallow-depth paired-end genome-wide bisulfite sequencing		Mann–Whitney U-test<br><br>95% CI	Methylomic and copy number: 93.5%	Methylomic and copy number: 95.8%	46	39	Hong Kong, China	22.302711	114.177216	Cheng et al., 2019	
27	urothelial cancer	Bladder Cancer	urine	50 mL			2		0.0400	2 ug DNA	centrifuged, pellet washed in PBS	hyper: SOX1, TJP2, MYOD, HOXA9_1, HOXA9_2<br><br>hypo: VAMP8, CASP8, SPP1, IFNG, CAPG, HLADPA1, RIPK3	methylation	bisulfite treatment Epitect Bisulfite Kit (Qiagen)<br><br>bisulfite pyrosequencing GoTaq Hot Start Polymerase		Pearson correlation coefficient Mann–Whitney test<br><br>AUC of ROC	100%	100%	73	18	Los Angeles, California	34.052235	-118.243683	Chihara et al., 2013	

27	urothelial cancer	Bladder Cancer	urine								centrifuged, pellet washed in PBS	hyper: SOX1, TJP2, MYOD, HOXA9_1, HOXA9_2<br><br>hypo: VAMP8, CASP8, SPP1, IFNG, CAPG, HLADPA1, RIPK3	methylation	bisulfite treatment Epitect Bisulfite Kit (Qiagen)<br><br>bisulfite pyrosequencing<br>GoTaq Hot Start Polymerase		Pearson correlation coefficient Mann–Whitney test<br><br>AUC of ROC	100%	100%	73	18	Nara, Japan	34.68505	135.80485	Chihara et al., 2013	
27	urothelial cancer	Bladder Cancer	urine								centrifuged, pellet washed in PBS	hyper: SOX1, TJP2, MYOD, HOXA9_1, HOXA9_2<br><br>hypo: VAMP8, CASP8, SPP1, IFNG, CAPG, HLADPA1, RIPK3	methylation	bisulfite treatment Epitect Bisulfite Kit (Qiagen)<br><br>bisulfite pyrosequencing<br>GoTaq Hot Start Polymerase		Pearson correlation coefficient Mann–Whitney test<br><br>AUC of ROC	100%	100%	73	18	Tokyo, Japan	35.652832	139.839478	Chihara et al., 2013	
27	urothelial cancer	Bladder Cancer	urine								centrifuged, pellet washed in PBS	hyper: SOX1, TJP2, MYOD, HOXA9_1, HOXA9_2<br><br>hypo: VAMP8, CASP8, SPP1, IFNG, CAPG, HLADPA1, RIPK3	methylation	bisulfite treatment Epitect Bisulfite Kit (Qiagen)<br><br>bisulfite pyrosequencing<br>GoTaq Hot Start Polymerase		Pearson correlation coefficient Mann–Whitney test<br><br>AUC of ROC	100%	100%	73	18	Tochigi, Japan	36.455254	139.804108	Chihara et al., 2013	
28	prostate cancer	Prostate Cancer	urine	30 mL	4.5 mL		0.015		0.0005	15-20 ng RNA	centrifuge, pellet analyzed<br><br>concentration analysis: NanoString	GSTP1, SFRP2, IGFBP3, IGFBP7, APC, and PTSG2	methylation	quantitative methylation-specific polymerase chain reaction (qPCR)	proportional odds ratio = 2.04 per 0.1 ExoMeth increase<br><br>Gleason ≥3 + 4: AUC 0.89 Gleason≥4 + 3: AUC 0.81	logistic regression<br><br>95% CI	90% accuracy		197		Dublin, Ireland	53.35014	-6.266155	Connell et al., 2020	

28	prostate cancer	Prostate Cancer	urine								centrifuge, pellet analyzed<br><br>concentration analysis: NanoString	GSTP1, SFRP2, IGFBP3, IGFBP7, APC, and PTSG2	methylation	quantitative methylation-specific polymerase chain reaction (qPCR)	proportional odds ratio = 2.04 per 0.1 ExoMeth increase<br><br>Gleason ≥3 + 4: AUC 0.89 Gleason≥4 + 3: AUC 0.81	logistic regression<br><br>95% CI	90% accuracy		197		Toronto, Ontario, Canada	43.65107	-79.347015	Connell et al., 2020	
28	prostate cancer	Prostate Cancer	urine								centrifuge, pellet analyzed<br><br>concentration analysis: NanoString	GSTP1, SFRP2, IGFBP3, IGFBP7, APC, and PTSG2	methylation	quantitative methylation-specific polymerase chain reaction (qPCR)	proportional odds ratio = 2.04 per 0.1 ExoMeth increase<br><br>Gleason ≥3 + 4: AUC 0.89 Gleason≥4 + 3: AUC 0.81	logistic regression<br><br>95% CI	90% accuracy		197		Norwich, Norfolk, United Kingdom	52.630886	1.297355	Connell et al., 2020	
29	bladder cancer	Bladder Cancer	urine	20–50 mL			1		0.0286	at least 1 ug DNA	centrifuged, pellet washed in phosphate-buffered saline<br><br>phenol-chloroform procedure	TCF21 and PCDH17 promoters	methylation	bisulfite treatment<br><br>real-time quantitative methylation-specific polymerase chain reaction (qMSP) AmpliTaq Gold<br><br>reference gene: ACTB		Kruskall-Wallis test Mann-Whitney U-test Kruskall-Wallis tests Spearman nonparametric correlation test<br><br>Receiver operator characteristics (ROC) curve, area under the curve (AUC)<br><br>P < 0.05	bladder 60%<br><br>overall: 39%	100%	50 bladder cancer	48 healthy individuals	Manassas, Virginia	38.75066	-77.475143	Costa et al., 2011	
29	bladder cancer	Bladder Cancer	urine								centrifuged, pellet washed in phosphate-buffered saline<br><br>phenol-chloroform procedure	TCF21 and PCDH17 promoters	methylation	bisulfite treatment<br><br>real-time quantitative methylation-specific polymerase chain reaction (qMSP) AmpliTaq Gold<br><br>reference gene: ACTB		Kruskall-Wallis test Mann-Whitney U-test Kruskall-Wallis tests Spearman nonparametric correlation test	renal: 32%<br><br>overall: 39%	100%	50 renal cancer	48 healthy individuals	Manassas, Virginia	38.75066	-77.475143	Costa et al., 2011	

																Receiver operator characteristics (ROC) curve, area under the curve (AUC)<br><br>$P < 0.05$									
29	bladder cancer	Bladder Cancer	urine								centrifuged, pellet washed in phosphate-buffered saline<br><br>phenol-chloroform procedure	TCF21 and PCDH17 promoters	methylation	bisulfite treatment<br><br>real-time quantitative methylation-specific polymerase chain reaction (qMSP) AmpliTaq Gold<br><br>reference gene: ACTB		Kruskall-Wallis test Mann-Whitney U-test Kruskall-Wallis tests Spearman nonparametric correlation test<br><br>Receiver operator characteristics (ROC) curve, area under the curve (AUC)<br><br>$P < 0.05$	prostate: 26%<br><br>overall: 39%	100%	50 prostate cancer	48 healthy individuals	Manassas, Virginia	38.75066	-77.475143	Costa et al., 2011	
30	bladder cancer	Bladder Cancer	urine	30–60 mL							centrifuged, pellet washed in phosphate-buffered saline<br><br>QIAamp DNA Mini Kit	RAR-β2	methylation	bisulfite treatment<br>EZ DNA Methylation-Gold™ kit<br><br>methylation specific nested PCR<br>puReTaq Polymerase<br>2% agarose gel, ethidium bromide<br><br>positive control: Universal methylated human liver DNA standard		Kruskal–Wallis<br>Mann–Whitney U<br>non-parametric tests<br>chi-square test<br><br>ROC, AUC<br><br>$P < 0.05$	65%	89.70%	100	65 benign urological disorders<br>51 healthy volunteers	Cairo, Egypt	30.033333	31.233334	Eissa et al., 2012	
31	bladder cancer	Bladder Cancer	urine					18		18 µl of DNA	centrifuged, pellet analyzed	DAPK, BCL-2, and H-TERT	methylation	bisulfite treatment<br><br>real-time quantitative methylation-sensitive PCR (MethyLight)		Kaplan-Meier analysis<br>log-rank test<br>Kaplan-Meier curve	81.10%	100%	37	20	Hamburg, Germany	53.551086	9.993682	Friedrich et al., 2007	

														control: Sss1-treated DNA methylated with methylase		P < 0.05									
32	colorectal cancer	Colorectal Cancer	feces	4 g			2			2 ug DNA	centrifuged, RNase A sodium acetate-isopropanol, ethanol wash,TE<br>proteinase K phenol/chloroform/isoamyl alcohol, LoTE<br><br>QIAamp DNA stool mini kit	TFPI2	methylation	bisulfite modifed Zymo Kit<br><br>Quantitative methylation-specific PCR Taq polymerase		Receiver operator characteristic (ROC) curve analysis, area under the curve	CRC: 76%<br>PPV: 94%	CRC: 93%<br>NPV: 73%	47 CRC	30	Amsterdam, The Netherlands	52.377956	4.89707	Glöckner et al., 2009	
32	colorectal cancer	Colorectal Cancer	feces								centrifuged, RNase A sodium acetate-isopropanol, ethanol wash,TE<br>proteinase K phenol/chloroform/isoamyl alcohol, LoTE<br><br>QIAamp DNA stool mini kit	TFPI2	methylation	bisulfite modifed Zymo Kit<br><br>Quantitative methylation-specific PCR Taq polymerase		Receiver operator characteristic (ROC) curve analysis, area under the curve	adenomas:<br>21%<br>PPV: 67%	adenomas:<br>93%<br>NPV: 65%	19 adenomas	30	Amsterdam, The Netherlands	52.377956	4.89707	Glöckner et al., 2009	
33	colorectal cancer	Colorectal Cancer	feces	4 g			2			2 ug DNA		GATA4 and GATA5 promoters	methylation	Quantitative MSP (qMSP) Taq polymerase		Pearson's chi-square test<br>Fisher's exact test and<br>the one-way ANOVA<br>Kruskal-Wallis<br>Mann-Whitney test<br><br>Mc Nemar test<br>paired t-test<br><br>Receiver operator characteristic (ROC) curve, area under the curve (AUC)<br><br>95% CI	51%	93%	75	75	Maastricht, The Netherlands	50.851368	5.690973	Hellebrekers et al., 2009	
34	kidney cancer	Kidney Cancer	urine	15 mL			2		0.1333	2 ug DNA	centrifuged, pellet analyzed<br><br>proteinase K phenol/chloroform extraction<br>ethanol precipitation	CDH1 promoter	methylation	bisulfite treatement<br><br>quantitative fluorogenic real-time PCR TaqMan	at least one gene methylated: 88%		Clinical: CDH1: 38%	CDH1: 95%	17 primary kidney cancer<br>9 renal cancer	91 without genitourinary cancer	Baltimore, Maryland	39.299236	-76.609383	Hoque et al., 2004	

34	kidney cancer	Kidney Cancer	urine								centrifuged, pellet analyzed<br><br>proteinase K phenol/chloroform extraction ethanol precipitation	APC promoter	methyl ation	bisulfite treatement<br><br>quantitative fluorogenic real-time PCR TaqMan	at least one gene methylate d: 88%		Clinical: APC: 38%	APC: 96%	17 primary kidney cancer 9 renal cancer	91 without genitouri nary cancer	Baltimore , Maryland	39.29 9236	- 76.609 383	Hoque et al., 2004	
34	kidney cancer	Kidney Cancer	urine								centrifuged, pellet analyzed<br><br>proteinase K phenol/chloroform extraction ethanol precipitation	MGMT promoter	methyl ation	bisulfite treatement<br><br>quantitative fluorogenic real-time PCR TaqMan	at least one gene methylate d: 88%		Clinical: MGMT: 8%	MGMT: 100%	17 primary kidney cancer 9 renal cancer	91 without genitouri nary cancer	Baltimore , Maryland	39.29 9236	- 76.609 383	Hoque et al., 2004	
34	kidney cancer	Kidney Cancer	urine								centrifuged, pellet analyzed<br><br>proteinase K phenol/chloroform extraction ethanol precipitation	RASSF1 A promoter	methyl ation	bisulfite treatement<br><br>quantitative fluorogenic real-time PCR TaqMan	at least one gene methylate d: 88%		Clinical: RASSF1A : 65%	RASSF1 A: 89%	17 primary kidney cancer 9 renal cancer	91 without genitouri nary cancer	Baltimore , Maryland	39.29 9236	- 76.609 383	Hoque et al., 2004	
34	kidney cancer	Kidney Cancer	urine								centrifuged, pellet analyzed<br><br>proteinase K phenol/chloroform extraction ethanol precipitation	GSTP1 promoter	methyl ation	bisulfite treatement<br><br>quantitative fluorogenic real-time PCR TaqMan	at least one gene methylate d: 88%		Clinical: GSTP1: 15%	GSTP1: 100%	17 primary kidney cancer 9 renal cancer	91 without genitouri nary cancer	Baltimore , Maryland	39.29 9236	- 76.609 383	Hoque et al., 2004	
34	kidney cancer	Kidney Cancer	urine								centrifuged, pellet analyzed<br><br>proteinase K phenol/chloroform extraction ethanol precipitation	p16 promoter	methyl ation	bisulfite treatement<br><br>quantitative fluorogenic real-time PCR TaqMan	at least one gene methylate d: 88%		Clinical: p16: 35%	p16: 100%	17 primary kidney cancer 9 renal cancer	91 without genitouri nary cancer	Baltimore , Maryland	39.29 9236	- 76.609 383	Hoque et al., 2004	
34	kidney cancer	Kidney Cancer	urine								centrifuged, pellet analyzed<br><br>proteinase K phenol/chloroform extraction ethanol precipitation	RAR-β2 promoter	methyl ation	bisulfite treatement<br><br>quantitative fluorogenic real-time PCR TaqMan	at least one gene methylate d: 88%		Clinical: RAR-β2: 31%	RAR-β2: 91%	17 primary kidney cancer 9 renal cancer	91 without genitouri nary cancer	Baltimore , Maryland	39.29 9236	- 76.609 383	Hoque et al., 2004	
34	kidney cancer	Kidney Cancer	urine								centrifuged, pellet analyzed<br><br>proteinase K phenol/chloroform extraction ethanol precipitation	ARF promoter	methyl ation	bisulfite treatement<br><br>quantitative fluorogenic real-time PCR TaqMan	at least one gene methylate d: 88%		Clinical: ARF: 31%	ARF: 100%	17 primary kidney cancer 9 renal cancer	91 without genitouri nary cancer	Baltimore , Maryland	39.29 9236	- 76.609 383	Hoque et al., 2004	
34	kidney cancer	Kidney Cancer	urine								centrifuged, pellet analyzed<br><br>proteinase K phenol/chloroform extraction ethanol precipitation	TIMP3 promoter	methyl ation	bisulfite treatement<br><br>quantitative fluorogenic real-time PCR TaqMan	at least one gene methylate d: 88%		Clinical: TIMP3: 46%	TIMP3: 91%	17 primary kidney cancer 9 renal cancer	91 without genitouri nary cancer	Baltimore , Maryland	39.29 9236	- 76.609 383	Hoque et al., 2004	
35	bladder cancer	Bladder Cancer	urine	15 mL			2		0.1333	2 ug DNA	centrifuged, pellet washed in phosphate-buffered saline<br><br>proteinase K phenol/chloroform, ethanol wash	APC, ARF, CDH1, GSTP1, MGMT, CDKN2A , RARβ2, RASSF1	methyl ation	bisulfite treatment Wizard DNA Clean-Up System<br><br>fluorescence-based real-		logistic regression model<br><br>AUC of ROC<br><br>95% CI	69%	at least one methylat ed gene: 10%	160	69	Baltimore , Maryland	39.29 9236	- 76.609 383	Hoque et al., 2006	

												A, and TIMP3 promoter s		time PCR Taq polymerase<br><br>reference gene: ACTB											
3 6	colorecta l cancer	Colorect al Cancer	feces	50 g				10		10 uL	DNA-stabilizing buffer<br><br>Integrity assay using real-time polymerase chain reaction (PCR	VIM and HLTF	methyl ation	bisulfite conversion<br><br>MSP PCR HotStar Polymerase 4% NuSieve 3:1 agarose		t tests chi-square test<br><br>P < 0.05	72.50%	86.90%	125	200	New York, New York	40.73 061	- 73.935 242	Itzkowit z et al., 2007	
3 6	colorecta l cancer	Colorect al Cancer	feces								DNA-stabilizing buffer<br><br>Integrity assay using real-time polymerase chain reaction (PCR	VIM and HLTF	methyl ation	bisulfite conversion<br><br>MSP PCR HotStar Polymerase 4% NuSieve 3:1 agarose		t tests chi-square test<br><br>P < 0.05	72.50%	86.90%	125	200	Evanston , Illinois	42.04 5597	- 87.688 568	Itzkowit z et al., 2007	
3 6	colorecta l cancer	Colorect al Cancer	feces								DNA-stabilizing buffer<br><br>Integrity assay using real-time polymerase chain reaction (PCR	VIM and HLTF	methyl ation	bisulfite conversion<br><br>MSP PCR HotStar Polymerase 4% NuSieve 3:1 agarose		t tests chi-square test<br><br>P < 0.05	72.50%	86.90%	125	200	Ontario, Canada	50	-85	Itzkowit z et al., 2007	
3 6	colorecta l cancer	Colorect al Cancer	feces								DNA-stabilizing buffer<br><br>Integrity assay using real-time polymerase chain reaction (PCR	VIM and HLTF	methyl ation	bisulfite conversion<br><br>MSP PCR HotStar Polymerase 4% NuSieve 3:1 agarose		t tests chi-square test<br><br>P < 0.05	72.50%	86.90%	125	200	Boston, Massach usetts	42.36 1145	- 71.057 083	Itzkowit z et al., 2007	
3 6	colorecta l cancer	Colorect al Cancer	feces								DNA-stabilizing buffer<br><br>Integrity assay using real-time polymerase chain reaction (PCR	VIM and HLTF	methyl ation	bisulfite conversion<br><br>MSP PCR HotStar Polymerase 4% NuSieve 3:1 agarose		t tests chi-square test<br><br>P < 0.05	72.50%	86.90%	125	200	Hines, Illinois	41.85 216	- 87.837	Itzkowit z et al., 2007	
3 6	colorecta l cancer	Colorect al Cancer	feces								DNA-stabilizing buffer<br><br>Integrity assay using real-time polymerase chain reaction (PCR	VIM and HLTF	methyl ation	bisulfite conversion<br><br>MSP PCR HotStar Polymerase 4% NuSieve 3:1 agarose		t tests chi-square test<br><br>P < 0.05	72.50%	86.90%	125	200	Norfolk, Virginia	36.85 0769	- 76.285 873	Itzkowit z et al., 2007	
3 6	colorecta l cancer	Colorect al Cancer	feces								DNA-stabilizing buffer<br><br>Integrity assay using real-time polymerase chain reaction (PCR	VIM and HLTF	methyl ation	bisulfite conversion<br><br>MSP PCR HotStar Polymerase 4% NuSieve 3:1 agarose		t tests chi-square test<br><br>P < 0.05	72.50%	86.90%	125	200	Marlboro ugh, Massach usetts	42.34 5463	- 71.551 628	Itzkowit z et al., 2007	

36	colorectal cancer	Colorectal Cancer	feces								DNA-stabilizing buffer<br><br>Integrity assay using real-time polymerase chain reaction (PCR	VIM and HLTF	methylation	bisulfite conversion<br><br>MSP PCR HotStar Polymerase 4% NuSieve 3:1 agarose		t tests chi-square test<br><br>P < 0.05	72.50%	86.90%	125	200	Cleveland, Ohio	41.505493	-81.68129	Itzkowitz et al., 2007	
37	colorectal cancer	Colorectal Cancer	feces					10		10 µL	DNA-stabilizing buffer<br><br>Integrity assay using real-time polymerase chain reaction (PCR	VIM	methylation	bisulfite conversion<br><br>MSP PCR HotStar Polymerase 4% NuSieve 3:1 agarose		t tests chi-square test<br><br>P < 0.05	hV: 83% hypermethylated vimentin gene (hV) and a two site DNA integrity assay (DY): 86%	hV: 82%<br>DY: 85%<br>hV + DY: 73%	40	122	New York, New York	40.73061	-73.935242	Itzkowitz et al., 2008	
37	colorectal cancer	Colorectal Cancer	feces								DNA-stabilizing buffer<br><br>Integrity assay using real-time polymerase chain reaction (PCR	VIM	methylation	bisulfite conversion<br><br>MSP PCR HotStar Polymerase 4% NuSieve 3:1 agarose		t tests chi-square test<br><br>P < 0.05	hV: 83% hypermethylated vimentin gene (hV) and a two site DNA integrity assay (DY): 86%	hV: 82%<br>DY: 85%<br>hV + DY: 73%	40	122	Evanston, Illinois	42.045597	-87.688568	Itzkowitz et al., 2008	
37	colorectal cancer	Colorectal Cancer	feces								DNA-stabilizing buffer<br><br>Integrity assay using real-time polymerase chain reaction (PCR	VIM	methylation	bisulfite conversion<br><br>MSP PCR HotStar Polymerase 4% NuSieve 3:1 agarose		t tests chi-square test<br><br>P < 0.05	hV: 83% hypermethylated vimentin gene (hV) and a two site DNA integrity assay (DY): 86%	hV: 82%<br>DY: 85%<br>hV + DY: 73%	40	122	Ontario, Canada	50	-85	Itzkowitz et al., 2008	
37	colorectal cancer	Colorectal Cancer	feces								DNA-stabilizing buffer<br><br>Integrity assay using real-time polymerase chain reaction (PCR	VIM	methylation	bisulfite conversion<br><br>MSP PCR HotStar Polymerase 4% NuSieve 3:1 agarose		t tests chi-square test<br><br>P < 0.05	hV: 83% hypermethylated vimentin gene (hV) and a two site DNA integrity assay (DY): 86%	hV: 82%<br>DY: 85%<br>hV + DY: 73%	40	122	Boston, Massachusetts	42.361145	-71.057083	Itzkowitz et al., 2008	
37	colorectal cancer	Colorectal Cancer	feces								DNA-stabilizing buffer<br><br>Integrity assay using real-time polymerase chain reaction (PCR	VIM	methylation	bisulfite conversion<br><br>MSP PCR HotStar Polymerase 4% NuSieve 3:1 agarose		t tests chi-square test<br><br>P < 0.05	hV: 83% hypermethylated vimentin gene (hV) and a two site DNA integrity assay (DY): 86%	hV: 82%<br>DY: 85%<br>hV + DY: 73%	40	122	Hines, Illinois	41.85216	-87.837	Itzkowitz et al., 2008	
37	colorectal cancer	Colorectal Cancer	feces								DNA-stabilizing buffer<br><br>Integrity assay using real-time polymerase chain reaction (PCR	VIM	methylation	bisulfite conversion<br><br>MSP PCR HotStar Polymerase		t tests chi-square test<br><br>P < 0.05	hV: 83% hypermethylated vimentin gene (hV) and a two site DNA	hV: 82%<br>DY: 85%<br>hV + DY: 73%	40	122	Norfolk, Virginia	36.850769	-76.285873	Itzkowitz et al., 2008	

														4% NuSieve<br>3:1 agarose			integrity<br>assay<br>(DY): 86%								
3<br>7	colorecta<br>l cancer	Colorect<br>al<br>Cancer	feces								DNA-stabilizing<br>buffer<br><br>Integrity assay using<br>real-time polymerase<br>chain reaction (PCR	VIM	methyl<br>ation	bisulfite<br>conversion<br><br>MSP PCR<br>HotStar<br>Polymerase<br>4% NuSieve<br>3:1 agarose		t tests<br>chi-square<br>test<br><br>P < 0.05	hV: 83%<br>hypermet<br>hylated<br>vimentin<br>gene (hV)<br>and a two<br>site DNA<br>integrity<br>assay<br>(DY): 86%	hV: 82%<br>DY: 85%<br>hV + DY:<br>73%	40	122	Marlboro<br>ugh,<br>Massach<br>usetts	42.34<br>5463	-<br>71.551<br>628	Itzkowit<br>z et al.,<br>2008	
3<br>7	colorecta<br>l cancer	Colorect<br>al<br>Cancer	feces								DNA-stabilizing<br>buffer<br><br>Integrity assay using<br>real-time polymerase<br>chain reaction (PCR	VIM	methyl<br>ation	bisulfite<br>conversion<br><br>MSP PCR<br>HotStar<br>Polymerase<br>4% NuSieve<br>3:1 agarose		t tests<br>chi-square<br>test<br><br>P < 0.05	hV: 83%<br>hypermet<br>hylated<br>vimentin<br>gene (hV)<br>and a two<br>site DNA<br>integrity<br>assay<br>(DY): 86%	hV: 82%<br>DY: 85%<br>hV + DY:<br>73%	40	122	Clevelan<br>d, Ohio	41.50<br>5493	-<br>81.681<br>29	Itzkowit<br>z et al.,<br>2008	
3<br>8	prostate<br>cancer	Prostate<br>Cancer	urine				0.5			0.5 ug<br>DNA	centrifuged, pellet<br>purified<br>IBI kit<br><br>DNeasy Blood &<br>Tissue Kit	PLA2G1<br>6 CpG<br>adjacent<br>loci	methyl<br>ation	EpiTect Plus<br>FFPE Bisulfite<br>Kit<br><br>one primer-<br>biotinylated<br>PCR<br>streptavidin<br>sepharose<br>beads<br>pyrosequencin<br>g assay<br>2% agarose<br>gel	0.8<br>predictive<br>value<br>1.37 odds<br>ratio	95% CI<br>probability<br>cutoff:<br>0.065	92%	35%	90<br>tumor-<br>associat<br>ed<br>77 non-<br>tumor<br>associat<br>ed	12	Madison,<br>Wisconsi<br>n	43.07<br>3051	-<br>89.401<br>23	Jarrard<br>et al.,<br>2019	
3<br>9	low-risk<br>prostate<br>cancer	Prostate<br>Cancer	urine	5–40<br>ml								GSTP1<br>and APC	methyl<br>ation	internal<br>control: β-<br>Actin<br>external<br>control:<br>targeted<br>plasma DNA<br><br>2-step PCR<br>assay<br>RT–PCR		logistic<br>prediction<br>model<br>logistic<br>regression<br>model<br><br>p-value<br>Student's t<br>test<br><br>area<br>under the<br>receiver<br>operating<br>characteri<br>stic<br>curve<br>(AUC)<br>Wilcoxon–<br>Mann–<br>Whitney<br>test<br>statistic	Gleason<br>score of 7<br>or greater<br>PPV:<br>100%	Gleason<br>score of<br>7 or<br>greater<br>NPV:<br>100%	biopsy<br>cohort:<br>243<br>prostate<br>ctomy<br>cohort:<br>99<br>males	biopsy<br>cohort<br>(no<br>prostate<br>cancer<br>diagnosi<br>s): 430	18<br>clinics:<br>United<br>States	38.88<br>9722	-<br>77.008<br>889	Jatkoe<br>et al.,<br>2015	
4<br>0	bladder<br>cancer	Bladder<br>Cancer	urine								proteinase K<br>digestion protocol	p14ARF,<br>RUNX3,<br>RARβ,<br>DAPK,	methyl<br>ation	methylation-<br>specific<br>polymerase<br>chain reaction	all 3: AUC<br>0.936<br>RUNX3<br>and	chi-square<br>test<br><br>AUC of	all3:<br>98.21%<br>RUNX3<br>and	all3:<br>88.89%<br>RUNX3<br>and	112	10<br>healthy<br>individua<br>ls	Changch<br>un, Jilin,<br>China	43.89<br>1262	125.33<br>1345	K.<br>Wang et<br>al.,<br>2016	

											concentration analysis: NanoDrop	and HPP1		GC buffer<br>PCR Master Mix<br>1% agarose gel	RARβ: AUC 0.918<br>p14ARF and HPP1: AUC 0.688	ROC<br>P < 0.05	RARβ: 96.64%<br>p14ARF and HPP1: 37.50%	RARβ: 88.89%<br>p14ARF and HPP1: 100%		17 glandular cystitis patients					
41	bladder cancer	Bladder Cancer	urine				0.5			0.5 μg DNA		TBX2	methylation	differential methylation hybridization protocol<br><br>llumina<br><br>PCR validation	predictors of progresion:<br>TBX2 predicting progression: area under the curve of 0.96 , p < 0.0001<br>TBX3: p = 0.04<br>GATA2: p = 0.03	p < 0.05<br>Benjamini and Hochberg method<br><br>Kaplan-Meier method<br><br>multivariate Cox regression	TBX2: 100% positive predictive value: 78%	TBX2: 80% negative predictive value: 100%	4		Rotterdam, The Netherlands	51.926517	4.462456	Kandimalla et al., 2012	All biomarkers: TBX2, TBX3, GATA2, and ZIC4 CpG Island
42	bladder cancer	Bladder Cancer	urine	25–100 mL			0.02		0.0003	20 ng DNA	centrifuged, pellet washed in PBS<br><br>DNeasy Tissue kit	OTX1, MEIS1, ONECUT2, SIM2, FOXA1, ZNF503, HOXA9, and OSR1	methylation	bisulfite treatment<br><br>EZ DNA methylation gold kit		logistic regression model<br><br>AUC of ROC<br><br>95% CI	74%	90%	95		Aarhus, Denmark	56.156635	10.210365	Kandimalla et al., 2013	
43	colon cancer	Colorectal Cancer	feces	4 g						32 ug DNA (outlier)	stool stabilization buffer<br><br>stool homogenization buffer<br>centrifuged, pellet submerged in RNAse A<br>sodium acetate & isopropanol and ethanol wash<br>DNA pellet re-suspended in TE & proteinase K<br>digested with SDS incubation, centrifugation<br>phenol/chloroform/isoamylalcohol, incubation, centrifugation<br>DNA precipitated, washed<br>Pellets re-suspended in TE solution	OSMR CpGs	methylation	EZ-96DNA Methylation Kit<br><br>Clean and Concentrator Kit<br><br>Sequencing and Combined Bisulfite Restriction Analysis (COBRA)<br><br>PCR gel, UV SYBR Green Gold		p < 0.01<br>receiver operating characteristic (ROC) curves<br><br>Z values (AUROC0.5)/Std Err (standard error of AUROC)	38%<br><br>Stage I: 11%<br>Stage II: 56%<br>Stage III: 44%<br>Stage IV: 17%	Controls: 5%	69	81	Tilman, Belgium	50.59058	5.57104	Kim et al., 2009	
44	prostate cancer	Prostate Cancer	urine	20 to 600 mL		more than 30 copies/uL DNA in sodium bisulfite					filtered<br>Nucleopore track etched polycarbonate membrane filter<br><br>centrfuge, pellet suspended in lysis/stabilization buffer	GSTP1, APC, RASSF1A, PITX2 and C1orf114p	methylation	bisulfite treatment<br>EZ DNA Methylation-Gold Kit<br><br>droplet digital polymerase chain reaction	prostate specific antigen and urine: AUC 0.95	Welch t-test<br>Spearman rank order correlation<br><br>95% CI	digital rectal examination before: 81%<br>after: 60%		117	12	Herlev, Denmark	55.72876	12.43728	Larsen et al., 2018	Journal of Urology

														ddPCR Supermix<br><br>control: MYOD1											
45	colorectal cancer	Colorectal Cancer	feces	250 mg			2			2 ug DNA	DNA: QIAamp DNA Stool Mini Kit<br><br>RNA: QIAamp Viral Mini Spin Kit	APC, ATM, hMLH1, sFRP2, HLTF, MGMT, and GSTP1<br><br>COX-2 mRNA	methylation<br><br>expression	bisulfite treatment EZ DNA Methylation Kit<br><br>methylation-specific PCR<br><br>positive control: universal methylated DNA<br><br>RT-PCR Reverse Trascriptase		Fisher's exact test Student's t test<br><br>95% CI	colorectal: 75%	90%	20 Colorectal patients	30	Hong Kong, China	22.302711	114.177216	Leung et al., 2007	
45	colorectal cancer	Colorectal Cancer	feces								DNA: QIAamp DNA Stool Mini Kit<br><br>RNA: QIAamp Viral Mini Spin Kit	APC, ATM, hMLH1, sFRP2, HLTF, MGMT, and GSTP1<br><br>COX-2 mRNA	methylation<br><br>expression	bisulfite treatment EZ DNA Methylation Kit<br><br>methylation-specific PCR<br><br>positive control: universal methylated DNA<br><br>RT-PCR Reverse Trascriptase		Fisher's exact test Student's t test<br><br>95% CI	adenomas: 68%	90%	30 paients with polyps	30	Hong Kong, China	22.302711	114.177216	Leung et al., 2007	
46	colorectal cancer	Colorectal Cancer	feces			200 or more copies of b-actin					sDNA stabilization buffer<br><br>target-specific magnetic beads	BMP3 and NDRG4	methylation	enzyme-linked immunosorbent assay plates			98%<br><br>stage I: 95%<br>stage II: 100%<br>stage III: 96%<br>stage IV: 100%<br><br>advanced precancers (AA and SSA)<br>≥1 cm: 57%<br>>2 cm: 73%<br>>3 cm: 83%	90%	93 CRC<br>84 advanced adenomas<br>30 sessile serrated adenoma	155 nonadvanced adenomas<br>641 no colonic lesions	Rochester, Minnesota	44.016369	-92.475395	Lidgard et al., 2013	
46	colorectal cancer	Colorectal Cancer	feces								sDNA stabilization buffer<br><br>target-specific magnetic beads	BMP3 and NDRG4	methylation	enzyme-linked immunosorbent assay plates			98%<br><br>stage I: 95%<br>stage II: 100%<br>stage III: 96%	90%	93 CRC<br>84 advanced adenomas<br>30 sessile	155 nonadvanced adenomas<br>641 no colonic lesions	Scottsdale, Arizona	33.501324	-111.925278	Lidgard et al., 2013	

																	stage IV: 100%<br><br>advanced precancers (AA and SSA) ≥1 cm: 57%<br>>2 cm: 73%<br>>3 cm: 83%		serrated adenoma						
47	bladder cancer	Bladder Cancer	urine				4			4 ug DNA	proteinase K phenol/chloroform	E-cadherin, p16, p14, and RASSF1A	methylation	bisulfite treatment<br><br>Methylation-specific PCR (MSP) assay<br><br>positive control: Lymphocyte DNA methylated with SssI methylase		chi-square test<br>k test<br><br>P < 0.05	83%	100%	57	20	Kaohsiung, Taiwan	22.633333	120.26667	Lin et al., 2010	
48	colorectal cancer and adenomas	Colorectal Cancer	feces					1		1 uL of bisulfite-modified DNA		CNRIP1, FBN1, INA, MAL, and SNCA promoters	methylation	bisulfite treatment<br>TaqMan Universal PCR master mix<br>NoAmpErase UNG<br><br>Reference gene: ALU-C4		Pearson's chi-square test<br>Fisher's exact test<br>Student T-test<br>Mann-Whitney U test<br><br>Receiver Operating Characteristics (ROC) curves					South-East Norway	59.911491	10.757933	Lind et al., 2011	unclear which data is stool-specific
49	colorectal cancer	Colorectal Cancer	feces								Total RNA extraction Qiagen miRNAeasy Mini Kits<br><br>concentration analysis: RiboGreen RNA quantitation kits	miR-21 and miR-106a	expression	Qiagen miRNAeasy Mini Kit<br>SAM-Bead microarray Illumina<br><br>qPCR<br>TaqMan Polymerase or SYBR Green	ΔCt ± SD<br><br>miR-21: colonic adenomas: 7.6 ± 1.6<br>CRC: 6.9 ± 0.5<br>healthy: 6.1 ± 1.6<br><br>miR-106a: colonic adenomas: 0.5 ± 1.6<br>CRC: −0.2 ± 0.5<br>healthy: 0.6 ± 1.6	Student's t tests<br>ANOVA or Kruskall-Wallis<br>Spearman's or Pearson's test<br>logarithmic regression<br><br>P < 0.05			29	8	Dallas, Texas	32.779167	-96.808891	Link et al., 2010	
50	low grade papillary urothelial cell carcinom	Bladder Cancer	urine					2		2 µL of bisulfite-modified DNA	phenol-chloroform extraction protocol	CCND2 promoter	methylation	EpiTect Bisulfite kit<br><br>fluorescence-based real-time PCR	LGPUCC: recurrent: CCND2 53%<br>CCNA1: 58%	cross tabulations and chi-square test<br>Fisher's	UCC cases CCND2: 26%	controls CCND2: 0%	148	56	Baltimore, Maryland	39.299236	-76.609383	Maldonado et al., 2014	All biomarkers: ARF, TIMP3, RAR-β2, NID2,

	a (LGPUCC)													Taq polymerase<br>reference gene: β-actin	NID2: 68% CALCA: 53% nonrecurrent: CCND2: 12% CCNA1: 23.5% NID2: 18% CALCA: 23.5%	exact test Student t-test									CCNA1, AIM1, CALCA and CCND2 promoters
50	low grade papillary urothelial cell carcinoma (LGPUCC)	Bladder Cancer	urine								phenol-chloroform extraction protocol	CCNA1 promoter	methylation	EpiTect Bisulfite kit<br>fluorescence-based real-time PCR Taq polymerase<br>reference gene: β-actin	LGPUCC: recurrent: CCND2 53% CCNA1: 58% NID2: 68% CALCA: 53% nonrecurrent: CCND2: 12% CCNA1: 23.5% NID2: 18% CALCA: 23.5%	cross tabulations and chi-square test Fisher's exact test Student t-test	UCC cases CCNA1: 68%	controls CCNA1: 17%	148	56	Baltimore, Maryland	39.299236	-76.609383	Maldonado et al., 2014	All biomarkers: ARF, TIMP3, RAR-β2, NID2, CCNA1, AIM1, CALCA and CCND2 promoters
50	low grade papillary urothelial cell carcinoma (LGPUCC)	Bladder Cancer	urine								phenol-chloroform extraction protocol	CALCA promoter	methylation	EpiTect Bisulfite kit<br>fluorescence-based real-time PCR Taq polymerase<br>reference gene: β-actin	LGPUCC: recurrent: CCND2 53% CCNA1: 58% NID2: 68% CALCA: 53% nonrecurrent: CCND2: 12% CCNA1: 23.5% NID2: 18% CALCA: 23.5%	cross tabulations and chi-square test Fisher's exact test Student t-test	UCC cases CALCA: 63.5%	controls CALCA: 28.5%	148	56	Baltimore, Maryland	39.299236	-76.609383	Maldonado et al., 2014	All biomarkers: ARF, TIMP3, RAR-β2, NID2, CCNA1, AIM1, CALCA and CCND2 promoters
51	colorectal cancer	Colorectal Cancer	feces				2			2 ug DNA		EN1, SCTR, INHBB CpG Islands	methylation	bisulfite treatment<br>Real-time PCR temperature dissociation (melting curve (MC))<br>Fast Start DNA Master Sybr Green I mix		t-tests or analysis of variance (ANOVA) chi-square or Fisher's-exact test Kaplan – Meier method	44% positive patients 27% overall	97%	30		Barcelona, Spain	41.390205	2.154007	Mayor et al., 2009	
52	bladder cancer	Bladder Cancer	urine	20 mL			0.1		0.0050	at least 100 ng DNA	ZR Urine DNA Isolation Kit<br>validated: Nanodrop	RBBP8	methylation	bisulfite conversion EZ DNA Methylation™ kit		p < 0.05 maximum likelihood estimator<br>non-	RBBP8: 52%	RBBP8: 91%	52	13	Aachen, Germany	50.775555	6.083611	Mijnes et al., 2018	

														positive control: EpiTect® PCR Control DNA Set<br><br>qRT-PCR SYBR Green		parametric Mann-Whitney U test<br>Dunn's multiple comparison test<br>Fisher's exact test<br>Kaplan-Meier method<br>Multivariate Cox regression analysis<br><br>Receiver operating characteristics (ROC)									
53	upper-tract urothelial carcinomas	Bladder Cancer	urine					2		2 µL of bisulphite modified DNA	centrifuged, pellet washed in phosphate-buffered saline<br><br>phenol–chloroform<br><br>validated: Nanodrop	GDF15, TMEFF2 and VIM promoter	methylation	bisulfite modified<br>EZ DNA methylation – Gold kit<br><br>methylation-specific polymerase chain reaction (MSP)<br>TaqMan Universal PCR Master Mix No AmpErase UNG<br><br>positive control: in vitro methylated human DNA		non-parametric Mann–Whitney U-test<br>Mann–Whitney and Kruskall–Wallis tests,<br>Spearman non-parametric correlation test<br>receiver operator characteristics (ROC) curve, area under de curve (AUC)<br>Kaplan–Meier with log rank tes<br>Cox-regression model<br><br>P < 0.05, with Bonferronís correction	91%	100%	22	20	Porto, Portugal	41.150223	-8.629932	Monteiro-Reis et al., 2014	
54	prostate cancer	Prostate Cancer	urine	20-30 mL				3		3 µL of bisulphite	centrifuged, pellet washed in PBS<br><br>phenolchloroform	singleplex-miR-34b/c and miR-	methylation	bisulfite sodium conversion<br>EZ DNA Methylation-		Kruskal-Wallis and Mann-Whitney	singleplex-miR-34b/c and miR-193b:	singleplex-miR-34b/c and miR-	87	32	Porto, Portugal	41.150223	-8.629932	Moreira-Barbosa et al., 2018	

										modified DNA		193b;<br><br>multiplex-APC, GSTP1, and RARβ2 promoters		Gold™ Kit<br><br>singleplex: KAPA SYBR FAST qPCR Kit<br>multiplex: MethyLight assay<br><br>reference gene: β-Actin		non-parametric tests<br>P < 0.05, Bonferroni's correction<br>Spearman non-parametric correlation test<br><br>receiver operator characteristics (ROC) curves, area under the curve (AUC)<br>logistic regression models<br><br>Kaplan-Meier with log rank test<br>Cox-regression model	95.4%<br>PPV: 94.3%<br><br>multiplex-APC, GSTP1, and RARβ2: 94.3%<br>PPV: 94.3%	193b: 84.4%<br>NPV: 87.1%<br><br>multiplex-APC, GSTP1, and RARβ2: 84.4%<br>NPV: 84.4%							
55	renal cell carcinoma (RCC)	Kidney Cancer	urine	2 mL			0.001		0.0005	1 ng DNA	centrifuged, pellet<br><br>Qiagen genomic DNA extraction Kit<br>proteinase K, AE buffer<br><br>concentration analysis<br>Qubit dsDNA High Sensitivity Assay Kit	Differentially Methylated Regions (DMRs)<br>300 DMRs: 150 DMRs with gain in RCC &150 DMRs with loss in RCC	methylation	cfMeDIP–seq protocol<br>KAPA HyperPrep Kit<br>AMPure XP beads<br>MagMeDIP kit<br><br>high-throughput sequencing<br>KAPA HiFi Hotstart ReadyMix<br>NEBNext Multiplex Oligos for Illumina	cell-free DNA: AUROC 0.86						Boston, Massachusetts	42.361145	-71.057083	Nuzzo et al., 2020	
56	aggressive prostate cancer	Prostate Cancer	urine	≤ 50 mL								GSTP1, SFRP2, IGFBP3, IGFBP7, APC, and PTGS2	methylation	quantitaive methylation-specific PCR<br>epiCaPture	area under the curve<br><br>PCa: 0.64<br>high-grade PCa: 0.86<br>high-risk PCa: 0.83<br><br>epiCaPture and PSA: 0.82	P < .05<br>logistic regression models<br><br>area under the receiver operating characteristic curve (AUC)	epiCaPture and PSA: 0.73	epiCaPture and PSA: 0.76	244	209	Atlanta, Georgia	33.753746	-84.38633	O'Reilly et al., 2019	

																decision curve									
56	aggressive prostate cancer	Prostate Cancer	urine									GSTP1, SFRP2, IGFBP3, IGFBP7, APC, and PTGS2	methylation	quantitaive methylation-specific PCR epiCaPture	area under the curve<br><br>PCa: 0.64<br>high-grade PCa: 0.86<br>high-risk PCa: 0.83<br><br>epiCaPture and PSA: 0.82	P < .05<br>logistic regression models<br><br>area under the receiver operating characteristic curve (AUC)<br><br>decision curve	epiCaPture and PSA: 0.73	epiCaPture and PSA: 0.76	244	209	Toronto, Ontario, Canada	43.65107	-79.347015	O'Reilly et al., 2019	
56	aggressive prostate cancer	Prostate Cancer	urine									GSTP1, SFRP2, IGFBP3, IGFBP7, APC, and PTGS2	methylation	quantitaive methylation-specific PCR epiCaPture	area under the curve<br><br>PCa: 0.64<br>high-grade PCa: 0.86<br>high-risk PCa: 0.83<br><br>epiCaPture and PSA: 0.82	P < .05<br>logistic regression models<br><br>area under the receiver operating characteristic curve (AUC)<br><br>decision curve	epiCaPture and PSA: 0.73	epiCaPture and PSA: 0.76	244	209	Dublin, Ireland	53.35014	-6.266155	O'Reilly et al., 2019	
56	aggressive prostate cancer	Prostate Cancer	urine									GSTP1, SFRP2, IGFBP3, IGFBP7, APC, and PTGS2	methylation	quantitaive methylation-specific PCR epiCaPture	area under the curve<br><br>PCa: 0.64<br>high-grade PCa: 0.86<br>high-risk PCa: 0.83<br><br>epiCaPture and PSA: 0.82	P < .05<br>logistic regression models<br><br>area under the receiver operating characteristic curve (AUC)<br><br>decision curve	epiCaPture and PSA: 0.73	epiCaPture and PSA: 0.76	244	209	Norwich, Norfolk, England	52.630886	1.297355	O'Reilly et al., 2019	
57	colorectal cancer	Colorectal Cancer	feces	10 g	0.2 g		0.02			20 ng of bisulfite-modified DNA	washed PBS homogenized, EDTA buffer<br><br>centrifuged, pellet submerged in lysis buffers<br>proteinase K<br>phenol-chloroform-isoamylalcohol<br>sodium acetate, isopropanol<br>ethanol wash, TE buffer<br><br>QIAamp DNA Stool Mini Kit<br><br>concentration	SDC2	methylation	bisulfite conversion EZ DNA Methylation-Gold kit<br><br>purification Zymo-Spin IC column<br><br>2-rounds PCR unidirectional linear target enrichment & quantitative methylation-specific real time PCR (LTE-qMSP)	AUC 0.933	95% CI<br><br>area under the receiver operating characteristic curve (AUC)	90%<br><br>stage I: 83.3%<br>II: 88.2%<br>III: 90.0%<br>IV: 100%	90.9%%	CRC: 50<br>pre-cancerous lesions: 21	healthy individiauls: 22	Seoul, South Korea	37.5326	127.024612	Oh et al., 2017	

											analysis<br>Qubit dsDNA BR assay kit			AptaTaq PCR master mix<br><br>control: COL2A1 region without CpGs											
57	colorectal cancer	Colorectal Cancer	feces								washed PBS<br>homogenized, EDTA buffer<br><br>centrifuged, pellet submerged in lysis buffers<br>proteinase K<br>phenol-chloroform-isoamylalcohol<br>sodium acetate, isopropanol<br>ethanol wash, TE buffer<br><br>QIAamp DNA Stool Mini Kit<br><br>concentration analysis<br>Qubit dsDNA BR assay kit	SDC2	methylation	bisulfite conversion<br>EZ DNA Methylation-Gold kit<br><br>purification<br>Zymo-Spin IC column<br><br>2-rounds PCR unidirectional linear target enrichment & quantitative methylation-specific real time PCR (LTE-qMSP)<br>AptaTaq PCR master mix<br><br>control: COL2A1 region without CpGs	AUC 0.933	95% CI<br><br>area under the receiver operating characteristic curve (AUC)	90%<br><br>stage I: 83.3%<br>II: 88.2%<br>III: 90.0%<br>IV: 100%	90.9%%	CRC: 50<br>pre-cancerous lesions: 21	healthy individiauals: 22	Ilsan, South Korea	38.183333	127.816667	Oh et al., 2017	
58	renal cell carcinoma (RCC)	Kidney Cancer	urine	4 mL				8		8 uL DNA	cohort 2: centrifuged, washed in PBS<br><br>phenol-chloroform	microRNA-30a-5p: two CpGs	methylation	bisulfite conversion<br>EZ DNA Methylation-Gold™ Kit<br><br>cohort 3: SsoAdvanced™ PreAmp Supermix<br><br>quantitative Methylation-specific PCR (qMSP)<br>Xpert Fast SYBR<br>reference gene: β-Actin		receiver operator characteristics (ROC)<br><br>Kaplan Meier curves (with log rank)	testing: 83%<br>validation: 63%	testing: 53%<br>validation: 67%	cohort 2: 53<br>cohort 3: 171	cohort 2: 57<br>cohort 3: 85	Porto, Portugal	41.150223	-8.629932	Outeiro-Pinho et al., 2020	
59	urothelial carcinoma	Bladder Cancer	urine								phenol-chloroform protocol<br><br>concentration analysis<br>NanoDrop	miR-129-2 and miR-663a promoters	methylation	sodium bisulphite conversion<br>EZ DNA Methylation-Gold Kit<br><br>real-time quantitative methylationspecific PCR		receiver operator characteristics curve (ROC)<br>area under the curve (AUC)<br><br>Mann–	87.70%	84%	49	25 prostate cancer<br>25 renal cancer<br>25 healthy individuals	Porto, Portugal	41.150223	-8.629932	Padra˜o et al., 2017	

														KAPA SYBR FAST qPCR Kit<br><br>reference gene: ACTB		Whitney U test<br>Spearman's method, Mann–Whitney or Kruskall–Wallis tests<br>DeLong's test<br>McNemar proportion test<br>Kaplan–Meier with log rank test<br>A Cox regression model<br>P < 0.05, Bonferron´s correction									
60	colorectal cancer	Colorectal Cancer	feces								QIAamp DNA Stool Mini Kit	VIM	methylation	bisulfite conversion EpiTect® Fast DNA Bisulfite Kit<br><br>quantitative methylation-specific PCR<br><br>internal control: ACTB		t-test or ANOVA<br>Fisher's exact test<br><br>P < 0.05	60%<br>PPV: 100%	100%	49	30	Mashhad, Iran	36.310699	59.599457	Pakbaz et al., 2019	
61	systemic lupus erythematosus (SLE)	Systemic Lupus Erythematosus (SLE)	urine								(CFN) cell-free: centrifuge, pellet analyzed<br>RNA: mirVana PARIS kit<br><br>(Exo) pellet containing exosomes: protease inhibitor cocktail<br>RNA: Total exosome RNA and protein isolation kit<br><br>(Sn) exosome-depleted supernatant: unltracentrifuged, pellet analyzed<br>RNA: mirVana PARIS kit<br><br>concentration analysis: NanoDrop	miR-335, miR-302d, miR-200c and miR-146a	microRNA expression	quantitative reverse-transcription PCR<br>TaqMan microRNA assay and Reverse Transcription kit	Exo significantly increased for all miRNAs compared to Sn fraction, especially miR146a (6-fold change, p<0.01)	Student's t-test<br>Fisher's exact test<br>Mann–Whitney U-tes<br><br>AUC of ROC<br><br>P < 0.05			38	12	Valencia, Spain	39.466667	-0.375	Perez-Hernandez et al., 2015	
62	colorectal cancer	Colorectal Cancer	feces							4 mg/mL DNA in RNAse (outlier)	centrifuged, pellet washed in PBS TNE, RNase sodium acetate,	CDKN2A, MGMT, and MLH1	methylation	sodium bisulfite treatment	adenomas:<br>CDKN2A: 31%	t tests<br>chi-square tests<br>Fisher			66		Nashville, Tennessee	36.174465	-86.76796	Petka et al., 2005	

											isopropal alcohol, ethanol wash			Methylation Specific PCR 3% agarose gel, ethidium bromide, UV<br><br>positive controls: DNA from peripheral blood leukocytes, DNA extracted from colorectal cancer cell lines known to be methylated for the three target genes	MGMT: 48%<br>MLH1: 0%<br><br>no polyps:<br>CDKN2A: 16%<br>MGMT: 27%<br>MLH1: 10%	exact tests									
63	bladder cancer	Bladder Cancer	urine				0.005			5 ng of urine DNA	Bladder CARE Urine Collection Kit<br><br>Quick-DNA™ Urine Kit<br><br>Femto™ Human DNA Quantification Kit<br>5 ng DNA or more	3 BC-specific genes<br>2 internal control genes	methylation	bisulfite-FREE<br><br>Bladder CARE	LOD as low as 0.046% "detecting 1 cancer cell for every 2,200 cells analyzed"	Student's two-tailed t tests.<br><br>receiver operating characteristic (ROC) curve area under the curve (AUC)	93.5%<br>PPV: 87.8%	92.6%<br>NPV: 96.2%	77	136	Los Angeles, California	34.052235	-118.243683	Piatti et al., 2021	
64	bladder cancer	Bladder Cancer	urine								centrifuged, pellet in PBS<br><br>QIAamp DNA Mini Kit	p14ARF, p16INK4A, RASSF1A, DAPK, and APC promoters	methylation	bisulfite conversion EpiTect Bisulfite kit<br><br>MSP<br>EpiTect Whole Bisulfitome Kit<br>vs.<br>UroVysion test interphase nuclei, cell-by-cell basis<br>20 target cells, nuclear size or irregular nuclear shape FISH pattern	p14ARF 32%<br>p16INK4a 12%<br>RASSF1A 46%<br>DAPK1 17%<br>APC 46%	Pearson's chi-square test<br>Fisher's exact test<br><br>p value < 0.05	91%		117	8	Bełchatów, Poland	51.36883	19.35671	Pietrusiński et al., 2017	
65	colorectal cancer	Colorectal Cancer	feces								Stool Nucleic Acid Collection and Preservation Tubes<br><br>Stool Total RNA Purification Kit	miR-486-5p	microRNA expression	NEBNext® Multiplex Small RNA Library Prep Set for Illumina<br><br>small-RNA-Seq pipeline analyses	CRC: 223.67 reads<br>control: 43.65 reads	likelihood ratio test (LRT)<br><br>Benjamini–Hochberg (BH)-adjusted p value < 0.05<br><br>Jonckheere–Terpstra test			CRC: 58	healthy individuals: 79	Sassari, Italy	40.725925	8.555683	Pisano et al., 2020	

66	prostate cancer	Prostate Cancer	urine				0.3			300 ng DNA		miR-130a promoter	methylation	EZ DNA Methylation Gold Kit<br><br>quantitative methylation-specific PCR (qMSP)	AUC: 0.89		83.50%	82.30%	101	15	Porto, Portugal	41.150223	-8.629932	Ramalho-Carvalho et al., 2017	
67	bladder cancer	Bladder Cancer	urine				0.5			500 ng of RNA	Puregene DNA Purification Kit<br><br>concentration analysis: OD260/280 ratio	ZNF154, POU4F2, HOXA9, and EOMES	methylation	PCR-based MS-HRM technique		Pearson correlation coefficient<br>Mann–Whitney test<br><br>95% CI	84%	96%	115	59 benign	Aarhus, Denmark	56.156635	10.210365	Reinert et al., 2011	
68	bladder cancer	Bladder Cancer	urine				0.005			5 ng of bisulfite-modified DNA	QIAsymphony Virus/Bacteria Midi kit	EOMES, HOXA9, POU4F2, TWIST1, VIM, and ZNF154	methylation	bisulfite modified<br>EZ-96 DNA methylation D5004<br><br>real-time methylation-specific PCR (MethyLight)<br>TaqMan Universal PCR Master Mix No AmpErase<br><br>positive control: Universal Methylated DNA) with ALU-C4		Wilcoxon-Mann-Whitney test<br>Fisher's exact test<br>chi-square exact test<br>Spearman correlation coefficients<br><br>ROC curve, area under the curve (AUC)<br><br>Kaplan-Meier survival plots<br>Univariate Cox regression analysis<br><br>P < 0.05	validation: 82%-89%<br>recurrence: 88%-94%	validation: 94%-100%<br>recurrence: 43%-67%	184	35	Aarhus, Denmark	56.156635	10.210365	Reinert et al., 2012	
69	bladder cancer	Bladder Cancer	urine	50 mL			1.5		0.0300	up to 1.5 µg of DNA	concentration analysis: Quant-iT PicoGreen dsDNA Quantitation Kit	TWIST1 and NID2	methylation	bisulfite conversion<br><br>MSP<br><br>normalization gene: β-Actin		logistic regression model<br><br>AUC of ROC	90%<br>PPV: 86%	93%<br>NPV: 95%	33	57	Lie`ge, Belgium	50.63373	5.56749	Renard et al., 2009	
70	non-muscle-invasive bladder cancer (NMIBC)	Bladder Cancer	urine				0.05			50 ng DNA	nylon membrane filter<br>wash phosphate-buffered saline<br><br>QiAmp DNA Mini kit<br>AL lysis buffer<br>proteinase K	HS3ST2, SLIT2 and SEPTIN9	methylation	sodium bisulfite treatment<br>EZ DNA Methylation kit<br><br>quantitative multiplex methylation-specific PCR (QMMSP)<br>TaqMan Polymerase	optimism-corrected AUC: 0.96<br><br>follow-up: AUC: 0.84<br><br>threshold algorithm: AUC: 0.82	logistic regression models<br><br>area under the receiver operating characteristic curve (AUC)	97.6%<br><br>follow-up: 90.3%<br><br>threshold algorithm: 94.5%	84.8 %<br>NPV: 99.6%<br><br>follow-up: 65.1%<br>NPV: 97.0%<br><br>threshold algorithm: 75.9%	167	105	Paris, France	48.864716	2.349014	Roperch et al., 2016	

														normalization: ALB with no CpGs				NPV: 98.5%							
71	prostate cancer	Prostate Cancer	urine								ultracentrifugation PBS<br><br>independent set: exosomal RNA purification procedure	urinary extracellular vesicles (uEVs)<br><br>CMTM3 and CDH3 transcripts	particle number<br><br>independent set: expression	Nanoparticle-tracking analysis<br><br>independent set: qRTPCR reference genes: GAPDH and RPL6		Pearson Correlation test<br><br>Student T test Mann Whitney U test	normalization against RPL6: 0.69 p = 0.055<br><br>normalization against GAPDH: 0.65 p = 0.01				Biscay, Spain	43.326702	-2.990057	Royo et al., 2015	
72	bladder cancer	Bladder Cancer	urine	15 mL				1		1 µl extracted DNA	phenol/chloroform ethanol precipitation	BCL2, CDKN2A and NID2	methylation	bisulfite treatment EpiTect® Bisulfite Kit<br><br>nested methylation specific polymerase chain reaction assay HotStart Taq polymerase		Fisher's exact test	80.90%	86.40%	42	21	Holon, Israel	32.01034	34.77918	Scher et al., 2012	Journal of Urology
73	bladder cancer	Bladder Cancer	urine	20-500 mL			2		0.0077	2 ug DNA	centrifuge, pellet washed in phosphate-buffered saline<br><br>QIAamp DNA Mini Kit	APC, RASSF1A and SFRP2	methylation	bisulfite conversion reference gene: ACTB<br><br>MethyLight TaqMan assay reference gene: ALUC4		Fisher's exact test Exact chi-square test Pearson correlation coefficient	52% APC, RASSF1A and SFRP2: 62%	APC, RASSF1A and SFRP2: 100%	113	33 noncancerous urinary lesions	Herlev, Denmark	55.72876	12.43728	Serizawa et al., 2011	All biomarkers: APC, ARF, DBC1, INK4A, RARB, RASSF1A, SFRP1, SFRP2, SFRP4, SFRP5 and WIF1
74	bladder cancer	Bladder Cancer	urine	10 mL			0.005		0.0005	5 ng of total RNA	centrifuge, pellet analyzed<br><br>phenol-chloroform	miR-137, miR-124-2, miR-124-3, and miR-9-3	methylation	Bisulfite conversion methylation-specific PCR (MSP) bisulfite sequencing, pyrosequencing<br><br>Reverse transcription Quantitative real-time polymerase chain TaqMan Polymerase	AUC: 0.916	Student t test one-way analysis of variance (ANOVA), post hoc Tukey test Fisher exact test Pearson correlation coefficient<br><br>Receiver operating characteristic (ROC) curve p < 0.05	81%	89%	86	20	Hokkaido, Japan	43.066666	141.350006	Shimizu et al., 2013	
75	colorectal cancer	Colorectal Cancer	feces				0.5			500 ng of bisulfite-	microbiota: GNOME DNA Isolation Kit	16s rRNA	microbiota sequen	microbiota: PCR Illumina		Pearson's chi-square test	cohort 1: 29.2%	cohort 1: 89.7%	cohort 1: 226 cohort 2:	cohort 2: 500 without	Paris, France	48.864716	2.349014	Sobhani et al., 2019	

										converted DNA	human:<br>QiAamp DNA stool Mini Kit	NPY, Wif1, and NPY	cing<br><br>human methylation	sequencing<br><br>human:<br>bisulfite conversion<br>EZ DNA Methylation kit<br><br>Illumina Golden Gate methylation bead arrays quantitative, single-gene methylation-specific PCR (QS-MSP) quantitative multiplex methylation-specific PCR (QMMSP)<br><br>reference gene: ALB without CpGs		Student's t test<br><br>logistic regression model<br>P < 0.05			500 digestive symptoms	digestive symptoms	Créteil, France				
76	non–muscle-invasive bladder cancer (NMIBC)	Bladder Cancer	urine	40 mL			0.5		0.0125	≤ 500 ng	centrifuged, pellet washed in phosphate-buffered saline<br><br>phenol/chloroform/isoamyl alcohol<br><br>concentration analysis: NanoDrop	DLX1, ITGA4, SOX17, ASTN1, RXFP3, and ZNF671	methylation	bisulfite treatment EpiTect Fast DNA Bisulfite Kit<br><br>quantitative methylation-specific PCR (qMSP) GynTect		Fisher exact test Cohen's κ<br><br>95% CI	60%	96.70%	40	30	Jena, Germany	50.927223	11.586111	Steinbach et al., 2020	
77	bladder cancer	Bladder Cancer	urine	50 mL							centridufuged, pellet analyzed	SOX1, IRAK3, andL1-MET	methylation	bisulfite conversion EZ DNA Methylation Kit<br><br>PCR biotin-labeled primers<br><br>pyrosequencing		logistic regression models<br><br>area under the receiver operating characteristic curve (AUC)<br><br>95% CI	SOX1, IRAK3, andL1-MET 3-gene panel: 86%	SOX1, IRAK3, andL1-MET 3-gene panel: 89%	20	20	Los Angeles, California	34.052235	-118.243683	Su et al., 2014	All biomarkers: HOXA9, SOX1, NPY, IRAK3, and ZO2, and hypomethylation of L1-MET
78	bladder cancer	Bladder Cancer	urine				10			10 ug DNA	proteinase K/organic extraction method	SFRP1	methylation	bisulfite conversion<br><br>real-time PCR		Fisher's exact test<br><br>AUC of ROC<br><br>95% CI	SFRP1: 36.65%	SFRP1: 93.3%	82	15	Shanghai, China	31.224361	121.46917	Sun et al., 2009	
79	colorectal cancer	Colorectal Cancer	fecal occult blood								samples mixed in buffer solution<br><br>hemoglobin > 0.2 μg/ml	SEPT9	methylation	Immune colloidal gold technique		ANOVA Spearman's rank correlations t tests<br><br>area under the	CRC patients: 73.0%% PPV: 63.0%<br><br>intestinal polyps and	CRC patients: 94.5% NPV: 96.5%<br><br>intestinal polyps and	650		Tianjin , China	39.133331	117.183334	Sun et al., 2019	

																curve (AUC) of the receiver operating characteristic curve (ROC)<br><br>Fisher exact test<br>Logistic regression analysis<br>Chi-square tests<br><br>P < 0.05	adenoma: 17.1%<br>PPV: 34.1%	adenoma: 94.5%<br>NPV: 87.3%							
80	non–muscle-invasive bladder cancer (NMIBC)	Bladder Cancer	urine	12 mL							Cytolyt fixation liquid centrifuged, pellet analyzed<br><br>Bladder EpiCheck DNA extraction kit	15 (proprietary)	methylation	quantitative real-time polymerase chain reaction (qRT-PCR)<br>Bladder EpiCheck test		Pearson chi-square test<br><br>AUC of ROC<br>z test<br><br>P <0.05	62.3%<br>low-grade tumors: 46.1%<br>high-grade tumors: 83.3%<br><br>cytology and EpiCheck: 66.7%<br>low-grade tumors: 56.4%<br>high-grade tumors: 90%<br><br>PPV: 68.2%<br>cytology and EpiCheck: 68.6%	86.3%<br><br>cytology and EpiCheck: 85.6%<br><br>NPV: 82.9%<br>cytology and EpiCheck: 84.5%	243		Bolzano, Italy	46.5	11.35	Trenti et al., 2019	
81	bladder cancer	Bladder Cancer	urine				1			1 ug Total RNA	centrifuged, pellet purified<br>QIAamp DNA Mini Kit	sFRP-1	methylation	bisulfite treatment<br><br>methylation-specific PCR<br>2% agarose gel, UV<br><br>bisulfite sequencing<br><br>internal control: GADPH	AUC:<br>sFRP-1: 0.683<br>sFRP-2: 0.611<br>sFRP-4: 0.614<br>sFRP-5: 0.620<br>Dkk-3: 0.684<br>Wif-1: 0.669	logistic regression models<br><br>area under the receiver operator characteristics (ROC) curve (AUC)<br><br>Mann-Whitney test<br>Kruskal-Wallis test<br>chi-square test	sFRP-1: 75.9%	sFRP-1: 53.7%	24	20	San Francisco, California	37.773972	-122.431297	Urakami et al., 2006	

																Fisher’s exact test<br><br>P < 0.05										
81	bladder cancer	Bladder Cancer	urine								centrifuged, pellet purified QIAamp DNA Mini Kit	sFRP-1	methylation	bisulfite treatment<br><br>methylation-specific PCR 2% agarose gel, UV<br><br>bisulfite sequencing<br><br>internal control: GADPH	AUC: sFRP-1: 0.683 sFRP-2: 0.611 sFRP-4: 0.614 sFRP-5: 0.620 Dkk-3: 0.684 Wif-1: 0.669	logistic regression models<br><br>area under the receiver operator characteristics (ROC) curve (AUC)<br><br>Mann-Whitney test Kruskal-Wallis test chi-square test Fisher’s exact test<br><br>P < 0.05	sFRP-1: 75.9%	sFRP-1: 53.7%	24	20	Izumo, Japan	35.36667	132.76667	Urakami et al., 2006	
81	bladder cancer	Bladder Cancer	urine								centrifuged, pellet purified QIAamp DNA Mini Kit	sFRP-1	methylation	bisulfite treatment<br><br>methylation-specific PCR 2% agarose gel, UV<br><br>bisulfite sequencing<br><br>internal control: GADPH	AUC: sFRP-1: 0.683 sFRP-2: 0.611 sFRP-4: 0.614 sFRP-5: 0.620 Dkk-3: 0.684 Wif-1: 0.669	logistic regression models<br><br>area under the receiver operator characteristics (ROC) curve (AUC)<br><br>Mann-Whitney test Kruskal-Wallis test chi-square test Fisher’s exact test<br><br>P < 0.05	sFRP-1: 75.9%	sFRP-1: 53.7%	24	20	Kagoshima, Japan	30.732412	30.732412	Urakami et al., 2006	
81	bladder cancer	Bladder Cancer	urine								centrifuged, pellet purified QIAamp DNA Mini Kit	sFRP-2	methylation	bisulfite treatment<br><br>methylation-specific PCR 2% agarose gel, UV<br><br>bisulfite sequencing<br><br>internal	AUC: sFRP-1: 0.683 sFRP-2: 0.611 sFRP-4: 0.614 sFRP-5: 0.620 Dkk-3: 0.684	logistic regression models<br><br>area under the receiver operator characteristics (ROC) curve	sFRP-2: 63.0%	sFRP-2: 57.4%	24	20	San Francisco, California	37.773972	-122.431297	Urakami et al., 2006	

														control: GADPH	Wif-1: 0.669	(AUC)<br><br>Mann-Whitney test<br>Kruskal-Wallis test<br>chi-square test<br>Fisher’s exact test<br><br>P < 0.05									
81	bladder cancer	Bladder Cancer	urine								centrifuged, pellet purified<br>QIAamp DNA Mini Kit	sFRP-2	methylation	bisulfite treatment<br><br>methylation-specific PCR<br>2% agarose gel, UV<br><br>bisulfite sequencing<br><br>internal control: GADPH	AUC:<br>sFRP-1: 0.683<br>sFRP-2: 0.611<br>sFRP-4: 0.614<br>sFRP-5: 0.620<br>Dkk-3: 0.684<br>Wif-1: 0.669	logistic regression models<br><br>area under the receiver operator characteristics (ROC) curve (AUC)<br><br>Mann-Whitney test<br>Kruskal-Wallis test<br>chi-square test<br>Fisher’s exact test<br><br>P < 0.05	sFRP-2: 63.0%	sFRP-2: 57.4%	24	20	Izumo, Japan	35.36667	132.76667	Urakami et al., 2006	
81	bladder cancer	Bladder Cancer	urine								centrifuged, pellet purified<br>QIAamp DNA Mini Kit	sFRP-2	methylation	bisulfite treatment<br><br>methylation-specific PCR<br>2% agarose gel, UV<br><br>bisulfite sequencing<br><br>internal control: GADPH	AUC:<br>sFRP-1: 0.683<br>sFRP-2: 0.611<br>sFRP-4: 0.614<br>sFRP-5: 0.620<br>Dkk-3: 0.684<br>Wif-1: 0.669	logistic regression models<br><br>area under the receiver operator characteristics (ROC) curve (AUC)<br><br>Mann-Whitney test<br>Kruskal-Wallis test<br>chi-square test<br>Fisher’s exact test<br><br>P < 0.05	sFRP-2: 63.0%	sFRP-2: 57.4%	24	20	Kagoshima, Japan	30.732412	30.732412	Urakami et al., 2006	
81	bladder cancer	Bladder Cancer	urine								centrifuged, pellet purified	sFRP-4	methylation	bisulfite treatment	AUC:<br>sFRP-1: 0.683	logistic regression models	sFRP-4: 38.9%	sFRP-4: 87.0%	24	20	San Francisc	37.773972	-122.431297	Urakami et al., 2006	

											QIAamp DNA Mini Kit			methylation-specific PCR<br>2% agarose gel, UV<br><br>bisulfite sequencing<br><br>internal control: GADPH	sFRP-2: 0.611<br>sFRP-4: 0.614<br>sFRP-5: 0.620<br>Dkk-3: 0.684<br>Wif-1: 0.669	area under the receiver operator characteristics (ROC) curve (AUC)<br><br>Mann-Whitney test<br>Kruskal-Wallis test<br>chi-square test<br>Fisher's exact test<br><br>P < 0.05					o, California				
81	bladder cancer	Bladder Cancer	urine								centrifuged, pellet purified<br>QIAamp DNA Mini Kit	sFRP-4	methylation	bisulfite treatment<br><br>methylation-specific PCR<br>2% agarose gel, UV<br><br>bisulfite sequencing<br><br>internal control: GADPH	AUC:<br>sFRP-1: 0.683<br>sFRP-2: 0.611<br>sFRP-4: 0.614<br>sFRP-5: 0.620<br>Dkk-3: 0.684<br>Wif-1: 0.669	logistic regression models<br><br>area under the receiver operator characteristics (ROC) curve (AUC)<br><br>Mann-Whitney test<br>Kruskal-Wallis test<br>chi-square test<br>Fisher's exact test<br><br>P < 0.05	sFRP-4: 38.9%	sFRP-4: 87.0%	24	20	Izumo, Japan	35.36667	132.76667	Urakami et al., 2006	
81	bladder cancer	Bladder Cancer	urine								centrifuged, pellet purified<br>QIAamp DNA Mini Kit	sFRP-4	methylation	bisulfite treatment<br><br>methylation-specific PCR<br>2% agarose gel, UV<br><br>bisulfite sequencing<br><br>internal control: GADPH	AUC:<br>sFRP-1: 0.683<br>sFRP-2: 0.611<br>sFRP-4: 0.614<br>sFRP-5: 0.620<br>Dkk-3: 0.684<br>Wif-1: 0.669	logistic regression models<br><br>area under the receiver operator characteristics (ROC) curve (AUC)<br><br>Mann-Whitney test<br>Kruskal-Wallis test<br>chi-square	sFRP-4: 38.9%	sFRP-4: 87.0%	24	20	Kagoshima, Japan	30.732412	30.732412	Urakami et al., 2006	

																test<br>Fisher's exact test<br><br>P < 0.05									
81	bladder cancer	Bladder Cancer	urine								centrifuged, pellet purified<br>QIAamp DNA Mini Kit	sFRP-5	methylation	bisulfite treatment<br><br>methylation-specific PCR<br>2% agarose gel, UV<br><br>bisulfite sequencing<br><br>internal control: GADPH	AUC:<br>sFRP-1: 0.683<br>sFRP-2: 0.611<br>sFRP-4: 0.614<br>sFRP-5: 0.620<br>Dkk-3: 0.684<br>Wif-1: 0.669	logistic regression models<br><br>area under the receiver operator characteristics (ROC) curve (AUC)<br><br>Mann-Whitney test<br>Kruskal-Wallis test<br>chi-square test<br>Fisher's exact test<br><br>P < 0.05	sFRP-5: 87.0%	sFRP-5: 37.0%	24	20	San Francisco, California	37.773972	-122.431297	Urakami et al., 2006	
81	bladder cancer	Bladder Cancer	urine								centrifuged, pellet purified<br>QIAamp DNA Mini Kit	sFRP-5	methylation	bisulfite treatment<br><br>methylation-specific PCR<br>2% agarose gel, UV<br><br>bisulfite sequencing<br><br>internal control: GADPH	AUC:<br>sFRP-1: 0.683<br>sFRP-2: 0.611<br>sFRP-4: 0.614<br>sFRP-5: 0.620<br>Dkk-3: 0.684<br>Wif-1: 0.669	logistic regression models<br><br>area under the receiver operator characteristics (ROC) curve (AUC)<br><br>Mann-Whitney test<br>Kruskal-Wallis test<br>chi-square test<br>Fisher's exact test<br><br>P < 0.05	sFRP-5: 87.0%	sFRP-5: 37.0%	24	20	Izumo, Japan	35.36667	132.76667	Urakami et al., 2006	
81	bladder cancer	Bladder Cancer	urine								centrifuged, pellet purified<br>QIAamp DNA Mini Kit	sFRP-5	methylation	bisulfite treatment<br><br>methylation-specific PCR<br>2% agarose gel, UV<br><br>bisulfite sequencing	AUC:<br>sFRP-1: 0.683<br>sFRP-2: 0.611<br>sFRP-4: 0.614<br>sFRP-5: 0.620<br>Dkk-3: 0.684	logistic regression models<br><br>area under the receiver operator characteristics (ROC)	sFRP-5: 87.0%	sFRP-5: 37.0%	24	20	Kagoshima, Japan	30.732412	30.732412	Urakami et al., 2006	

														internal<br>control:<br>GADPH	Wif-1:<br>0.669	curve<br>(AUC)<br><br>Mann-<br>Whitney<br>test<br>Kruskal-<br>Wallis test<br>chi-square<br>test<br>Fisher's<br>exact test<br><br>P < 0.05									
8<br>1	bladder<br>cancer	Bladder<br>Cancer	urine								centrifuged, pellet<br>purified<br>QIAamp DNA Mini<br>Kit	Wif-1	methyl<br>ation	bisulfite<br>treatment<br><br>methylation-<br>specific PCR<br>2% agarose<br>gel, UV<br><br>bisulfite<br>sequencing<br><br>internal<br>control:<br>GADPH	AUC:<br>sFRP-1:<br>0.683<br>sFRP-2:<br>0.611<br>sFRP-4:<br>0.614<br>sFRP-5:<br>0.620<br>Dkk-3:<br>0.684<br>Wif-1:<br>0.669	logistic<br>regression<br>models<br><br>area<br>under the<br>receiver<br>operator<br>characteri<br>stics<br>(ROC)<br>curve<br>(AUC)<br><br>Mann-<br>Whitney<br>test<br>Kruskal-<br>Wallis test<br>chi-square<br>test<br>Fisher's<br>exact test<br><br>P < 0.05	Wif-1:<br>63.0%	Wif-1:<br>72.2%	24	20	San<br>Francisc<br>o,<br>California	37.77<br>3972	-<br>122.43<br>1297	Urakami<br>et al.,<br>2006	
8<br>1	bladder<br>cancer	Bladder<br>Cancer	urine								centrifuged, pellet<br>purified<br>QIAamp DNA Mini<br>Kit	Wif-1	methyl<br>ation	bisulfite<br>treatment<br><br>methylation-<br>specific PCR<br>2% agarose<br>gel, UV<br><br>bisulfite<br>sequencing<br><br>internal<br>control:<br>GADPH	AUC:<br>sFRP-1:<br>0.683<br>sFRP-2:<br>0.611<br>sFRP-4:<br>0.614<br>sFRP-5:<br>0.620<br>Dkk-3:<br>0.684<br>Wif-1:<br>0.669	logistic<br>regression<br>models<br><br>area<br>under the<br>receiver<br>operator<br>characteri<br>stics<br>(ROC)<br>curve<br>(AUC)<br><br>Mann-<br>Whitney<br>test<br>Kruskal-<br>Wallis test<br>chi-square<br>test<br>Fisher's<br>exact test<br><br>P < 0.05	Wif-1:<br>63.0%	Wif-1:<br>72.2%	24	20	Izumo,<br>Japan	35.36<br>667	132.76<br>667	Urakami<br>et al.,<br>2006	

81	bladder cancer	Bladder Cancer	urine								centrifuged, pellet purified<br>QIAamp DNA Mini Kit	Wif-1	methylation	bisulfite treatment<br><br>methylation-specific PCR<br>2% agarose gel, UV<br><br>bisulfite sequencing<br><br>internal control: GADPH	AUC:<br>sFRP-1: 0.683<br>sFRP-2: 0.611<br>sFRP-4: 0.614<br>sFRP-5: 0.620<br>Dkk-3: 0.684<br>Wif-1: 0.669	logistic regression models<br><br>area under the receiver operator characteristics (ROC) curve (AUC)<br><br>Mann-Whitney test<br>Kruskal-Wallis test<br>chi-square test<br>Fisher's exact test<br><br>P < 0.05	Wif-1: 63.0%	Wif-1: 72.2%	24	20	Kagoshima, Japan	30.732412	30.732412	Urakami et al., 2006	
81	bladder cancer	Bladder Cancer	urine								centrifuged, pellet purified<br>QIAamp DNA Mini Kit	Dkk-3	methylation	bisulfite treatment<br><br>methylation-specific PCR<br>2% agarose gel, UV<br><br>bisulfite sequencing<br><br>internal control: GADPH	AUC:<br>sFRP-1: 0.683<br>sFRP-2: 0.611<br>sFRP-4: 0.614<br>sFRP-5: 0.620<br>Dkk-3: 0.684<br>Wif-1: 0.669	logistic regression models<br><br>area under the receiver operator characteristics (ROC) curve (AUC)<br><br>Mann-Whitney test<br>Kruskal-Wallis test<br>chi-square test<br>Fisher's exact test<br><br>P < 0.05	Dkk-3: 38.9%	Dkk-3: 92.6%	24	20	San Francisco, California	37.773972	-122.431297	Urakami et al., 2006	
81	bladder cancer	Bladder Cancer	urine								centrifuged, pellet purified<br>QIAamp DNA Mini Kit	Dkk-3	methylation	bisulfite treatment<br><br>methylation-specific PCR<br>2% agarose gel, UV<br><br>bisulfite sequencing<br><br>internal control: GADPH	AUC:<br>sFRP-1: 0.683<br>sFRP-2: 0.611<br>sFRP-4: 0.614<br>sFRP-5: 0.620<br>Dkk-3: 0.684<br>Wif-1: 0.669	logistic regression models<br><br>area under the receiver operator characteristics (ROC) curve (AUC)<br><br>Mann-Whitney test	Dkk-3: 38.9%	Dkk-3: 92.6%	24	20	Izumo, Japan	35.366667	132.766667	Urakami et al., 2006	

																Kruskal-Wallis test<br>chi-square test<br>Fisher's exact test<br><br>P < 0.05									
81	bladder cancer	Bladder Cancer	urine								centrifuged, pellet purified<br>QIAamp DNA Mini Kit	Dkk-3	methylation	bisulfite treatment<br><br>methylation-specific PCR<br>2% agarose gel, UV<br><br>bisulfite sequencing<br><br>internal control: GADPH	AUC:<br>sFRP-1: 0.683<br>sFRP-2: 0.611<br>sFRP-4: 0.614<br>sFRP-5: 0.620<br>Dkk-3: 0.684<br>Wif-1: 0.669	logistic regression models<br><br>area under the receiver operator characteristics (ROC) curve (AUC)<br><br>Mann-Whitney test<br>Kruskal-Wallis test<br>chi-square test<br>Fisher's exact test<br><br>P < 0.05	Dkk-3: 38.9%	Dkk-3: 92.6%	24	20	Kagoshima, Japan	30.732412	30.732412	Urakami et al., 2006	
82	endometrial cancer	Endometrial Cancer	urine		15 mL		0.05		0.0033	50 ng modified DNA	DNA mini and blood mini kit<br><br>concentration analysis: NanoDrop	GHSR, SST, and ZIC1	methylation	bisulfte conversion<br>EZ DNA Methylation Kit<br><br>quantitative methylation-specifc PCR (qMSP)<br><br>reference gene: ACTB	AUC<br><br>full void urine: AUC 0.86 to 0.95<br><br>GHSR: 0.95<br>SST: 0.92<br>ZIC1: 0.86	Spearman's rank correlation<br><br>Mann–Whitney U test<br><br>receiver operating characteristic (ROC) area under the curve (AUC)			42	46	Amsterdam, The Netherlands	52.377956	4.89707	van den Helder et al., 2020	
83	bladder cancer	Bladder Cancer	urine				1			1 ug DNA	centrifuged, pellet analyzed<br><br>QIAamp DNA Mini Kit<br><br>concentration analysis: NanoDrop	CFTR, SALL3, and TWIST1	methylation	bisulfite modification<br>EpiTect Bisulfite kit<br><br>Ampli Taq Gold 360 DNA polymerase<br>2% agarose gel, ethidium bromide<br><br>pyrosequencing<br>biotin-labeled	methylation and cytology: CFTR, SALL3, and TWIST1: AUC 0.86	logistic regression models<br><br>area under the receiver operator characteristics (ROC) curve (AUC)<br><br>p < 0.1	methylation and cytology: CFTR, SALL3, and TWIST1: 96%<br>PPV: 56%	methylation and cytology: CFTR, SALL3, and TWIST1: 40%,<br>NPV: 92%	111	57	Barcelona, Spain	41.390205	2.154007	van der Heijden et al., 2018	All biomarkers: CDH13, CFTR, NID2, SALL3, TMEFF2, TWIST1, and VIM2
83	bladder cancer	Bladder Cancer	urine								centrifuged, pellet analyzed	CFTR, SALL3,	methylation	bisulfite modification<br>EpiTect	methylation and cytology:	logistic regression models	methylation and cytology:	methylation and cytology:	111	57	Nijmegen, The	51.8425	5.85278	van der Heijde	All biomarkers: CDH13,

											QIAamp DNA Mini Kit<br><br>concentration analysis: NanoDrop	and TWIST1		Bisulfite kit<br><br>Ampli Taq Gold 360 DNA polymerase 2% agarose gel, ethidium bromide<br><br>pyrosequencing biotin-labeled	CFTR, SALL3, and TWIST1: AUC 0.86	area under the receiver operator characteristics (ROC) curve (AUC)<br><br>p < 0.1	CFTR, SALL3, and TWIST1: 96% PPV: 56%	CFTR, SALL3, and TWIST1: 40%, NPV: 92%			Netherlands			n et al., 2018	CFTR, NID2, SALL3, TMEFF2, TWIST1, and VIM2
83	bladder cancer	Bladder Cancer	urine								centrifuged, pellet analyzed<br><br>QIAamp DNA Mini Kit<br><br>concentration analysis: NanoDrop	CFTR, SALL3, and TWIST1	methylation	bisulfite modification EpiTect Bisulfite kit<br><br>Ampli Taq Gold 360 DNA polymerase 2% agarose gel, ethidium bromide<br><br>pyrosequencing biotin-labeled	methylation and cytology: CFTR, SALL3, and TWIST1: AUC 0.86	logistic regression models<br><br>area under the receiver operator characteristics (ROC) curve (AUC)<br><br>p < 0.1	methylation and cytology: CFTR, SALL3, and TWIST1: 96% PPV: 56%	methylation and cytology: CFTR, SALL3, and TWIST1: 40%, NPV: 92%	111	57	Bucharest, Romania	44.389122	26.115875	van der Heijden et al., 2018	All biomarkers: CDH13, CFTR, NID2, SALL3, TMEFF2, TWIST1, and VIM2
83	bladder cancer	Bladder Cancer	urine								centrifuged, pellet analyzed<br><br>QIAamp DNA Mini Kit<br><br>concentration analysis: NanoDrop	CFTR, SALL3, and TWIST1	methylation	bisulfite modification EpiTect Bisulfite kit<br><br>Ampli Taq Gold 360 DNA polymerase 2% agarose gel, ethidium bromide<br><br>pyrosequencing biotin-labeled	methylation and cytology: CFTR, SALL3, and TWIST1: AUC 0.86	logistic regression models<br><br>area under the receiver operator characteristics (ROC) curve (AUC)<br><br>p < 0.1	methylation and cytology: CFTR, SALL3, and TWIST1: 96% PPV: 56%	methylation and cytology: CFTR, SALL3, and TWIST1: 40%, NPV: 92%	111	57	Houston, Texas	29.749907	-95.358421	van der Heijden et al., 2018	All biomarkers: CDH13, CFTR, NID2, SALL3, TMEFF2, TWIST1, and VIM2
84	hematuria	Hematuria	urine				0.1			100 ng DNA	QIAamp DNA Micro Kit<br><br>concentration analysis: Quant-iT PicoGreen dsDNA Quantification Kit	TWIST1, ONECUT2,and OTX1 methylation<br><br>FGFR3, TERT and HRAS mutation	mutation<br><br>methylation	bisulfite treatment EZ DNA Methylation-Lightning Kit<br><br>TWIST1: Methylation specific polymerase chain reaction Quantitative PCR mix<br><br>OTX1 and ONECUT2: SNaPshot™	TWIST1, ONECUT2,and OTX1 methylation & FGFR3, TERT and HRAS mutation panel: AUC 0.93 optimism corrected AUC 0.92.	95% CI	TWIST1, ONECUT2,and OTX1 methylation & FGFR3, TERT and HRAS mutation panel: 97%	TWIST1, ONECUT2,and OTX1 methylation & FGFR3, TERT and HRAS mutation panel: 83% NOV: 99.6-99.9%	74: bladder cancer	80 without bladder cancer	Rotterdam, The Netherlands	51.926517	4.462456	van Kessel et al., 2016	All biomarkers: TERT, PIK3CA, FGFR3, HRAS, KRAS and NRAS mutations OTX1, ONECUT2 and TWIST1 methylation
85	hematuria	Hematuria	urine								QIAmp DNA Mini-Kit	FGFR3, TERT and HRAS mutation<br><br>OTX1,	mutation<br><br>methylation	bisulfite treatment EZ DNA Methylation-Gold<br><br>QuantiTect	AUC 0.96 optimism corrected AUC 0.95	logistic regression models Mann-Whitney U test Fisher	93%	86% NPV: 99%	97	103	Rotterdam, The Netherlands	51.926517	4.462456	van Kessel et al., 2017	

												ONECUT 2 and TWIST1 methylation		Multiplex PCR Master Mix		exact test<br><br>area under the receiver operator characteristics (ROC) curve (AUC)<br><br>95% CI									
85	hematuria	Hematuria	urine								QIAmp DNA Mini-Kit	FGFR3, TERT and HRAS mutation<br><br>OTX1, ONECUT 2 and TWIST1 methylation	mutation<br><br>methylation	bisulfite treatment EZ DNA Methylation-Gold<br><br>QuantiTect Multiplex PCR Master Mix	AUC 0.96 optimism corrected AUC 0.95	logistic regression models Mann-Whitney U test Fisher exact test<br><br>area under the receiver operator characteristics (ROC) curve (AUC)<br><br>95% CI	93%	86% NPV: 99%	97	103	Barcelona, Spain	41.390205	2.154007	van Kessel et al., 2017	
85	hematuria	Hematuria	urine								QIAmp DNA Mini-Kit	FGFR3, TERT and HRAS mutation<br><br>OTX1, ONECUT 2 and TWIST1 methylation	mutation<br><br>methylation	bisulfite treatment EZ DNA Methylation-Gold<br><br>QuantiTect Multiplex PCR Master Mix	AUC 0.96 optimism corrected AUC 0.95	logistic regression models Mann-Whitney U test Fisher exact test<br><br>area under the receiver operator characteristics (ROC) curve (AUC)<br><br>95% CI	93%	86% NPV: 99%	97	103	Madrid, Spain	40.416775	-3.70379	van Kessel et al., 2017	
85	hematuria	Hematuria	urine								QIAmp DNA Mini-Kit	FGFR3, TERT and HRAS mutation<br><br>OTX1, ONECUT 2 and TWIST1 methylation	mutation<br><br>methylation	bisulfite treatment EZ DNA Methylation-Gold<br><br>QuantiTect Multiplex PCR Master Mix	AUC 0.96 optimism corrected AUC 0.95	logistic regression models Mann-Whitney U test Fisher exact test<br><br>area under the receiver operator	93%	86% NPV: 99%	97	103	Uppsala, Sweden	59.853642	17.634966	van Kessel et al., 2017	

																characteristics (ROC) curve (AUC)<br><br>95% CI									
86	colorectal cancer	Colorectal Cancer	feces					2		2-5 µL bisulfite-converted DNA	methylation: QIAamp DNA Stool Mini Kit<br><br>expression: RNeasy Mini Kit	GRIA4 and VIPR2<br><br>GRIA4, VIPR2, SPOCK1 and SLC6A3 mRNA and proteins	methylation<br><br>expression	bisulfite conversion EZ DNA Methylation Gold Kit TM<br><br>MethyLight qPCR TaqMan Genotyping Master mix positive control: Human Methylated & Non-Methylated (WGA) DNA Set reference gene: Alu digital droplet PCR ddPCR Supermix<br><br>expression: mRNA: SsoAdvanced Universal SYBR® Green Supermix protein: Western blot		Welch's t-test	90%		10		Cagliari, Italy	39.227779	9.111111	Vega-Benedetti et al., 2020	
87	prostate cancer	Prostate Cancer	urine	20 – 120 mL	50 uL			5		5 uL modifed DNA	centrifuge, pellet in PBS<br><br>DNA extraction with Puregene Kit<br><br>DNA quantification: Picogreen DNAQuantification Assay	GSTP1, RARB, and APC	methylation	EZ-DNA Methylation Kit<br><br>quantitative methylation-specific polymerase chain reaction (MSP) Taq (Ab) Polymerase<br><br>internal control: ACTB negative control: Universally unmethylated DNA	cohort 1: AUC 0.69 cohort 2: AUC 0.65	logistic regression model<br><br>AUC of ROC<br><br>95% CI	cohort 1: 55%	cohort 1: 80%	cohort 1: 121 cohort 2: 113		Little Rock, Arkansas	34.746483	-92.289597	Vener et al., 2008	
87	prostate cancer	Prostate Cancer	urine								centrifuge, pellet in PBS<br><br>DNA extraction with Puregene Kit<br><br>DNA quantification:	GSTP1, RARB, and APC	methylation	EZ-DNA Methylation Kit<br><br>quantitative methylation-specific	cohort 1: AUC 0.69 cohort 2: AUC 0.65	logistic regression model<br><br>AUC of ROC	cohort 1: 55%	cohort 1: 80%	cohort 1: 121 cohort 2: 113		Rochester, Minnesota	44.016369	-92.475395	Vener et al., 2008	

											Picogreen DNAQuantification Assay			polymerase chain reaction (MSP)<br>Taq (Ab) Polymerase<br><br>internal control: ACTB<br>negative control: Universally unmethylated DNA		95% CI									
87	prostate cancer	Prostate Cancer	urine								centrifuge, pellet in PBS<br><br>DNA extraction with Puregene Kit<br><br>DNA quantification: Picogreen DNAQuantification Assay	GSTP1, RARB, and APC	methylation	EZ-DNA Methylation Kit<br><br>quantitative methylation-specific polymerase chain reaction (MSP)<br>Taq (Ab) Polymerase<br><br>internal control: ACTB<br>negative control: Universally unmethylated DNA	cohort 1: AUC 0.69<br>cohort 2: AUC 0.65	logistic regression model<br><br>AUC of ROC<br><br>95% CI	cohort 1: 55%	cohort 1: 80%	cohort 1: 121<br>cohort 2: 113		San Diego, California	32.715736	-117.161087	Vener et al., 2008	
87	prostate cancer	Prostate Cancer	urine								centrifuge, pellet in PBS<br><br>DNA extraction with Puregene Kit<br><br>DNA quantification: Picogreen DNAQuantification Assay	GSTP1, RARB, and APC	methylation	EZ-DNA Methylation Kit<br><br>quantitative methylation-specific polymerase chain reaction (MSP)<br>Taq (Ab) Polymerase<br><br>internal control: ACTB<br>negative control: Universally unmethylated DNA	cohort 1: AUC 0.69<br>cohort 2: AUC 0.65	logistic regression model<br><br>AUC of ROC<br><br>95% CI	cohort 1: 55%	cohort 1: 80%	cohort 1: 121<br>cohort 2: 113		Tucson, Arizona	32.25346	-110.911789	Vener et al., 2008	
87	prostate cancer	Prostate Cancer	urine								centrifuge, pellet in PBS<br><br>DNA extraction with Puregene Kit<br><br>DNA quantification: Picogreen DNAQuantification Assay	GSTP1, RARB, and APC	methylation	EZ-DNA Methylation Kit<br><br>quantitative methylation-specific polymerase chain reaction (MSP)<br>Taq (Ab) Polymerase	cohort 1: AUC 0.69<br>cohort 2: AUC 0.65	logistic regression model<br><br>AUC of ROC<br><br>95% CI	cohort 1: 55%	cohort 1: 80%	cohort 1: 121<br>cohort 2: 113		Snellville, Georgia	33.857327	-84.019913	Vener et al., 2008	

														internal control: ACTB<br>negative control: Universally unmethylated DNA											
87	prostate cancer	Prostate Cancer	urine								centrifuge, pellet in PBS<br><br>DNA extraction with Puregene Kit<br><br>DNA quantification: Picogreen DNAQuantification Assay	GSTP1, RARB, and APC	methylation	EZ-DNA Methylation Kit<br><br>quantitative methylation-specific polymerase chain reaction (MSP)<br>Taq (Ab) Polymerase<br><br>internal control: ACTB<br>negative control: Universally unmethylated DNA	cohort 1: AUC 0.69<br>cohort 2: AUC 0.65	logistic regression model<br><br>AUC of ROC<br><br>95% CI	cohort 1: 55%	cohort 1: 80%	cohort 1: 121<br>cohort 2: 113		Innsbruck, Austria	47.259659	11.400375	Vener et al., 2008	
87	prostate cancer	Prostate Cancer	urine								centrifuge, pellet in PBS<br><br>DNA extraction with Puregene Kit<br><br>DNA quantification: Picogreen DNAQuantification Assay	GSTP1, RARB, and APC	methylation	EZ-DNA Methylation Kit<br><br>quantitative methylation-specific polymerase chain reaction (MSP)<br>Taq (Ab) Polymerase<br><br>internal control: ACTB<br>negative control: Universally unmethylated DNA	cohort 1: AUC 0.69<br>cohort 2: AUC 0.65	logistic regression model<br><br>AUC of ROC<br><br>95% CI	cohort 1: 55%	cohort 1: 80%	cohort 1: 121<br>cohort 2: 113		Graz, Austria	47.076668	15.421371	Vener et al., 2008	
87	prostate cancer	Prostate Cancer	urine								centrifuge, pellet in PBS<br><br>DNA extraction with Puregene Kit<br><br>DNA quantification: Picogreen DNAQuantification Assay	GSTP1, RARB, and APC	methylation	EZ-DNA Methylation Kit<br><br>quantitative methylation-specific polymerase chain reaction (MSP)<br>Taq (Ab) Polymerase<br><br>internal control: ACTB<br>negative control: Universally	cohort 1: AUC 0.69<br>cohort 2: AUC 0.65	logistic regression model<br><br>AUC of ROC<br><br>95% CI	cohort 1: 55%	cohort 1: 80%	cohort 1: 121<br>cohort 2: 113		Warsaw, Poland	52.237049	21.017532	Vener et al., 2008	

														unmethylated<br>DNA											
8<br>7	prostate<br>cancer	Prostate<br>Cancer	urine								centrifuge, pellet in<br>PBS<br><br>DNA extraction with<br>Puregene Kit<br><br>DNA quantification:<br>Picogreen<br>DNAQuantification<br>Assay	GSTP1,<br>RARB,<br>and APC	methyl<br>ation	EZ-DNA<br>Methylation<br>Kit<br><br>quantitative<br>methylation-<br>specific<br>polymerase<br>chain reaction<br>(MSP)<br>Taq (Ab)<br>Polymerase<br><br>internal<br>control: ACTB<br>negative<br>control:<br>Universally<br>unmethylated<br>DNA	cohort 1:<br>AUC 0.69<br>cohort 2:<br>AUC 0.65	logistic<br>regression<br>model<br><br>AUC of<br>ROC<br><br>95% CI	cohort 2:<br>53%	cohort 2:<br>76%	cohort 1:<br>121<br>cohort 2:<br>113		Little<br>Rock,<br>Arkansas	34.74<br>6483	-<br>92.289<br>597	Vener et<br>al.,<br>2008	
8<br>7	prostate<br>cancer	Prostate<br>Cancer	urine								centrifuge, pellet in<br>PBS<br><br>DNA extraction with<br>Puregene Kit<br><br>DNA quantification:<br>Picogreen<br>DNAQuantification<br>Assay	GSTP1,<br>RARB,<br>and APC	methyl<br>ation	EZ-DNA<br>Methylation<br>Kit<br><br>quantitative<br>methylation-<br>specific<br>polymerase<br>chain reaction<br>(MSP)<br>Taq (Ab)<br>Polymerase<br><br>internal<br>control: ACTB<br>negative<br>control:<br>Universally<br>unmethylated<br>DNA	cohort 1:<br>AUC 0.69<br>cohort 2:<br>AUC 0.65	logistic<br>regression<br>model<br><br>AUC of<br>ROC<br><br>95% CI	cohort 2:<br>53%	cohort 2:<br>76%	cohort 1:<br>121<br>cohort 2:<br>113		Rocheste<br>r,<br>Minnesot<br>a	44.01<br>6369	-<br>92.475<br>395	Vener et<br>al.,<br>2008	
8<br>7	prostate<br>cancer	Prostate<br>Cancer	urine								centrifuge, pellet in<br>PBS<br><br>DNA extraction with<br>Puregene Kit<br><br>DNA quantification:<br>Picogreen<br>DNAQuantification<br>Assay	GSTP1,<br>RARB,<br>and APC	methyl<br>ation	EZ-DNA<br>Methylation<br>Kit<br><br>quantitative<br>methylation-<br>specific<br>polymerase<br>chain reaction<br>(MSP)<br>Taq (Ab)<br>Polymerase<br><br>internal<br>control: ACTB<br>negative<br>control:<br>Universally<br>unmethylated<br>DNA	cohort 1:<br>AUC 0.69<br>cohort 2:<br>AUC 0.65	logistic<br>regression<br>model<br><br>AUC of<br>ROC<br><br>95% CI	cohort 2:<br>53%	cohort 2:<br>76%	cohort 1:<br>121<br>cohort 2:<br>113		San<br>Diego,<br>California	32.71<br>5736	-<br>117.16<br>1087	Vener et<br>al.,<br>2008	
8<br>7	prostate<br>cancer	Prostate<br>Cancer	urine								centrifuge, pellet in<br>PBS<br><br>DNA extraction with	GSTP1,<br>RARB,<br>and APC	methyl<br>ation	EZ-DNA<br>Methylation<br>Kit	cohort 1:<br>AUC 0.69<br>cohort 2:<br>AUC 0.65	logistic<br>regression<br>model	cohort 2:<br>53%	cohort 2:<br>76%	cohort 1:<br>121<br>cohort 2:<br>113		Tucson,<br>Arizona	32.25<br>346	-<br>110.91<br>1789	Vener et<br>al.,<br>2008	

											Puregene Kit<br><br>DNA quantification: Picogreen DNAQuantification Assay			quantitative methylation-specific polymerase chain reaction (MSP)<br>Taq (Ab) Polymerase<br><br>internal control: ACTB<br>negative control: Universally unmethylated DNA		AUC of ROC<br><br>95% CI									
87	prostate cancer	Prostate Cancer	urine								centrifuge, pellet in PBS<br><br>DNA extraction with Puregene Kit<br><br>DNA quantification: Picogreen DNAQuantification Assay	GSTP1, RARB, and APC	methylation	EZ-DNA Methylation Kit<br><br>quantitative methylation-specific polymerase chain reaction (MSP)<br>Taq (Ab) Polymerase<br><br>internal control: ACTB<br>negative control: Universally unmethylated DNA	cohort 1: AUC 0.69<br>cohort 2: AUC 0.65	logistic regression model<br><br>AUC of ROC<br><br>95% CI	cohort 2: 53%	cohort 2: 76%	cohort 1: 121<br>cohort 2: 113		Snellville, Georgia	33.857327	-84.019913	Vener et al., 2008	
87	prostate cancer	Prostate Cancer	urine								centrifuge, pellet in PBS<br><br>DNA extraction with Puregene Kit<br><br>DNA quantification: Picogreen DNAQuantification Assay	GSTP1, RARB, and APC	methylation	EZ-DNA Methylation Kit<br><br>quantitative methylation-specific polymerase chain reaction (MSP)<br>Taq (Ab) Polymerase<br><br>internal control: ACTB<br>negative control: Universally unmethylated DNA	cohort 1: AUC 0.69<br>cohort 2: AUC 0.65	logistic regression model<br><br>AUC of ROC<br><br>95% CI	cohort 2: 53%	cohort 2: 76%	cohort 1: 121<br>cohort 2: 113		Innsbruck, Austria	47.259659	11.400375	Vener et al., 2008	
87	prostate cancer	Prostate Cancer	urine								centrifuge, pellet in PBS<br><br>DNA extraction with Puregene Kit<br><br>DNA quantification: Picogreen DNAQuantification Assay	GSTP1, RARB, and APC	methylation	EZ-DNA Methylation Kit<br><br>quantitative methylation-specific polymerase chain reaction (MSP)	cohort 1: AUC 0.69<br>cohort 2: AUC 0.65	logistic regression model<br><br>AUC of ROC<br><br>95% CI	cohort 2: 53%	cohort 2: 76%	cohort 1: 121<br>cohort 2: 113		Graz, Austria	47.076668	15.421371	Vener et al., 2008	

														Taq (Ab) Polymerase<br><br>internal control: ACTB negative control: Universally unmethylated DNA											
87	prostate cancer	Prostate Cancer	urine								centrifuge, pellet in PBS<br><br>DNA extraction with Puregene Kit<br><br>DNA quantification: Picogreen DNAQuantification Assay	GSTP1, RARB, and APC	methylation	EZ-DNA Methylation Kit<br><br>quantitative methylation-specific polymerase chain reaction (MSP)<br>Taq (Ab) Polymerase<br><br>internal control: ACTB negative control: Universally unmethylated DNA	cohort 1: AUC 0.69<br>cohort 2: AUC 0.65	logistic regression model<br><br>AUC of ROC<br><br>95% CI	cohort 2: 53%	cohort 2: 76%	cohort 1: 121<br>cohort 2: 113		Warsaw, Poland	52.237049	21.017532	Vener et al., 2008	
88	non-muscle-invasive urothelial carcinoma	Bladder Cancer	urine	25-50 mL			0.5		0.0133	500 ng of bisulfite-converted DNA	QIAamp DNA Mini Kit<br><br>concentration analysis: NanoDrop	BCL2 and hTERT promoters	methylation	bisulfite treatment Epitect Bisulfite Kit<br><br>positive control: human sperm DNA methylated using SssI DNA methylase, bisulfite treated negative control: untreated sperm DNA<br><br>methylation-specific real-time polymerase chain reaction QuantiTect Probe polymerase chain reaction Master Mix<br><br>reference gene: β-Actin	bladder tumor and urine sediment correlation BCL2 and hTERT: P < 0.001<br>BCL2 only: P = 0.005	Kolmogorov-Smirnov test<br>chi-square test<br>Student's t-test<br>Spearman's rank correlation test<br><br>receiver operating characteristic curves, area under the curve (AUC)<br><br>P < 0.05	BCL2 and hTERT: 76%	BCL2 and hTERT: 98%	108	105	Pisa, Italy<br>Florence, Italy	43.716667	10.4	Vinci et al., 2011	BCL2, hTERT, and DAPK promoters
89	colorectal cancer	Colorectal Cancer	feces	250 mg	50 uL			3		3 uL bisulfite-	centrifuged, pellet washed in PBS	SFRP2 promoter	methylation	EpiTect® Bisulfite Kit	hypermethylated: CRCs:	two sided Student's t test	CRCs: 87.0%		69 CRC patients	30	Yangzhou, Jiangsu	32.377529	119.398018	Wang & Tang, 2008	

										converted DNA	QIAamp DNA Stool Mini Kit			Fluorescence-based real-time PCR assay (MethyLight) SYBR greenIand PCR<br><br>standard universal methylated DNA	91.3% advanced adenomas: 79.4% hyperplastic polyps: 53.8%	Chi-square test Fisher exact test<br><br>P < 0.05					Province, China				
89	colorectal cancer	Colorectal Cancer	feces								centrifuged, pellet washed in PBS<br><br>QIAamp DNA Stool Mini Kit	SFRP2 promoter	methylation	EpiTect® Bisulfite Kit<br><br>Fluorescence-based real-time PCR assay (MethyLight) SYBR greenIand PCR<br><br>standard universal methylated DNA	hypermethylated: CRCs: 91.3% advanced adenomas: 79.4% hyperplastic polyps: 53.8%	two sided Student's t test Chi-square test Fisher exact test<br><br>P < 0.05	advanced adenomas: 61.8%		34 patients with adenoma ≥ 1 cm	30	Yangzhou, Jiangsu Province, China	32.377529	119.398018	Wang & Tang, 2008	
89	colorectal cancer	Colorectal Cancer	feces								centrifuged, pellet washed in PBS<br><br>QIAamp DNA Stool Mini Kit	SFRP2 promoter	methylation	EpiTect® Bisulfite Kit<br><br>Fluorescence-based real-time PCR assay (MethyLight) SYBR greenIand PCR<br><br>standard universal methylated DNA	hypermethylated: CRCs: 91.3% advanced adenomas: 79.4% hyperplastic polyps: 53.8%	two sided Student's t test Chi-square test Fisher exact test<br><br>P < 0.05	hyperplastic polyps: 42.3%	hyperplastic polyps: 76.8%	26 patients with hyperplastic polyp	30	Yangzhou, Jiangsu Province, China	32.377529	119.398018	Wang & Tang, 2008	
90	systemic lupus erythematosus (SLE)	Systemic Lupus Erythematosus (SLE)	urine								centrifuged, pellet analyzed<br><br>RNA: mirVana PARIS Kit	miR-200a, miR-200c, miR-141, miR-429 and miR-192	microRNA expression	RT-qPCR TaqMan miRNA reverse transcription kit	patients: miR-200a:18.33, miR-200c: 18.36 miR-141: 16.24 miR-429: 14.07 miR-192: 16.04<br><br>controls: miR-200a: 20.08 miR-200c: 20.45 miR-141: 17.69 miR-429:	Mann–Whitney U test Spearman's rank correlation p < 0.05			40	30	Hong Kong, China	22.302711	114.177216	Wang et al., 2011	

															16.37 miR-192: 17.74										
91	systemic lupus erythematosus (SLE)	Systemic Lupus Erythematosus (SLE)	urine								centrifuged, pellet analyzed<br><br>RNA: MirVana™ miRNA isolation kits	miR-146a and miR-155	microRNA expression	RT-qPCR TaqMan miRNA reverse transcription kit	miR-146a & glomerular filtration rate (r = 0.242, P = 0.008) miR-155 & proteinuria (r = 0.407, P < 0.001) & systemic lupus erythematosus disease activity index (r = 0.278, P = 0.002) miR-146a reversely correlated with the urinary expression of TNF-α (r = −0.247 , P = 0.012)	Mann–Whitney U test Spearman's rank correlation p < 0.05			40	13	Hong Kong, China	22.302711	114.177216	Wang et al., 2012	
92	non–muscle-invasive bladder cancer (NMIBC)	Bladder Cancer	urine	10 mL							centrifuge, pellet	15 proprietary DNA methylation biomarkers	methylation	real-time polymerase chain reaction Bladder EpiCheckTM Methylation Test	with low-grade lesions: AUC 0.82 without low-grade lesions: AUC 0.94	AUC of ROC<br><br>95% CI	68.2% PPV: 44.8%<br><br>excluding low-grade: 91.7%	88.0% NPV: 95.1%<br><br>excluding low-grade: NPV: 99.3%	353		Nijmegen, The Netherlands	51.8425	5.85278	Witjes et al., 2018	
92	non–muscle-invasive bladder cancer (NMIBC)	Bladder Cancer	urine								centrifuge, pellet	15 proprietary DNA methylation biomarkers	methylation	real-time polymerase chain reaction Bladder EpiCheckTM Methylation Test	with low-grade lesions: AUC 0.82 without low-grade lesions: AUC 0.94	AUC of ROC<br><br>95% CI	68.2% PPV: 44.8%<br><br>excluding low-grade: 91.7%	88.0% NPV: 95.1%<br><br>excluding low-grade: NPV: 99.3%	353		Barcelona, Spain	41.390205	2.154007	Witjes et al., 2018	
92	non–muscle-invasive bladder cancer (NMIBC)	Bladder Cancer	urine								centrifuge, pellet	15 proprietary DNA methylation biomarkers	methylation	real-time polymerase chain reaction Bladder EpiCheckTM Methylation Test	with low-grade lesions: AUC 0.82 without low-grade lesions: AUC 0.94	AUC of ROC<br><br>95% CI	68.2% PPV: 44.8%<br><br>excluding low-grade: 91.7%	88.0% NPV: 95.1%<br><br>excluding low-grade: NPV: 99.3%	353		Hengelo, The Netherlands	52.26583	6.79306	Witjes et al., 2018	
92	non–muscle-invasive bladder	Bladder Cancer	urine								centrifuge, pellet	15 proprietary DNA methylation	methylation	real-time polymerase chain reaction Bladder EpiCheckTM	with low-grade lesions: AUC 0.82 without	AUC of ROC<br><br>95% CI	68.2% PPV: 44.8%<br><br>excluding	88.0% NPV: 95.1%<br><br>excluding	353		Würzburg, Germany	49.783333	9.933333	Witjes et al., 2018	

	cancer (NMIBC)											biomarkers		Methylation Test	low-grade lesions: AUC 0.94		low-grade: 91.7%	low-grade: NPV: 99.3%							
92	non–muscle-invasive bladder cancer (NMIBC)	Bladder Cancer	urine								centrifuge, pellet	15 proprietary DNA methylation biomarkers	methylation	real-time polymerase chain reaction Bladder EpiCheckTM Methylation Test	with low-grade lesions: AUC 0.82 without low-grade lesions: AUC 0.94	AUC of ROC<br><br>95% CI	68.2% PPV: 44.8%<br><br>excluding low-grade: 91.7%	88.0% NPV: 95.1%<br><br>excluding low-grade: NPV: 99.3%	353		Kfar Saba, Israel	32.175	34.90694	Witjes et al., 2018	
92	non–muscle-invasive bladder cancer (NMIBC)	Bladder Cancer	urine								centrifuge, pellet	15 proprietary DNA methylation biomarkers	methylation	real-time polymerase chain reaction Bladder EpiCheckTM Methylation Test	with low-grade lesions: AUC 0.82 without low-grade lesions: AUC 0.94	AUC of ROC<br><br>95% CI	68.2% PPV: 44.8%<br><br>excluding low-grade: 91.7%	88.0% NPV: 95.1%<br><br>excluding low-grade: NPV: 99.3%	353		Sindelfingen, Germany	48.7	9.01667	Witjes et al., 2018	
93	hematuria	Hematuria	urine	30 mL			0.5		0.0167	500 ng of bisulfite-converted DNA	centrifuged, pellet washed in phosphate buffered saline<br><br>QIAamp DNA Micro Kit<br><br>concentration analysis: NanoDrop	HOXA9, PCDH17, POU4F2, and ONECUT2	methylation	bisulfite treatment EZ DNA Methylation-Gold Kit<br><br>methylation-specific high-resolution melting-curve (MS-HRM) polymerase chain reaction (PCR) ZymoTaq qPCR Premix	AUC 0.871	Mann-WhitneyUtest chi-square test<br><br>AUC of ROC<br><br>95% CI	90.5% PPV: 100%	73.2% NPV: 98%	97	95	Shanghai, China	31.224361	121.46917	Wu et al., 2020	
94	renal cell carcinoma (RCC)	Kidney Cancer	urine	20-50 mL				1		1 µl of bisulfite-converted DNA	centrifuged, pellet washed in phosphate-buffered saline<br><br>AllPrep DNA Mini kit<br><br>concentration analysis: NanoDrop	TCF21	methylation	bisulfite treatment EZ-96 DNA Methylation-Gold™<br><br>Quantitative pyrosequencing methylation analysis HotStarTaq Master Mix kit		Mann-Whitney U test Spearman's correlation<br><br>AUC of ROC<br><br>$P < 0.05$	79%	100%	33	15	Quanzhou, China	24.91389	118.58583	Xin et al., 2016	
95	colorectal cancer	Colorectal Cancer	feces								homogenization stool DNA extraction kit	SEPT9	methylation	bisulfite conversion kit<br><br>methylated SEPT9 test duplex methylated qPCR assay PCR mastermix<br><br>internal control: ACBT		$p < 0.05$ t test<br><br>Receiver operating characteristic (ROC) curves	83.30%	92.10%	100	60 paired stool and plasma samples	Xuzhou, China	34.205769	117.284126	Y. Liu et al., 2020	

96	bladder cancer	Bladder Cancer	urine				0.2			200 ng DNA	centrifuge, pellet analyzed<br><br>TIANamp Micro DNA Kit DP316<br><br>concentration analysis: Qubit™ 2.0 fluorometer<br>1% agarose gel<br>> 200 ng, size > 5 Kb	PCDH17 and POU4F2	methylation	bisulfite modification<br>EZ DNA Methylation-Gold™ Kit<br><br>positive control: CpGenome™ Universal Methylated DNA<br><br>qMSP<br>SYBR® Premix Ex TaqTM II<br><br>reference gene: ALU-C4		Mann-Whitney test<br><br>AUC or ROC<br><br>95% CI	90.00%	94%	72 urothelial cell carcinoma<br>21 infected urinary calculi<br>26 kidney cancer<br>22 prostate cancer	23 healthy individuals	He Fei Shi, An Hui Sheng, China	31.871727	117.330551	Y. Wang et al., 2016	
96	bladder cancer	Bladder Cancer	urine								centrifuge, pellet analyzed<br><br>TIANamp Micro DNA Kit DP316<br><br>concentration analysis: Qubit™ 2.0 fluorometer<br>1% agarose gel<br>> 200 ng, size > 5 Kb	PCDH17 and POU4F2	methylation	bisulfite modification<br>EZ DNA Methylation-Gold™ Kit<br><br>positive control: CpGenome™ Universal Methylated DNA<br><br>qMSP<br>SYBR® Premix Ex TaqTM II<br><br>reference gene: ALU-C4		Mann-Whitney test<br><br>AUC or ROC<br><br>95% CI	90.00%	94%	72 urothelial cell carcinoma<br>21 infected urinary calculi<br>26 kidney cancer<br>22 prostate cancer	23 healthy individuals	Guangzhou, China	23.128994	113.25325	Y. Wang et al., 2016	
96	bladder cancer	Bladder Cancer	urine								centrifuge, pellet analyzed<br><br>TIANamp Micro DNA Kit DP316<br><br>concentration analysis: Qubit™ 2.0 fluorometer<br>1% agarose gel<br>> 200 ng, size > 5 Kb	PCDH17 and POU4F2	methylation	bisulfite modification<br>EZ DNA Methylation-Gold™ Kit<br><br>positive control: CpGenome™ Universal Methylated DNA<br><br>qMSP<br>SYBR® Premix Ex TaqTM II<br><br>reference gene: ALU-C4		Mann-Whitney test<br><br>AUC or ROC<br><br>95% CI	90.00%	94%	72 urothelial cell carcinoma<br>21 infected urinary calculi<br>26 kidney cancer<br>22 prostate cancer	23 healthy individuals	Guang Zhou Shi, Guang Dong Sheng, China	23.128994	113.25325	Y. Wang et al., 2016	
97	urothelial cancer	Bladder Cancer	urine	100 mL			1		0.0100	1 ug bisulfite-treated DNA	centrifuged, pellet washed in phosphate-buffered saline	RASSF1a, APC and E-cad promoters	methylation	quantitative fluorometric real-time methylation-specific PCR assay (QMSP)<br><br>normalization gene: β-Actin		t test<br>chi-square test	RASSF1a: 0.51<br>APC: 0.4<br>E-cad: 0.31<br><br>RASSF1a, APC and E-cad panel: 0.69	RASSF1a: 0.63<br>APC: 0.83<br>E-cad: 0.53<br><br>RASSF1a, APC and E-cad	35	35 over 70 years old<br>34 under 40 years old	Sheffield, United Kingdom	53.383331	-1.466667	Yates et al., 2006	All biomarkers: RASSF1a, E-Cadherin, APC, p16, p14, GSTP, DAPK and RARb promoters

																		panel:<br>0.6							
98	bladder cancer	Bladder Cancer	urine				0.2			~200 ng bisulfite-modified DNA	ZR Urine DNA Isolation Kit	TWIST1 and NID2	methylation	bisulfite treatment<br>EZ DNA Methylation-Gold Kit<br><br>methylation-specific polymerase chain reaction (MSP)<br>ZymoTaq DNA Polymerase<br><br>positive control: Universal methylated human DNA standard		Fisher’s exact tests<br><br>P < 0.05	87.50%	95.80%	24	15	Samsun, Turkey	41.27976	36.3361	Yegin et al., 2013	
99	urothelial carcinoma	Bladder Cancer	urine	50 mL			1		0.0200	1 µg of total RNA	centrifuged, pellet washed in PBS<br><br>Genomic DNA Mini Kits	ZNF671, IRF8 and sFRP1	methylation	bisulfite treatment:<br>EZ DNA methylation kits<br><br>Quantitative MSP<br><br>reference gene: β-Actin		Mann-Whitney tests<br>Kaplan-Meier analysis<br>Cox hazard-proportional regression analysis<br><br>AUC of ROC<br><br>95% CI	42%-48%<br><br>ZNF671, IRF8 and sFRP1: 96.2%	89%-92.8%	61	28	Chia-Yi, Taiwan	47.4925	19.0513	Yeh et al., 2015	
100	bladder cancer	Bladder Cancer	urine								proteinase K/organic extraction method	SALL3, CFTR, ABCC6, HPR1, RASSF1A, MT1A, RUNX3, ITGA4, BCL2, ALX4, MYOD1, DRM, CDH13, BMP3B, CCNA1, RPRM, MINT1, and BRCA1	methylation	bisulfite treatment<br><br>MSP		Fisher’s exact test<br><br>AUC of ROC<br><br>95% CI	91.70%	87%	132	23	Nanning, Guangxi, China	22.816668	108.316666	Yu et al., 2007	
100	bladder cancer	Bladder Cancer	urine								proteinase K/organic extraction method	SALL3, CFTR, ABCC6, HPR1, RASSF1A, MT1A, RUNX3, ITGA4, BCL2,	methylation	bisulfite treatment<br><br>MSP		Fisher’s exact test<br><br>AUC of ROC<br><br>95% CI	91.70%	87%	132	23	Shanghai, China	31.224361	121.46917	Yu et al., 2007	

												ALX4,<br>MYOD1,<br>DRM,<br>CDH13,<br>BMP3B,<br>CCNA1,<br>RPRM,<br>MINT1,<br>and<br>BRCA1													
1<br>0<br>1	prostate cancer	Prostate Cancer	urine					1		1 uL<br>bisulfite-treated DNA	centrifuge, pellet in PBS	DLEC1	methyl ation	bisulfite conversion<br>EZ DNA Methylation-Direct Kit<br><br>MSP		t test<br>Fisher exact test<br>chi-square test<br><br>p <0.05	PrCa patients: 36.7%		30 prostate cancer (PrCa)	30 benign prostatic hyperpla sia (BPH)	Beijing, China	39.91 6668	116.38 3331	Zhang et al., 2015	
1<br>0<br>2	hematuri a	Hematuri a	urine		30 uL			1		1 uL DNA	QIAamp DNA Micro Kit<br><br>concentration analysis: NanoDrop	HOXA9,<br>ONECUT 2,<br>PCDH17,<br>PENK,<br>TWIST1,<br>VIM and ZNF154	methyl ation	bisulfite treatment<br>EZ DNA Methylation-Gold™ Kit<br><br>ZymoTaq™ qPCR Premix	AUC 0.894 and 0.851	Pearson’s Chi-square test<br>Fisher’s exact test<br><br>logistic regression model<br><br>AUC of ROC<br><br>95% CI			99		Shangha i, China	31.22 4361	121.46 917	Zhang et al., 2019	
1<br>0<br>3	prostate cancer	Prostate Cancer	urine	20–80 mL			0.005		0.0001	5 ng/µl DNA	centrifuged, pellet suspended in phosphate buffered saline<br><br>QiaAMP DNA Micro Kit<br><br>concentration analysis: NanoDrop	APC,<br>CRIP3,<br>GSTP1,<br>HOXD3,<br>HOXD8,<br>KLK10,<br>TBX15 and TGFb2	methyl ation	bisulfite treatment<br>EZ DNA Methylation-Gold Kit<br><br>multiplex MethyLight<br><br>normalization control: ALU	Classifier: 2.695 OR and Clinical: 2.734	Spearman ρ test<br>Mann-Whitney U test<br><br>logistic regression<br><br>95% CI			153		Toronto, Ontario, Canada	43.65 107	-79.347 015	Zhao et al., 2017	Online options are not free, sent a Report a Problem request for ILL
1<br>0<br>4	prostate cancer	Prostate Cancer	urine	50 mL	1 mL						centrifuge, pelle in PBS<br><br>AllPrep DNA/RNA mini-kit	HOXD3 and GSTP1	methyl ation	bisulfite conversion<br><br>quantitative MethyLight assay<br>Prostate Cancer Urinary Epigenetic (ProCUrE) assay<br><br>positive control: CpGenome Universal Methylated DNA<br>reference gene: ALU		Spearman ’s ρ rank chi-square tests<br><br>logistic regression<br><br>ROC<br><br>95% CI<br>C-statistic	31.6%<br>PPV: 59.4–78%	false positive: 11.9%	408		Norwich, Norfolk, England	52.63 0886	1.2973 55	Zhao et al., 2018	
1<br>0<br>4	prostate cancer	Prostate Cancer	urine								centrifuge, pelle in PBS	HOXD3 and GSTP1	methyl ation	bisulfite conversion		Spearman ’s ρ rank chi-square	31.6%<br>PPV: 59.4–78%	false positive: 11.9%	408		Toronto, Ontario, Canada	43.65 107	-79.347 015	Zhao et al., 2018	

											AllPrep DNA/RNA mini-kit			quantitative MethyLight assay<br>Prostate Cancer Urinary Epigenetic (ProCUrE) assay<br><br>positive control: CpGenome Universal Methylated DNA<br>reference gene: ALU		tests<br><br>logistic regression<br><br>ROC<br><br>95% CI<br>C-statistic									
104	prostate cancer	Prostate Cancer	urine								centrifuge, pelle in PBS<br><br>AllPrep DNA/RNA mini-kit	HOXD3 and GSTP1	methylation	bisulfite conversion<br><br>quantitative MethyLight assay<br>Prostate Cancer Urinary Epigenetic (ProCUrE) assay<br><br>positive control: CpGenome Universal Methylated DNA<br>reference gene: ALU		Spearman's ρ rank chi-square tests<br><br>logistic regression<br><br>ROC<br><br>95% CI<br>C-statistic	31.6%<br>PPV: 59.4–78%	false positive: 11.9%	408		Dublin, Ireland	53.35014	-6.266155	Zhao et al., 2018	
105	prostate cancer	Prostate Cancer	urine	20–80 mL	45 mL		0.1		0.0020	100 ng of bisulfite-converted DNA	centrifuge, pellet washed in PBS<br><br>RNA: miRNeasy Serum/Plasma kit<br>DNA: QiaAMP DNA micro kit<br><br>concentration analysis: NanoDrop	miR-24, miR-30c and CRIP3	expression<br><br>methylation	miRNA expression: miRCURY LNA<br>miRNA SYBR Green PCR system<br><br>DNA methylation: EZ DNA Methylation-Gold kit<br><br>qPCR-based MethyLight assay<br><br>normalization control: ALU-C4 (ALU) repeats	Odds ratio = 2.166<br><br>patient reclassification: AUC of 0.708	Spearman's r ranked test<br>Backward step-wise logistic regression<br>Mann-Whitney U<br><br>AUC of ROC<br><br>95% CI	81%	59.7%<br>NPV: 90.9%	103		Toronto, Ontario, Canada	43.65107	-79.347015	Zhao et al., 2019	
106	bladder cancer	Bladder Cancer	urine								Wizard Plus Minipreps DNA Purificiation System (Promega)<br>guanidine thiocyanate, resin mixing	jagged edges	methylation	bisulfite conversion<br>EpiTect Plus Bisulfite Kit<br><br>Illumina sequencing	JI-U values median: 35.8; range: 21.3–60.0<br><br>AUC: 0.75	Mann-Whitney U test<br>JI-U<br><br>95% CI			46	39	Hong Kong, China	22.302711	114.177216	Zhou et al., 2021	

107	colorectal cancer	Colorectal Cancer	feces	2 g			2			2.0 µg of bisulfite-converted DNA	centrifuged, pellet in GT buffer 1<br><br>proteinase K<br><br>concentration analysis: Qubit dsDNA BR assay kit	SDC2	methylation	bisulfite treatment EZ DNA Methylation-Gold kit<br><br>linear target enrichment (LTE)-quantitative methylation-specific real-time PCR (qMSP) AptaTaq PCR master mix	AUC of 0.902	AUC of ROC<br><br>95% CI	90.2%<br><br>early stages: 89.1%	90.20%	62	245	Seoul, South Korea	37.5326	127.024612	Han et al., 2019	
108	colorectal cancer	Colorectal Cancer	feces				2			2 µg DNA	e QiaAmp DNA Stool mini-kit	SFRP2, GATA4/5, NDRG4 and VIM	methylation	bisulfite treatment EpiTect Bisulfite Kit<br><br>Methylation-specific polymerase chain reaction 2.5% agarose gel, UV	Promoter methylation levels SFRP2: 57.1% GATA4/5: 42.9%, 83.9% NDRG4: 28.6% VIM: 41.1%	chi-square test Fisher's exact tests<br><br>AUC of ROC<br><br>95% CI	at least one methylated gene: 96.4%	at least one methylated gene: 65%	56	40	Ningbo, China	29.868336	121.543991	Lu et al., 2014	
109	colorectal cancer	Colorectal Cancer	feces					2		2 µL DNA	Stool DNA Extraction kit	NDRG4	methylation	bisulfite treatment EZ DNA Methylation TM-Direct kit<br><br>n-MSP Taq Polymerase			positive detection: feces: 76.2%		76	16	Beijing, China	39.916668	116.383331	Xiao et al., 2015	
109	colorectal cancer	Colorectal Cancer	urine								Stool DNA Extraction kit	NDRG4	methylation	bisulfite treatment EZ DNA Methylation TM-Direct kit<br><br>n-MSP Taq Polymerase			positive detection: urine: 72.6%		76	16	Beijing, China	39.916668	116.383331	Xiao et al., 2015	
